# Supplementary material for: Molecular mechanisms underlying the role of the centriolar CEP164-TTBK2 complex in ciliopathies
Source: Structure. 2022 Jan 6;30(1):114–128.e9. doi: 10.1016/j.str.2021.08.007 (PMC8752127; doi:10.1016/j.str.2021.08.007)
Supplement: Document S1. Figures S1–S7 [file mmc1.pdf]

Structure, Volume *30*

Supplemental Information

Molecular mechanisms underlying the role of the centriolar CEP164-TTBK2 complex in ciliopathies

Ivan Rosa e Silva, Lucia Binó, Christopher M. Johnson, Trevor J. Rutherford, David Neuhaus, Antonina Andreeva, Lukáš Čajánek, and Mark van Breugel

Supplementary Figure 1. Key residues in the CEP164-TTBK2 interface are essential for the CEP164-TTBK2 interaction. A) The N-terminal domain of CEP164 and TTBK2$^{1074\text{-}1087}$ are sufficient for the CEP164-TTBK2 interaction. Western blot showing a pull-down experiment with recombinant GST or GST-CEP164$^{1\text{-}109}$ and lysates from cells expressing C-terminally GFP-tagged TTBK2$^{1074\text{-}1087}$ constructs. TTBK2 constructs carried alanine mutations of four consecutive residues in the 1074-1087 residue range, as indicated. Below the blot, the band intensities of the eluates (in percent of the WT level) are shown (±standard deviation, n=3). **B)** Interface residues of the CEP164 WW domain are critical for its interaction with TTBK2. Western blot showing a pull-down experiment with lysates from cells expressing 3xHA tagged TTBK2 and 3xFLAG tagged CEP164, WT or the indicated WW domain mutants. Below the blot, the band intensities of the eluates (in percent of the WT level) are shown (±standard deviation, n=3). **C)** TTBK2 residues 1076-1078 are crucial for its interaction with CEP164. Western blot showing a pull-down experiment with lysates from cells expressing 3xFLAG tagged CEP164 and 3xHA tagged TTBK2, WT or R1076A+P1077A+P1078A (3-5A) mutant. TCL: Total cell lysate. **D)** The recombinant CEP164$^{1\text{-}109}$ mutants assayed for TTBK2$^{1074\text{-}1087}$ binding in ITC experiments (Figure 1C) do not strongly compromise the CEP164$^{1\text{-}109}$ secondary structure. Buffer-subtracted circular dichroism (CD) spectra of recombinant CEP164$^{1\text{-}109}$, WT and mutants at 25 °C. **E-H)** Coomassie blue-stained 4-12% NuPAGE (Invitrogen) SDS-PAGE gels of **E)** purified, recombinant Nanobodies 10Z, 36Z and the TTBK2$^{1074\text{-}1087}$-CEP164$^{1\text{-}109}$ fusion - Nanobody 36Z complex used for crystallization, **F)** purified, recombinant GST or GST-CEP164$^{1\text{-}109}$ (GST-CEP164-NTD), wild-type (WT) and mutants, as indicated above the gel, that were used in the pull-down experiments in Figure 3B, **G)** purified, recombinant GST or GST-CEP164-NTD, WT, Q11P or R93W that were used in the pull-down experiments in Figure 3F, **H)** purified, recombinant CEP164$^{1\text{-}109}$ (CEP164-NTD), WT and mutants, used in ITC experiments (Figure 1C), **I)** SDS-PAGE (home-made, 15%) gel showing purified, recombinant CEP164$^{1\text{-}109}$-ΔWW and CEP164$^{1\text{-}109}$-ΔHelices that were used in the CD experiments in Figure 2D.

Supplementary Figure 2. The high-resolution structures obtained from two different nanobody-CEP164$^{1\text{-}109}$ complexes are highly similar. A) Top: Ribbon representation of the structure of CEP164$^{1\text{-}109}$, rainbow-coloured from N- to C-terminus, in complex with the camelid nanobody 10Z (in grey). Rotation, as indicated. Consecutive alpha-helices (α), beta-sheets (β) and linkers (L) are labelled. Dotted boxes indicate the regions showed magnified in the panels below. Bottom left: Detailed view of the CEP164$^{1\text{-}109}$ WW domain region including the short β1 strand. Bottom right: Detailed view of the interface region between the CDR3 loop of the bound nanobody and CEP164$^{1\text{-}109}$. Green or yellow dotted lines indicate hydrogen-bonds. Selected residues are shown as sticks and are labelled. **B)** Equivalent views to A) but with the structure of the CEP164$^{1\text{-}109}$-nanobody 36Z complex. Note that despite CDR3 loops of different length and sequence, the binding modes of nanobody 10Z and 36Z are similar to each other. Both nanobodies utilise an arginine in their CDR3 loops to engage CEP164 residue D89. CEP164 D89 is located in the interface region between the WW domain and the helical bundle of CEP164's N-terminal domain.

Supplementary Figure 3. NMR analyses of CEP164$^{1\text{-}109}$ alone and in complex with TTBK2$^{1074\text{-}1087}$ in solution. A) Amino acid residues 1-25 of CEP164$^{1\text{-}109}$ are flexible in solution. Top: {^{1}H} ^{15}N heteronuclear NOE (mean from duplicate datasets) plotted by residue number for CEP164$^{1\text{-}109}$ wild-type (WT, blue circles) and Q11P mutant (red

squares), depicting the flexibility in the CEP164$^{1-109}$ backbone at 293 K. The observed WT - Q11P difference for each residue is shown in black bars. The dotted line across the plot indicates an (arbitrary) threshold with values above indicating conformationally more constrained and values below indicating more flexible backbones. Bottom: RCI-S^2 predicted order parameters obtained from NMR backbone chemical shifts of wild-type CEP164$^{1-109}$ using the TALOS+ software package. RCI-S^2 closely correlates with amplitudes of backbone motions in proteins (Berjanskii and Wishart, 2005). Right: Ribbon presentation of the crystal structure of CEP164$^{1-109}$ (in grey. Residues 1 and 104-109 were not visible in the electron density map, probably due to their high flexibility), consecutive alpha-helices (α), beta-sheets (β) and linkers (L) are labelled. Coloured from green through to red are residues with the specified ranges of ^{1}H ^{15}N heteronuclear NOE (I/I_0) values indicating increased flexibility of the corresponding backbones. **B)** TTBK2$^{1074-1087}$ binding results in only small conformational changes in the N-terminal domain of CEP164. Ribbon representation of the structure of CEP164$^{1-109}$ (in red) overlayed with the structure of the CEP164$^{1-109}$-TTBK2$^{1074-1087}$ complex (in green and blue). Consecutive alpha-helices (α), beta-sheets (β) and linkers (L) of CEP164$^{1-109}$ are labelled. **C)** TTBK2$^{1074-1087}$ binding to CEP164$^{1-109}$ involves the same CEP164 regions *in crystallo* and in solution. Heatmap depicting the extent of exchange broadening in the HSQC of 90 μM ^{15}N-CEP164$^{1-109}$ upon addition of 60 μM unlabelled TTBK2$^{1074-1087}$ at 293 K. The percentage attenuation of peak height for each residue, relative to wild-type, is colour-coded onto the crystal structure of CEP164$^{1-109}$ using a colour gradient from yellow (no peak height attenuation upon TTBK2$^{1074-1087}$ addition), through orange, to red (100% reduction of peak height). Proline residues, which do not produce ^{1}H-^{15}N HSQC peaks, are coloured in grey. The CEP164$^{1-109}$ structure is shown in the same orientation as in B) but in a combined ribbon and semi-transparent surface presentation. Rotation, as indicated. Note the agreement between the CEP164 regions that show exchange broadening upon TTBK2 binding in solution and those *in crystallo* that either are directly involved in TTBK2 binding or undergo conformational changes upon binding (compare to B)).

Supplementary Figure 4. Evolutionary conservation of the solvent exposed WW domain surface and the interior of the CEP164$^{1-109}$ helical bundle. Top: Ribbon representation of the structure of CEP164$^{1-109}$ coloured according to ConSurf (Ashkenazy et al., 2016) conservation scores from variable (cyan) to conserved (burgundy). Consecutive alpha-helices (α), beta-sheets (β) and linkers (L) are labelled. Rotation, as indicated. Dotted boxes indicate the regions showed magnified in the panels below. Bottom left: Detailed view of the solvent exposed CEP164$^{1-109}$ WW domain surface comprising strands β1-β4. Bottom right: Detailed view of the helical bundle region of CEP164$^{1-109}$. Selected residues are shown as sticks and are labelled.

Supplementary Figure 5. Structural comparison of the CEP164$^{1-109}$-TTBK2$^{1074-1087}$ complex with the WW domain-proline-rich binding motif (PRBM) complexes of different WW domain classes. A) View onto the solvent exposed, PRBM-interacting surface of the WW domains of CEP164$^{1-109}$ (green). Left: As ribbon representation and in complex with TTBK2$^{1074-1087}$ (grey). Right: as molecular surface representation. Circled are the XP and the XP2 grooves into which TTBK2 P1077-P1078 and P1075 insert, respectively. **B)** Similar view as in A) but with the WW domain-PRBM complexes of the

indicated members of WW domain classes I, II, III and IV, in ribbon representation. Selected residues are labelled and shown as sticks.

Supplementary Figure 6. The ciliopathic mutation R93W destabilises the CEP164$^{1-109}$ structure. CEP164 contains long regions of predicted disorder.
A) BEST-TROSY NMR spectrum of ^{15}N-labelled CEP164$^{1-109}$ R93W (red) overlayed onto the corresponding wild-type (WT) spectrum (black). Spectra were acquired at a sample temperature of 277 K (left) or 298 K (right). Extensive loss of peaks due to exchange broadening for R93W but not WT at the higher temperature illustrates the structural instability of the mutant. Even at 4°C, perturbations of the TROSY spectrum of the mutant are indicative of a structure that is already compromised. Exchange broadening and peak loss were reversible on re-cooling. **B)** The region between the N-terminal domain and the first coiled coil domain of CEP164 is predicted to be largely unstructured. Per-residue disorder probabilities of CEP164 as calculated by the disorder prediction algorithm AUCpreD (Wang et al., 2016). Values above 0.5 (dashed line) indicate disorder. The location of the N-terminal domain and the predicted coiled coil domains (cc) of CEP164 are indicated above the plot.

Supplementary Figure 7. CEP164 binding inhibits EB1 engagement by TTBK2 and does not stimulate TTBK2's autophosphorylation activity. A) CEP164-NTD binding inhibits full-length TTBK2 association with EB1. Western blot showing a pull-down experiment with bead-bound 3xFLAG or 3xFLAG-EB1 and cell lysates containing 3xHA-tagged full-length TTBK2 (WT or CEP164 binding-deficient ΔPro-rich (Δ1074-1087) mutant), in the presence of recombinant TTBK2-binding-competent WT or -incompetent Y73A GST-CEP164$^{1-109}$, as indicated. TCL: Total cell lysate. **B)** Excess EB1-GFP does not efficiently compete with CEP164$^{1-109}$ (CEP164-NTD) binding to MBP-TTBK2$^{1033-1087}$. Coomassie-stained SDS-PAGE gel showing pull-down experiments with purified MBP-TTBK2$^{1033-1087}$ (wild-type, EB1-binding deficient TTBK2$^{1051-1054\ \mathrm{SKIP\ to\ AAAA}}$ or CEP164-NTD-binding deficient TTBK2$^{1076-1078\ \mathrm{RPP\ to\ AAA}}$) with CEP164$^{1-109}$ (CEP164-NTD) in the presence of an excess of EB1-GFP. **C)** TTBK2 kinase activity is not increased in the presence of an excess of CEP164$^{1-109}$. Coomassie stained SDS-PAGE gel showing a TTBK2 autophosphorylation assay with recombinantly produced TTBK2 in the presence of CEP164-NTD WT or Y73A. Autophosphorylation of wild-type TTBK2, but not its kinase dead D163A mutant, is evident from the slowed TTBK2 migration in Coomassie-stained 8%, low bis-acrylamide SDS-PAGE gels. CEP164-NTD runs with the dye front.

Figure S1

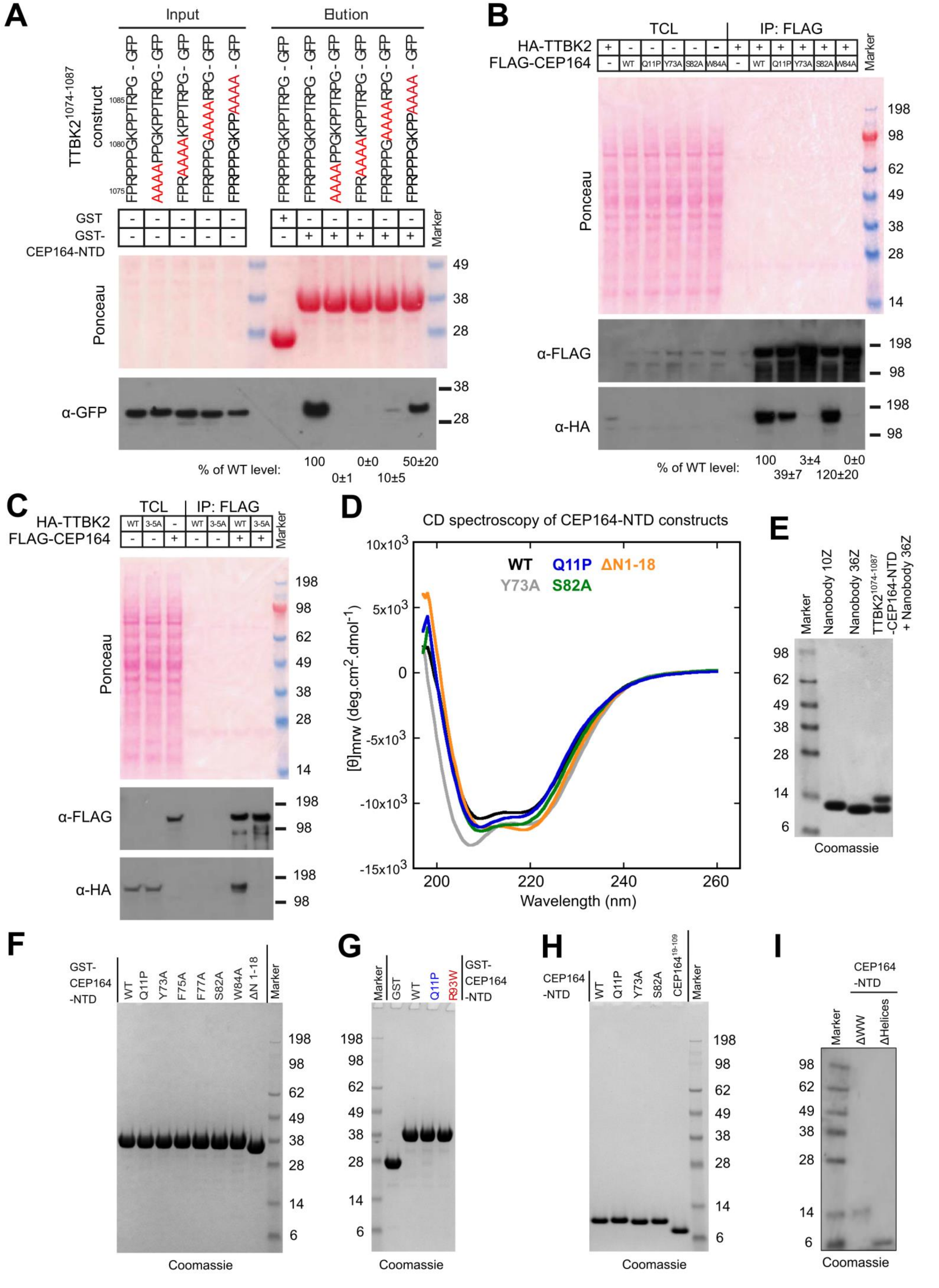
A
Input
Elution
TTBK2^1074-1087 construct
GST
GST-CEP164-NTD
Marker
Ponceau
α-GFP
% of WT level:
100
0±1
0±0
10±5
50±20
B
TCL
IP: FLAG
HA-TTBK2
FLAG-CEP164
WT
Q11P
Y73A
S82A
W84A
Ponceau
α-FLAG
α-HA
% of WT level:
100
39±7
3±4
120±20
0±0
C
TCL
IP: FLAG
HA-TTBK2
FLAG-CEP164
3-5A
Ponceau
α-FLAG
α-HA
D
CD spectroscopy of CEP164-NTD constructs
WT
Q11P
ΔN1-18
Y73A
S82A
[θ]mrw (deg.cm².dmol⁻¹)
Wavelength (nm)
E
Marker
Nanobody 10Z
Nanobody 36Z
TTBK2^1074-1087-CEP164-NTD + Nanobody 36Z
Coomassie
F
GST-CEP164-NTD
WT
Q11P
Y73A
F75A
F77A
S82A
W84A
ΔN 1-18
Marker
Coomassie
G
Marker
GST
WT
Q11P
R93W
GST-CEP164-NTD
Coomassie
H
CEP164-NTD
WT
Q11P
Y73A
S82A
CEP164^19-109
Marker
Coomassie
I
CEP164-NTD
Marker
ΔWW
ΔHelices
Coomassie

Figure S2

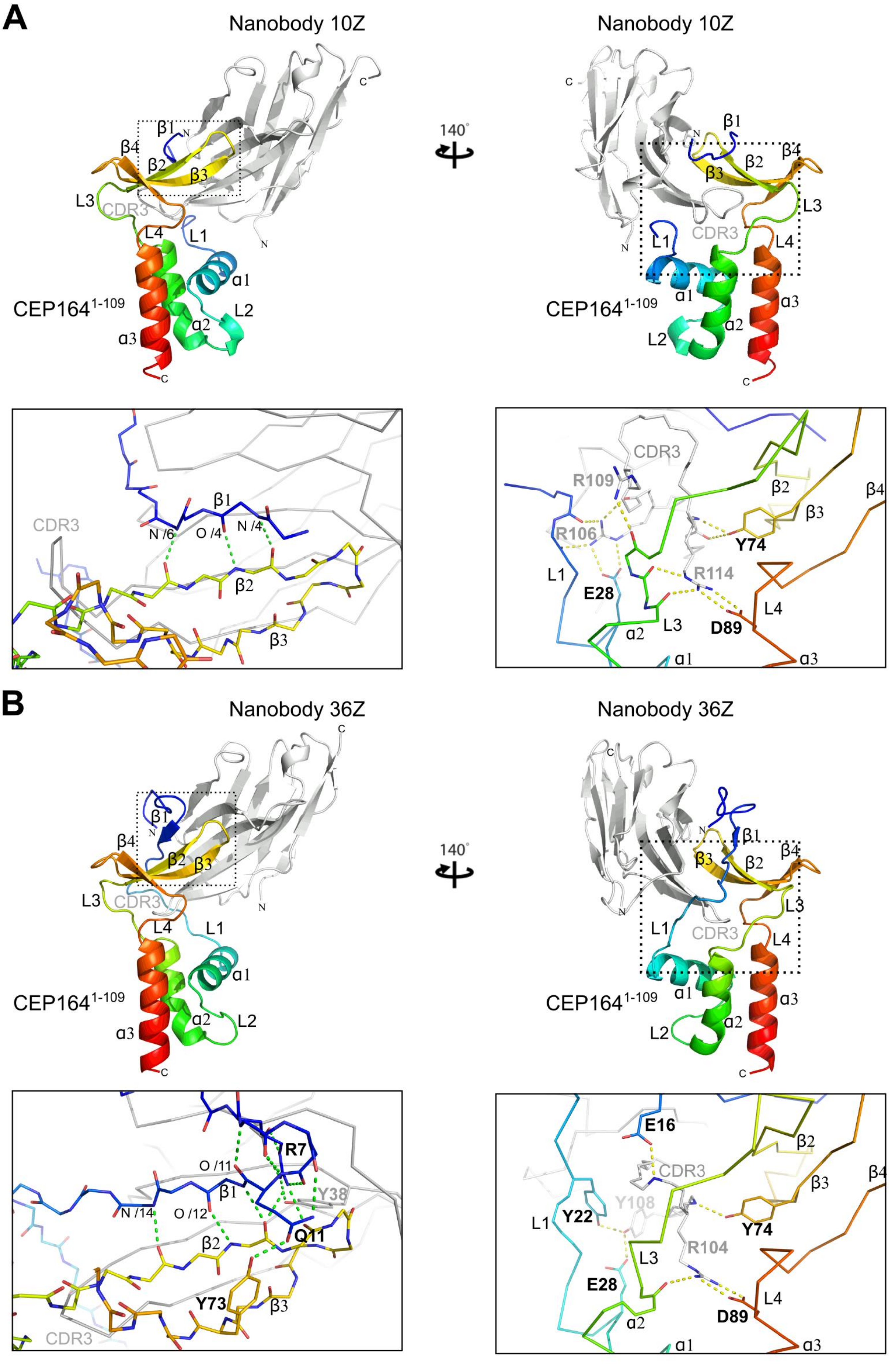

A
Nanobody 10Z
Nanobody 10Z
140°
CEP164$^{1-109}$
CEP164$^{1-109}$
β1
β2
β3
β4
L1
L2
L3
L4
α1
α2
α3
CDR3
N
C
N /6
O /4
N /4
R109
R106
R114
E28
Y74
D89
B
Nanobody 36Z
Nanobody 36Z
140°
CEP164$^{1-109}$
CEP164$^{1-109}$
O /11
N /14
O /12
R7
Y38
Q11
Y73
E16
Y22
Y108
R104
E28
Y74
D89

Figure S3

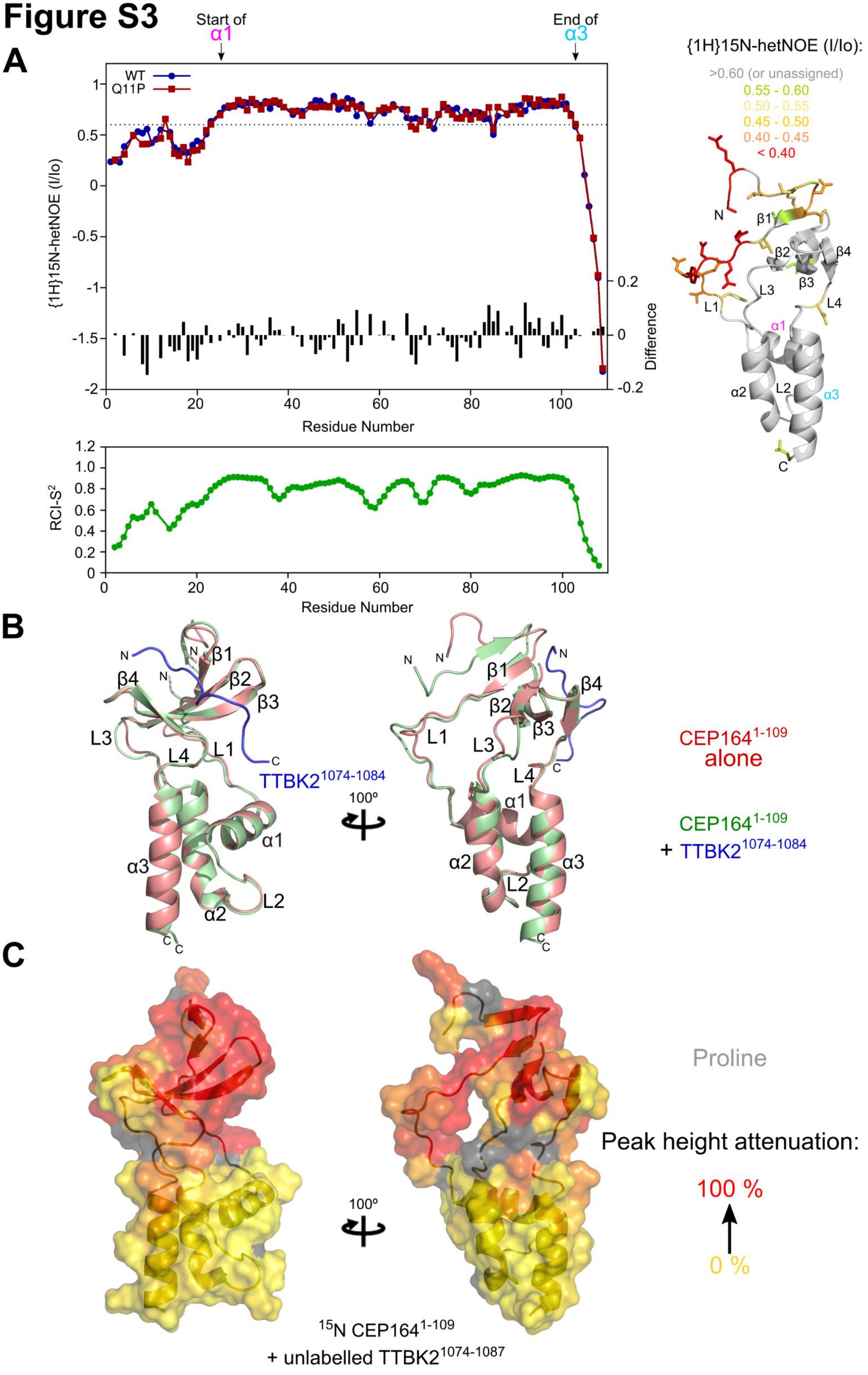
A
Start of α1
End of α3
WT
Q11P
{1H}15N-hetNOE (I/Io)
Difference
Residue Number
RCI-S²
{1H}15N-hetNOE (I/Io):
>0.60 (or unassigned)
0.55 - 0.60
0.50 - 0.55
0.45 - 0.50
0.40 - 0.45
< 0.40
N
β1
β2
β3
β4
L1
L3
L4
α1
α2
L2
α3
C
B
TTBK2$^{1074-1084}$
100º
CEP164$^{1-109}$ alone
CEP164$^{1-109}$ + TTBK2$^{1074-1084}$
C
Proline
Peak height attenuation:
100 %
0 %
^{15}N CEP164$^{1-109}$ + unlabelled TTBK2$^{1074-1087}$

Figure S4

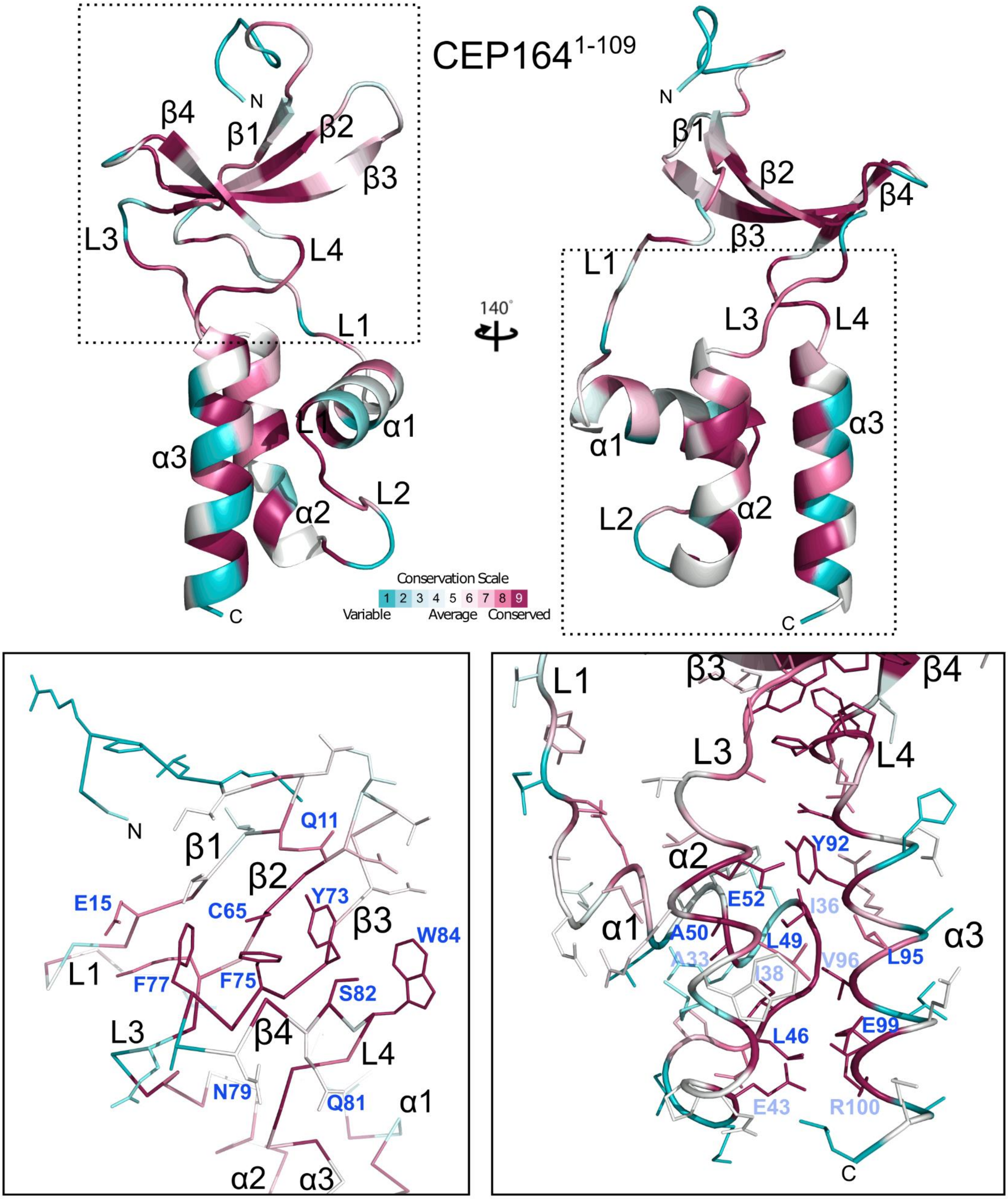
CEP164 1-109
N
β1
β2
β3
β4
L1
L2
L3
L4
α1
α2
α3
C
140°
Conservation Scale
1 2 3 4 5 6 7 8 9
Variable
Average
Conserved
Q11
E15
C65
Y73
W84
F77
F75
S82
N79
Q81
Y92
E52
I36
A50
L49
A33
I38
V96
L95
E99
L46
E43
R100

Figure S5

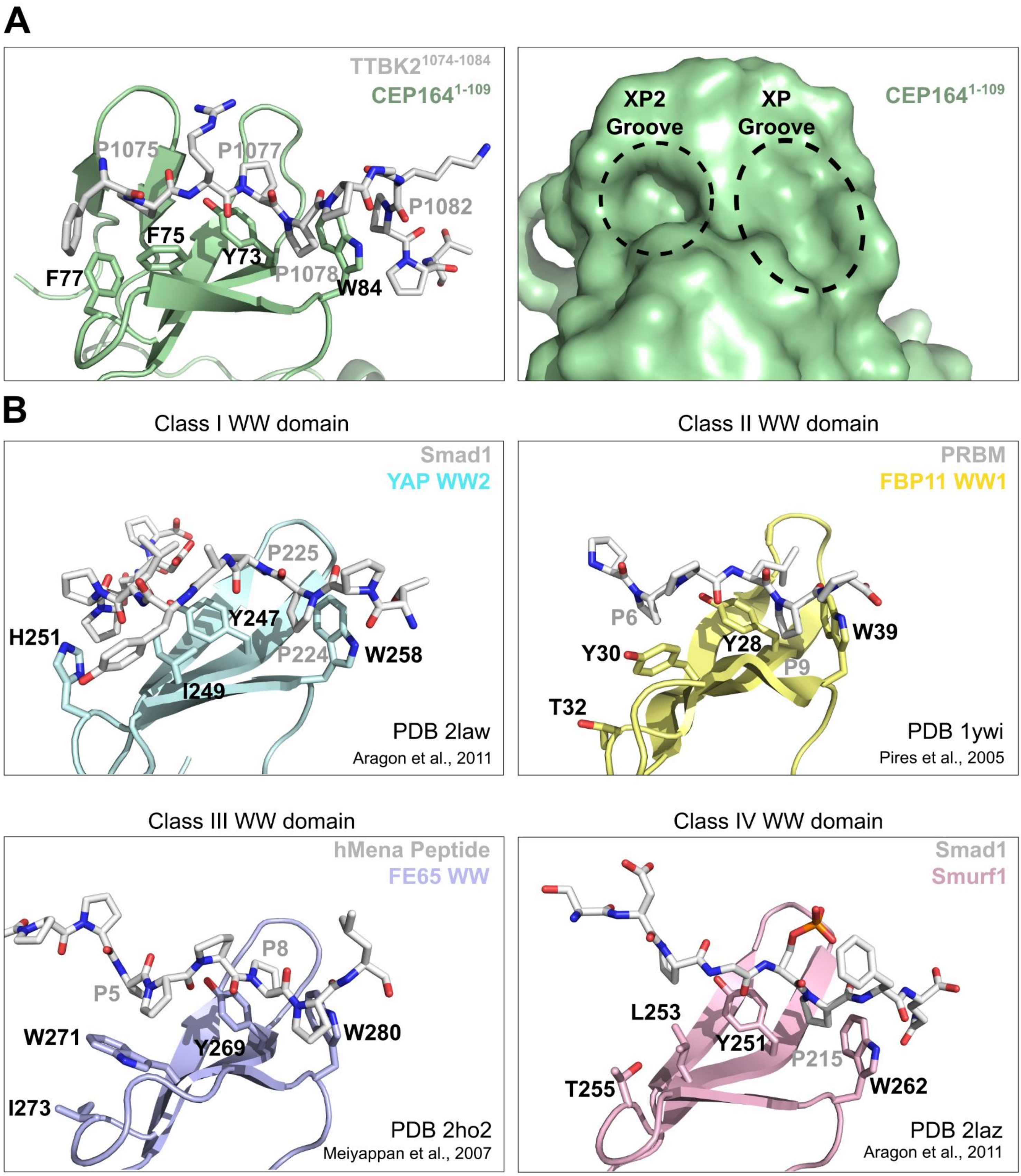

A
TTBK2$^{1074-1084}$
CEP164$^{1-109}$
P1075
P1077
P1082
F75
Y73
F77
P1078
W84
XP2 Groove
XP Groove
CEP164$^{1-109}$
B
Class I WW domain
Smad1
YAP WW2
P225
Y247
H251
P224
W258
I249
PDB 2law
Aragon et al., 2011
Class II WW domain
PRBM
FBP11 WW1
P6
W39
Y30
Y28
P9
T32
PDB 1ywi
Pires et al., 2005
Class III WW domain
hMena Peptide
FE65 WW
P8
P5
W271
Y269
W280
I273
PDB 2ho2
Meiyappan et al., 2007
Class IV WW domain
Smad1
Smurf1
L253
Y251
P215
T255
W262
PDB 2laz
Aragon et al., 2011

Figure S6

A

NMR BEST-TROSY spectra of ^{15}N labelled CEP164$^{1\text{-}109}$

277K (4 ºC) | 298K (25 ºC)

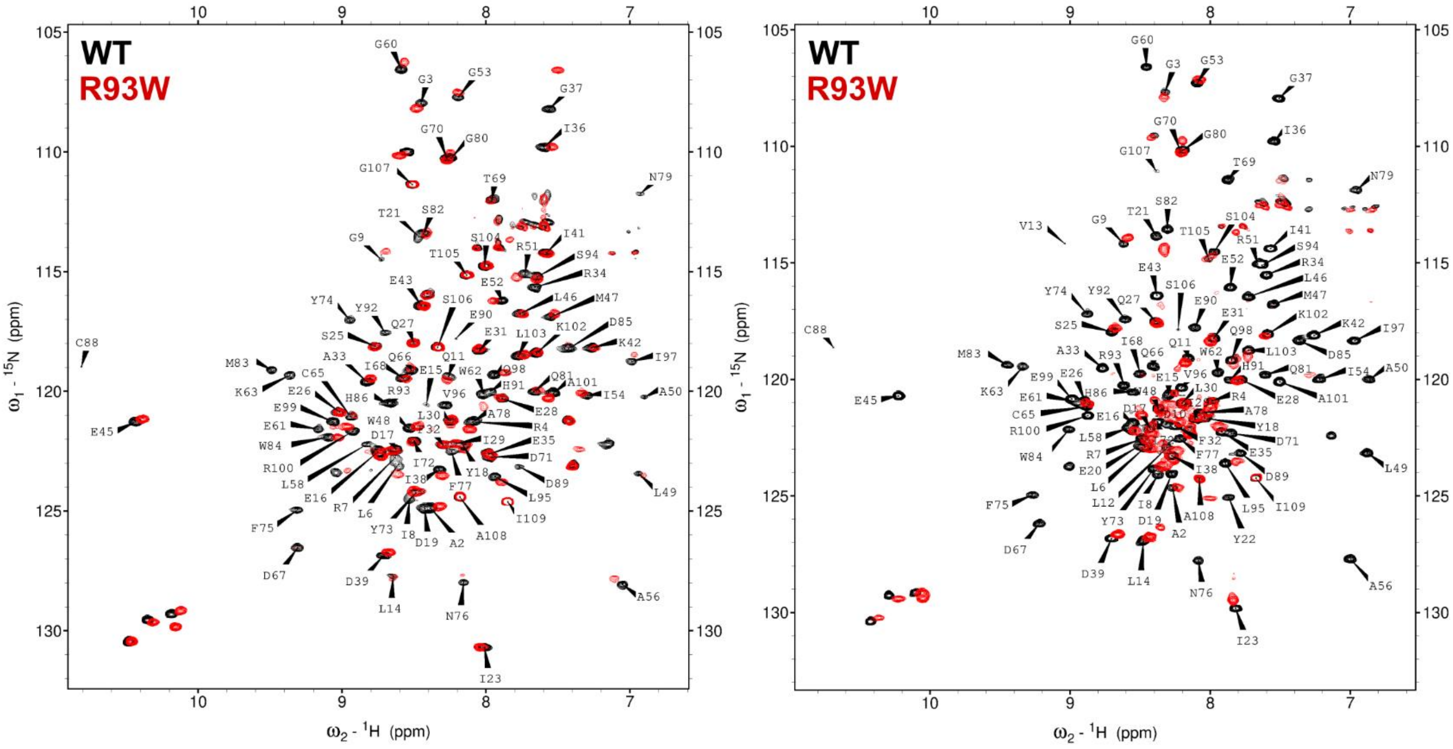

B

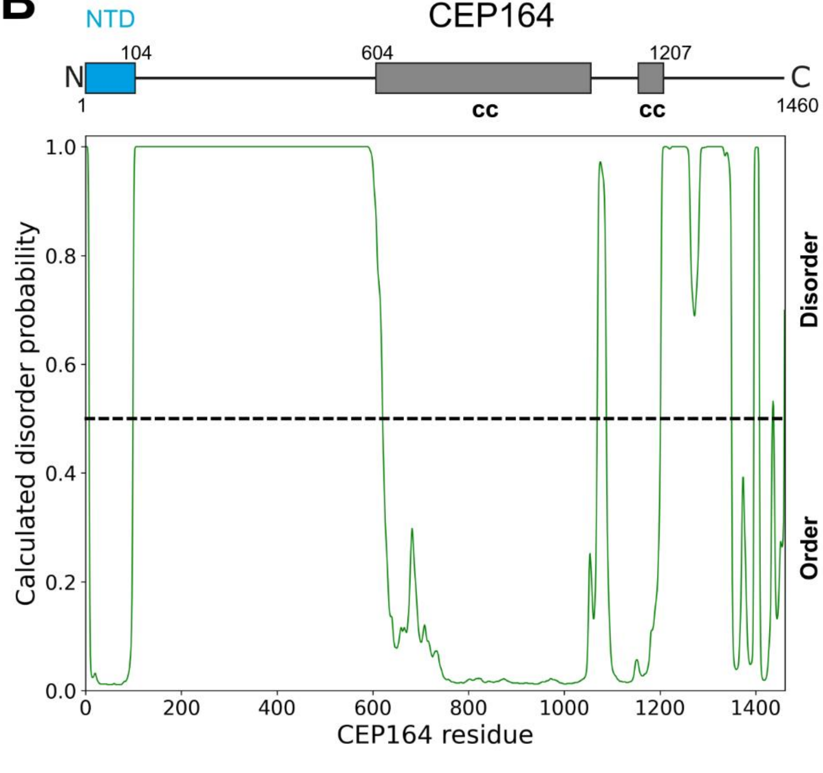

Figure S7

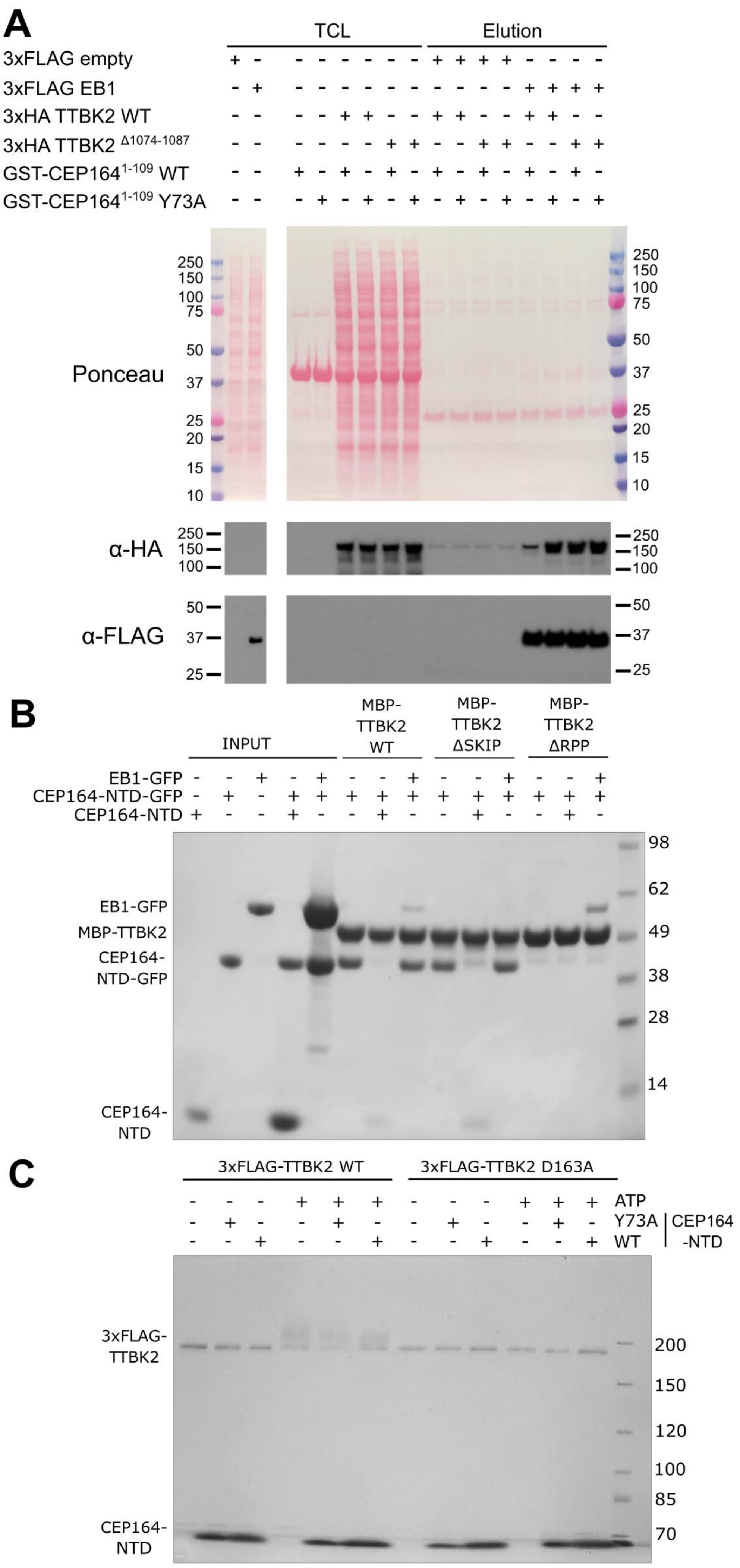

A
TCL
Elution
3xFLAG empty
3xFLAG EB1
3xHA TTBK2 WT
3xHA TTBK2 Δ1074-1087
GST-CEP164 1-109 WT
GST-CEP164 1-109 Y73A
Ponceau
α-HA
α-FLAG
250
150
100
75
50
37
25
20
15
10
B
INPUT
MBP-TTBK2 WT
MBP-TTBK2 ΔSKIP
MBP-TTBK2 ΔRPP
EB1-GFP
CEP164-NTD-GFP
CEP164-NTD
MBP-TTBK2
98
62
49
38
28
14
C
3xFLAG-TTBK2 WT
3xFLAG-TTBK2 D163A
ATP
Y73A
WT
CEP164 -NTD
3xFLAG-TTBK2
200
150
120
100
85
70
