# Supplementary material for: Molecular mechanisms underlying the role of the centriolar CEP164-TTBK2 complex in ciliopathies
Source: Structure. 2022 Jan 6;30(1):114–128.e9. doi: 10.1016/j.str.2021.08.007 (PMC8752127; doi:10.1016/j.str.2021.08.007)
Supplement: Document S2. Article plus supplemental information [file mmc2.pdf]

Structure

Article

Molecular mechanisms underlying the role of the centriolar CEP164-TTBK2 complex in ciliopathies

Graphical abstract

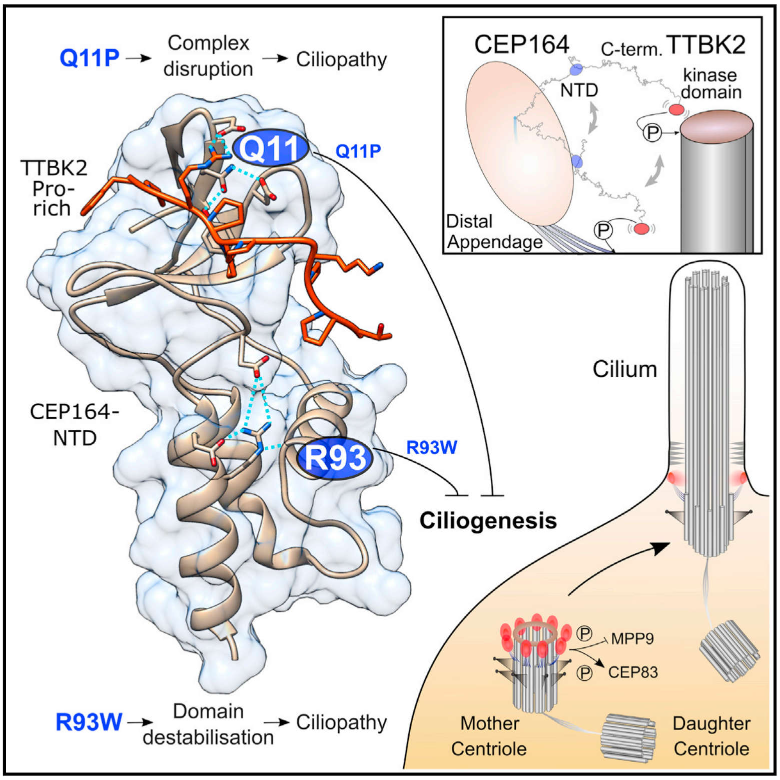

Highlights

- CEP164-NTD contains a TTBK2-binding WW domain inserted into an α-helical bundle
- CEP164-Q11P and R93W mutants cause ciliopathies by disrupting complex formation
- CEP164-NTD binding does not stimulate TTBK2 activity but impairs its EB1 engagement
- Disordered regions in TTBK2 and CEP164 might facilitate substrate access by TTBK2

Authors

Ivan Rosa e Silva, Lucia Binó, Christopher M. Johnson, ..., Antonina Andreeva, Lukáš Čajánek, Mark van Breugel

Correspondence

m.vanbreugel@qmul.ac.uk (M.v.B.), ivan.silva@alumni.usp.br (I.R.e.S.)

In brief

Rosa e Silva et al. report the structural characterization of the centriolar CEP164-TTBK2 complex that is essential for cilia formation. The authors show how ciliopathic mutations in CEP164 compromise complex formation and shed light on how CEP164 binding is coordinated with TTBK2 activities such as substrate access and EB1/3 association.

Rosa e Silva et al., 2022, Structure 30, 114–128
January 6, 2022 © 2021 The Author(s). Published by Elsevier Ltd.
https://doi.org/10.1016/j.str.2021.08.007

CellPress

CellPress
OPEN ACCESS

Structure

Article

Molecular mechanisms underlying the role of the centriolar CEP164-TTBK2 complex in ciliopathies

Ivan Rosa e Silva,[1,2,*] Lucia Binó,[3] Christopher M. Johnson,[2] Trevor J. Rutherford,[2] David Neuhaus,[2] Antonina Andreeva,[2] Lukáš Čajánek,[3] and Mark van Breugel[1,2,4,*]
[1]Queen Mary University of London, School of Biological and Chemical Sciences, 2 Newark Street, London E1 2AT, UK
[2]Medical Research Council – Laboratory of Molecular Biology, Francis Crick Avenue, Cambridge CB2 0QH, UK
[3]Department of Histology and Embryology, Faculty of Medicine, Masaryk University, Kamenice 5, Brno 62500, Czech Republic
[4]Lead contact
*Correspondence: m.vanbreugel@qmul.ac.uk (M.v.B.), ivan.silva@alumni.usp.br (I.R.e.S.)
https://doi.org/10.1016/j.str.2021.08.007

SUMMARY

Cilia formation is essential for human life. One of the earliest events in the ciliogenesis program is the recruitment of tau-tubulin kinase 2 (TTBK2) by the centriole distal appendage component CEP164. Due to the lack of high-resolution structural information on this complex, it is unclear how it is affected in human ciliopathies such as nephronophthisis. Furthermore, it is poorly understood if binding to CEP164 influences TTBK2 activities. Here, we present a detailed biochemical, structural, and functional analysis of the CEP164-TTBK2 complex and demonstrate how it is compromised by two ciliopathic mutations in CEP164. Moreover, we also provide insights into how binding to CEP164 is coordinated with TTBK2 activities. Together, our data deepen our understanding of a crucial step in cilia formation and will inform future studies aimed at restoring CEP164 functionality in a debilitating human ciliopathy.

INTRODUCTION

Cilia are hair-like cell projections that extend outward from the cell surface. They are found on most human cell types and are strictly required for human life (Bettencourt-Dias et al., 2011; Ishikawa and Marshall, 2011; Werner et al., 2017). Cilia are involved in cell motility and fluid flow generation. They also act as sensors that respond to physical and chemical stimulation and thereby play crucial roles in critical signaling pathways during development and in tissue homeostasis (Bettencourt-Dias et al., 2011; Ishikawa and Marshall, 2011; Singla and Reiter, 2006; Werner et al., 2017).

Centrioles are cylindrical cell organelles that are typically found as a pair in the center of centrosomes in interphase cells (Joukov and De Nicolo, 2019; Werner et al., 2017). The two centrioles differ in age, with the older (mother) centriole distinguished from the younger (daughter) centriole by the presence of distal and subdistal appendages (Bowler et al., 2019; Chong et al., 2020; Tanos et al., 2013; Yang et al., 2018). During cilia formation, mother centrioles become basal bodies that template cilia through the elongation of the plus ends of the doublet microtubules that constitute parts of their wall structure (Ishikawa and Marshall, 2011; Pedersen et al., 2008; Werner et al., 2017). The distal appendages play crucial roles during ciliogenesis by mediating membrane docking and providing a platform on which essential steps in cilia formation take place (Kumar and Reiter, 2021).

The initiation of ciliogenesis is tightly regulated both temporally and spatially (Werner et al., 2017; Kumar and Reiter, 2021). One of the earliest steps in cilia formation is the recruitment of tau-tubulin kinase 2 (TTBK2) to the distal appendages of centrioles through CEP164 that is located at their tip structure (Bowler et al., 2019; Cajanek and Nigg, 2014; Oda et al., 2014; Goetz et al., 2012; Yang et al., 2018). TTBK2 recruitment requires a conserved WW domain in the N-terminal region of CEP164 and a proline-rich region in TTBK2 (Cajanek and Nigg, 2014; Oda et al., 2014; Schmidt et al., 2012). CEP164-TTBK2 complex formation facilitates TTBK2-mediated phosphorylation of both CEP83 at the base of distal appendages (Bernatik et al., 2020; Lo et al., 2019) and MPHOSP9 (MPP9) at the distal end of the mother centriole (Huang et al., 2018). As a consequence, MPP9 and the associated CP110-CEP97 complex that caps the distal ends of mother centrioles are removed, promoting ciliary axoneme growth (Huang et al., 2018).

In agreement with this model, lack of CEP164 or TTBK2 impairs ciliogenesis in mammalian tissue culture cells and mouse models without otherwise affecting centriole structure (Bowie and Goetz, 2020; Cajanek and Nigg, 2014; Goetz et al., 2012; Graser et al., 2007; Humbert et al., 2012; Kobayashi et al., 2020; Lo et al., 2019; Oda et al., 2014; Schmidt et al., 2012; Slaats et al., 2014). Furthermore, CEP164 or TTBK2 truncations that lack their respective TTBK2- or CEP164-interacting region do not support ciliogenesis *in vivo* (Bowie et al., 2018; Cajanek and Nigg, 2014; Goetz et al., 2012; Kobayashi et al., 2020; Oda et al., 2014). Consistently, overexpression of the WW domain-containing N-terminal region of CEP164 in tissue culture cells results in a dominant-negative effect on cilia formation, probably by competing with TTBK2 association with CEP164 at distal

 © 2021 The Author(s). Published by Elsevier Ltd.
This is an open access article under the CC BY license (http://creativecommons.org/licenses/by/4.0/).

CellPress
OPEN ACCESS

appendages (Cajanek and Nigg, 2014; Chaki et al., 2012; Slaats et al., 2014; Schmidt et al., 2012). Importantly, a chimera between CEP164, lacking its TTBK2-interacting N-terminal region, and a kinase domain containing part of TTBK2 is sufficient to rescue the ciliogenesis defect in CEP164-depleted cells (Cajanek and Nigg, 2014). Together, these data argue that recruitment of TTBK2 to the distal appendages by CEP164 is the main function of CEP164's N-terminal region and that this step is critical for the initiation of ciliogenesis.

Despite the strong evidence that functionally links CEP164 and TTBK2, many questions remain open concerning this complex. Most of the functional insights rest on the use of truncation constructs, and biochemical analyses on this complex, including its binding affinity, are largely missing. Importantly, despite the crucial importance for ciliogenesis of TTBK2 recruitment to CEP164, the mechanisms by which this step is regulated are currently largely unknown. Phosphatidylinositol-4-phosphate binding to TTBK2 and CEP164 has been suggested to compromise TTBK2-CEP164 complex formation (Xu et al., 2016), but the structural basis of this proposed mechanism is unclear and other regulatory mechanisms might act in parallel. Directly in the vicinity of the proposed CEP164-binding motif in TTBK2 is located an EB1/3 binding motif important for TTBK2 recruitment to microtubules (Oda et al., 2014; Watanabe et al., 2015; Jiang et al., 2012). Thus, EB1/3 and CEP164 binding to TTBK2 might be sterically mutually exclusive, a mechanism that could contribute to the regulation of TTBK2 recruitment. Other cellular factors might directly compete with TTBK2 binding to CEP164 by mimicking its WW domain-binding motif. The identification of TTBK2's critical CEP164-binding motif might therefore facilitate the identification of putative TTBK2 regulating factors.

There are currently no high-resolution structural data available that could shed light on the molecular basis of CEP164-TTBK2 complex formation. The lack of structural information hampers our ability to derive the binding motif for the interaction with the WW domain of CEP164 and prevents insight into the structural constraints and plausibility of the proposed regulatory mechanism of TTBK2 recruitment. Importantly, high-resolution structural information on the CEP164-TTBK2 complex is also crucial to understand its involvement in human disease. Mutations in centriole components such as CEP164 that impair cilia formation or function are either incompatible with human life or result in disorders referred to as ciliopathies (Fliegauf et al., 2008; Mitchison and Valente, 2017; Braun and Hildebrandt, 2017). Nephronophthisis is an autosomal recessive ciliopathy that constitutes the most frequent genetic cause of kidney failure in children (Hildebrandt et al., 2009). Probably due to compromised signaling pathways that affect planar cell polarity, cysts and fibrosis progressively arise in the kidney, leading to kidney failure (Braun and Hildebrandt, 2017; Fischer et al., 2006; Hildebrandt et al., 2009; Stokman et al., 2021). To date, there is no treatment available to slow the progressive loss of kidney function, and patients ultimately require dialysis or kidney transplantation to survive (Hildebrandt et al., 2009; Stokman et al., 2021).

A structural understanding of how ciliopathy mutations affect CEP164 function might therefore inform translational research approaches aimed at alleviating disease progression in patients. The two missense mutations Q11P (homozygous) and R93W (compound heterozygous with a Q525X truncation mutation) in CEP164 are associated with nephronophthisis, while a homozygous CEP164 R93W mutation also has been identified in a patient with BBS (Bardet-Biedl syndrome)-like syndrome (Chaki et al., 2012; Maria et al., 2016). It is currently not known whether and how these mutations affect the interaction between CEP164 and TTBK2 to give rise to the underlying ciliopathy. Furthermore, it is unclear what potential strategies could be used to address CEP164 dysfunction in these ciliopathies. While both mutations fall within the N-terminal region of CEP164, its WW domain is located between residues 56 and 89 (Cajanek and Nigg, 2014) and therefore is not directly targeted by the Q11P and the R93W mutations.

In this paper, we present a detailed biochemical, functional, and high-resolution structural analysis of the complex between the N-terminal domain (NTD) of CEP164 and its TTBK2-binding motif. Our data unambiguously demonstrate that the Q11P and R93W ciliopathic mutations compromise complex formation, reveal the structural basis of the underlying processes, and suggest possible approaches to mitigate their deleterious effects. Furthermore, our data and analyses provide insight into how TTBK2 recruitment to CEP164 affects TTBK2 activities and also allow us to propose a model of how TTBK2 is able to reach its substrates while bound at the distal appendages. Together, our data provide high-resolution structural information on a distal appendage complex and deepen our understanding of a critical step in cilia formation in human health and disease.

RESULTS

Unraveling the TTBK2-CEP164 interaction

TTBK2 interacts through a proline-rich C-terminal region ($TTBK2^{1074-1085}$) with a predicted WW domain ($CEP164^{62-84}$) in the N-terminal part of CEP164 (Cajanek and Nigg, 2014; Oda et al., 2014). Consistent with these findings, we established that the ability of FLAG-tagged CEP164 to pull down hemagglutinin (HA)-tagged TTBK2 depended on both elements (Figures 1A and 1B). Furthermore, pull-down experiments with recombinantly produced GST-$CEP164^{1-109}$ and cell extracts from tissue culture cells expressing $TTBK2^{1074-1087}$-GFP also established that $CEP164^{1-109}$ and $TTBK2^{1074-1087}$ are sufficient to bind to each other (Figures S1A and S1F). Similar experiments, but with $TTBK2^{1074-1087}$ constructs carrying alanine mutations in consecutive blocks of four amino acids, suggested that the residues within $TTBK2^{1074-1083}$ (the first three mutated blocks) contribute strongly to CEP164 binding, while the effect of those residues within $TTBK2^{1084-1087}$ (the last mutated block) is limited (Figure S1A).

To determine the corresponding binding affinity of the TTBK2-CEP164 complex, we performed isothermal titration calorimetry (ITC) with purified recombinant $CEP164^{1-109}$ (Figure S1H) and synthetically produced $TTBK2^{1074-1087}$ (Figure 1C; Table 1). Analysis of the ITC data suggested a binding affinity of ~60 μM, which is in agreement with the binding affinities observed for other WW domain-proline-rich binding motif (PRBM) interactions (Iglesias-Bexiga et al., 2015; McDonald et al., 2011; Verma et al., 2018).

Structure and dynamics of the NTD of CEP164

To gain insight into the structural basis of TTBK2 binding by CEP164, we first determined the high-resolution structure of

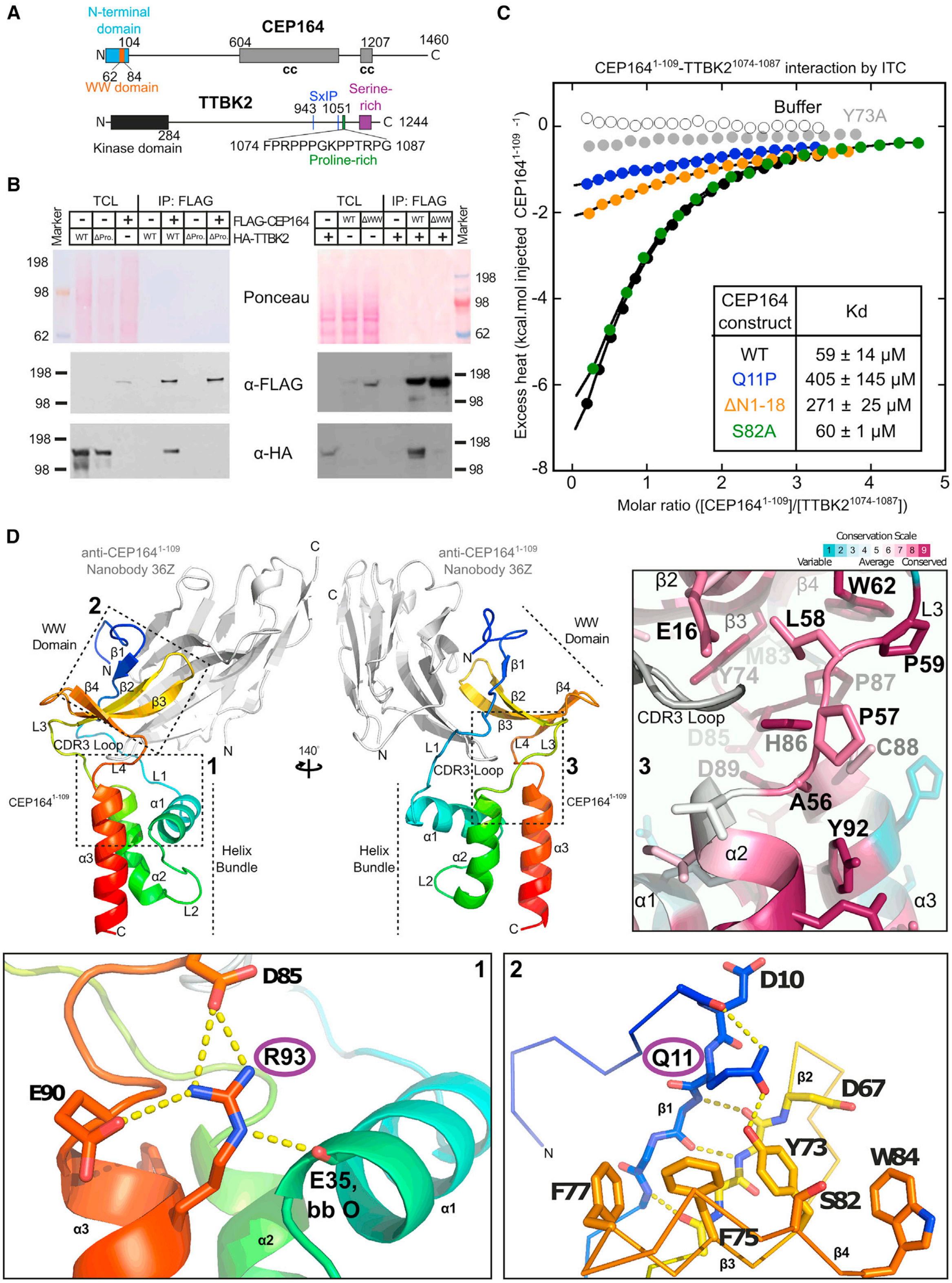

Figure 1. High-resolution structure of CEP164$^{1–109}$ that interacts with TTBK2$^{1074–1087}$ with low micromolar affinity

(A) Domain organization of human CEP164 and TTBK2. cc, coiled coil. Amino acid residue numbers are indicated.

(B) The WW domain of CEP164 and the proline-rich region 1,074–1,087 in TTBK2 are necessary for the CEP164-TTBK2 interaction. Western blots showing pull-down experiments with lysates from cells expressing 3xFLAG-tagged CEP164 and 3xHA-tagged TTBK2 constructs. Left: pull-down with 3xFLAG-CEP164 and

(legend continued on next page)

CellPress
OPEN ACCESS

the NTD of CEP164 ($CEP164^{1-109}$) from two different crystal forms by X-ray crystallography to a resolution of 1.6 and 1.7 Å (Table 2; Figures 1D and S2). These crystal forms were obtained by co-crystallization with two different camelid nanobodies (10Z and 36Z) that were raised against $CEP164^{1-109}$.

The $CEP164^{1-109}$ (CEP164-NTD) structure from the first crystal form (Figure 1D, co-crystallized with nanobody 36Z) revealed a globular shape composed of a head, neck, and body delineating the WW domain, connecting linkers and an α-helical domain. The WW domain has a canonical three-stranded topology (β2–4 in Figure 1D) and is inserted into the helical domain. The N-terminal region of CEP164-NTD preceding the helical domain is partly unstructured with the exception of residues 12–14 that form a short fourth strand (β1 in Figure 1D) stabilized by mainchain-mainchain hydrogen bonds to β2 of the WW domain. The β1-strand is also held in place by additional contacts, including a sidechain-mainchain hydrogen bond between R7 and D67, and a sidechain-sidechain hydrogen bond between Y73 and Q11 (Figure 1D; magnified view, panel 2, Figure S2B). Intriguingly, Q11 is mutated to proline in a CEP164 allele that is associated with the human ciliopathy nephronophthisis (Chaki et al., 2012).

As expected, the $CEP164^{1-109}$ structure derived from the second crystal form (obtained by co-crystallization with nanobody 10Z) is very similar to the first structure (root-mean-square deviation [RMSD]: 0.86Å over 87 aligned CEP164 residues), including the position of the bound nanobody (Figure S2). Surprisingly, the very N terminus, including the short additional β1-strand of the WW domain, is highly divergent in both structures. While such a strand is observed at a comparable place in the second $CEP164^{1-109}$ structure, it is formed by residues 4–6 (not residues 12–14) and is held in place only through mainchain interactions with β2 of CEP164. The structural promiscuity of this region might argue that the short β1-strand is only weakly bound. In agreement with this notion, NMR experiments with ^{15}N-labeled purified, recombinant $CEP164^{1-109}$ confirm the high flexibility of the very N-terminal region of CEP164 in solution (residues 1–23) (Figure S3A, (Berjanskii and Wishart, 2005)). Residues 7–14 show marginally higher $\{^1H\}^{15}N$ heteronuclear Overhauser effect (hetNOE) values (~0.5) than their flanking residues but a lower degree of order than residues in the folded helical and WW domains. hetNOE data for the ^{15}N-labeled ciliopathic Q11P mutant did not reveal an increased flexibility of this N-terminal region. We conclude that the first 23 residues of the CEP164-NTD appear to associate only weakly with the WW domain.

The body of the $CEP164^{1-109}$ structure comprises an α-helical bundle whose three helices pack together to form a highly conserved hydrophobic core (Figure S4). The WW domain packs against the α-helical bundle and is held in place by the interconnecting linkers L3 and L4 (Figure 1D; magnified view, panel 3). In both crystal structures, the bound nanobodies are partially sandwiched between the WW domain and the helical bundle, allowing the complementary determining region 3 (CDR3) loop of the nanobodies to interact with residues at the interface between helical bundle and WW domain (Figures S2 and 1D).

To gain insight into the dynamics of both parts of the CEP164-NTD in the absence of nanobodies, we also determined the $CEP164^{1-109}$ structure by NMR (Figures 2A and 2B; Table 3). The obtained NMR ensemble structures, superposed to the helical bundle or WW domain, demonstrated that both parts of CEP164-NTD were highly similar in the 20 NMR ensemble structures and were also highly similar to the crystal structure of the nanobody-bound CEP164-NTD (Figures 2A and 2B). However, the relative orientation between both parts showed some variability due to slight rotation movements in the interconnecting linkers L3+4. As a result, the angle between α-helical bundle and WW domain in the NMR ensemble structures (see Figure 2A for definition) varied in the approximate range 97°–117°. The corresponding angle in the crystal structure of nanobody-bound $CEP164^{1-109}$ was ~83° (Figure 2C). This decrease is due to the CDR3 loop of the nanobody, which makes several bridging interactions between helical bundle and WW domain that pull both together (Figure 2A). We conclude that $CEP164^{1-109}$ displays some inter-domain flexibility and that the overall $CEP164^{1-109}$ structure is largely unaffected by the bound nanobodies.

Stability of the CEP164-NTD

Sequence analysis of the CEP164 homologs demonstrated that sequence conservation of CEP164-NTD is mainly found in the interior of the α-helical bundle and the solvent-exposed side of the WW domain (Figure S4) as well as in linker L3+4, which connects both domains (Figure 1D, magnified view - panel 3). Two nearly invariant proline residues, P59 and P87, in this linker region make hydrophobic contacts with the highly conserved W62. In addition, the invariant H86 in the vicinity forms a hydrogen bond with the A56 mainchain. The high conservation of this linker region suggests that inter-domain packing might be functionally important (for example, in stabilizing the WW domain fold).

To test this hypothesis, we deleted either WW domain or helical bundle from $CEP164^{1-109}$ by recombinantly joining the loops connecting them. Subsequently, we purified these constructs and examined their fold and thermostability using circular

3xHA-TTBK2 or 3xHA-TTBK2 carrying a deletion of its proline-rich region 1,074–1,087 (ΔPro.). Right: pull-down with 3xFLAG-CEP164 or 3xFLAG-CEP164 carrying a deletion containing its WW domain (ΔWW) and 3xHA-TTBK2. TCL, total cell lysate.

(C) Recombinant $CEP164^{1-109}$ and $TTBK2^{1074-1087}$ interact with low micromolar affinity. Typical ITC of a synthetic $TTBK2^{1074-1087}$ peptide and recombinant human $CEP164^{1-109}$ constructs carrying the indicated mutations, at 25°C. The resulting dissociation constants (K_D) are indicated as an average (±SD) from three to five independent measurements.

(D) Top left: ribbon representation of the structure of $CEP164^{1-109}$, rainbow-colored from N- to C-terminus, in complex with a camelid nanobody (in gray). Rotation, as indicated. Consecutive alpha helices (α), beta sheets (β), and linkers (L) are labeled. Dotted boxes labeled from 1 to 3 indicate the regions shown magnified in the three panels below (1 and 2) or to the right (3). Bottom: detailed view of the $CEP164^{1-109}$ regions containing residues R93 (box 1) or Q11 (box 2). These residues are mutated to W or P, respectively, in ciliopathies. Yellow dotted lines indicate hydrogen bonds. Selected residues are shown as sticks and are labeled. bb, backbone. Top right, ribbon representation of the region indicated by box 3, colored according to ConSurf (Ashkenazy et al., 2016) conservation scores from variable (cyan) to conserved (burgundy). Key residues in the interface region between the WW domain and the helical bundle are shown as sticks and are labeled.

Table 1. ITC analysis of the interaction between CEP164$^{1–109}$ constructs and TTBK2$^{1074–1087}$

CEP164$^{1-109}$ construct	N (±SD)	K_D (μM, ±SD)	ΔH (kcal/mol, ±SD)	n
WT	0.7 ± 0.2	59 ± 14	−17 ± 7	5
Q11P	1 (fixed)	405 ± 145	−9 ± 4	3
S82A	0.7 ± <0.1	60 ± 1	−14 ± <1	3
ΔN1-18	1 (fixed)	271 ± 25	−10 ± 1	3

dichroism (CD) spectroscopy (Figures 2D and S1I). Our results showed that CEP164$^{1-109}$ has a mixed α/β fold and unfolds with a T_m of approximately 55°C. The helical domain alone (CEP164$^{19-62,80-109}$) had a CD spectrum consistent with a highly α-helical structure, as expected, and displayed a thermostability similar to CEP164$^{1-109}$, while the spectrum and the lack of a cooperative denaturation transition for the WW domain alone (CEP164$^{1-28,50-94}$) indicated that it had a largely unfolded conformation. Furthermore, a deletion of the helical bundle from full-length FLAG-tagged CEP164 largely abolished its ability to pull down HA-tagged TTBK2 from lysates of cells expressing these constructs (Figure 2E). Thus, our data suggest that the CEP164 WW domain requires the intramolecular interaction with the helical bundle to fold stably and function *in vivo*.

Structural basis for TTBK2 binding by the CEP164-NTD

To understand in more detail how CEP164 and TTBK2 interact, we determined the high-resolution structure of the CEP164-NTD in complex with TTBK2$^{1074-1087}$ by X-ray crystallography to a resolution of 2.4 Å (Table 2, Figures 3A and S1E). Due to the low micromolar affinity of this complex ($K_D \sim 60$ μM, Figure 1C), we fused TTBK2$^{1074-1087}$ recombinantly to the N terminus of CEP164$^{1-109}$ to obtain diffraction-quality protein crystals of this complex. For crystallization, we again utilized nanobody 36Z as a crystallization chaperone. In the structure, neither the WW domain nor the α-helical bundle of the CEP164-NTD revealed significant conformational changes upon binding to TTBK2$^{1074-1087}$ (apo- and TTBK2-bound structures superposed with an RMSD of 1.03 Å over 99 structurally equivalent residues) (Figure S3B). The main difference resided in the N-terminal region of CEP164$^{1-109}$ where residues 6–8 form a second short strand in addition to the β1-strand of residues 12–14.

WW domains are categorized into four groups that differ in their ligand-binding specificity. Class I and IV WW domains recognize PPxY and phosphorylated (S/T)P motifs by accommodating their Xaa-P residues into a single, so-called XP groove defined by two highly conserved aromatic residues. In contrast, class II and III WW domains utilize two XP grooves, XP and XP2, to bind to PPLP and PxxGMxPP motifs, respectively (Kato et al., 2004; Zarrinpar and Lim, 2000). The CEP164$^{1-109}$-TTBK2$^{1074-1087}$ complex revealed a strong similarity to class II/III WW domains (Figure S5). Like these, it contains two XP grooves with the aromatic residues CEP164 Y73 and W84 defining the XP, and F75 and F77 the XP2 groove. TTBK2$^{1074-1087}$ adopts a polyproline helix II conformation and packs with core prolines against both grooves: P1075, P1077, P1078, and P1082 stack against CEP164 F75, F77, Y73, and W84, respectively, with P1075 occupying the XP2 and P1077-P1078 the XP groove (Figures 3A and S5A). P1079 and G1080 allow the backbone between residues P1078 and P1082 to bend like an arch over W84 to sandwich it. An additional contact between TTBK2 and CEP164 is made by R1076, which forms a salt bridge with residue D10 of CEP164, probably facilitating P1075 docking into the XP2 groove. D10 positioning is enabled by Q11 (mutated to proline in nephronophthisis), which hydrogen bonds with residues Y73 and D10. Finally, CEP164 residue S82 hydrogen bonds to the mainchain of the TTBK2 peptide. Residues TTBK2$^{1085-1087}$ were not visible in our structure, arguing that they do not make significant contact with CEP164 (Figure S1A). NMR-based binding experiments with purified ^{15}N-labeled CEP164$^{1-109}$ and unlabeled TTBK2$^{1074-1087}$ revealed an excellent agreement with our crystallographic analyses of this complex (Figure S3C).

Determinants of TTBK2 recognition by CEP164-NTD and the impact of nephronophthisis mutation Q11P

To dissect the importance of these interactions, we performed pull-down assays with purified GST-CEP164$^{1-109}$ and cell extracts from cells expressing TTBK2$^{1074-1087}$-GFP constructs containing individual alanine mutations. The results shown in Figure 3C suggest that residues F1074, R1076, P1077, P1078, and G1080 are particularly important for CEP164 binding. We confirmed the importance of the core residues in the context of the full-length proteins by pull-down experiments with FLAG-tagged CEP164 and HA-tagged TTBK2 (wild-type [WT] and mutant) (Figure S1C). Our structural data (Figure 3A) rationalize these biochemical findings with the exception of the strong impact of the F1074A mutant. While F1074 is well placed to make hydrophobic contacts with CEP164 residues F75 and F77, its electron density was poorly defined, arguing against a tight binding of this residue *in crystallo*.

To establish the importance of the interface residues Q11, Y73, and S82 in the CEP164-NTD, we purified recombinant CEP164$^{1-109}$, the ciliopathy mutant Q11P, and the S82A and Y73A mutants (Figure S1H). To ascertain the role of the N-terminal, flexible (Figure S3A) region of CEP164$^{1-109}$, we also purified a corresponding deletion construct (CEP164$^{19-109}$, Δ1–18). CD spectra analyses of these constructs suggested that their fold is not significantly perturbed compared with the WT protein with only very minor changes evident in the Y73A mutant (Figure S1D). Next, we determined the binding affinity of these mutants to synthetically produced TTBK2$^{1074-1087}$ by ITC (Figure 1C). Analysis of the ITC data suggested that the S82A mutation does not affect TTBK2 binding affinity. In contrast, Q11P and CEP164-NTD Δ1–18 both showed a strong drop in binding strength to ∼400 μM and ∼270 μM, respectively, while the Y73A mutant did not show any significant binding activity. These results confirm the crucial role of Y73 and of the ciliopathic residue Q11 for the TTBK2 interaction. In contrast, the contribution of S82 to TTBK2 binding in solution appears negligible.

We extended the mutational analysis of CEP164 interface residues further by performing pull-down assays with recombinantly produced GST-CEP164$^{1-109}$ WT, Q11P, Y73A, F75A, F77A, S82A, W84A, and Δ1–18 mutants (Figure S1F) and cell extracts from cells expressing TTBK2$^{1074-1087}$-GFP. In agreement with our previous data, S82A bound to TTBK2$^{1074-1087}$ similar to the WT protein, while mutations Q11P, Y73A, F77A, W84A, and Δ1–18 resulted in a strongly reduced and F75A in a moderately

CellPress
OPEN ACCESS

Table 2. X-ray crystallography dataset analysis and refinement statistics

Protein complex	Nanobody 10Z-CEP164$^{1-109}$	Nanobody 36Z-CEP164$^{1-109}$	Nanobody 36Z-TTBK2$^{1074-1087}$-CEP164$^{1-109}$
PDB accession code	7O06	7O0S	7O3B
Beamline	I04 (Diamond)	I04 (Diamond)	I03 (Diamond)
Space group	$P12_11$	I121	$C222_1$
Wavelength (Å)	0.97942	0.97942	0.97625
Monomers in the asymmetric unit	2	1	3
Unit cell dimensions (Å, °)	a = 50.9, b = 70.7, c = 62.0, α = 90.00, β = 90.09, γ = 90.00	a = 38.3, b = 50.6, c = 119.9, α = 90.0, β = 99.0, γ = 90.00	a = 70.6, b = 127.7, c = 218.9, α = 90.0, β = 90.0, γ = 90.0
Resolution (Å)	62.0–1.6	59.2–1.7	61.8–2.4
Completeness (overall/inner/outer shell) (%)	99.4/99.9/98.9	100.0/99.7/100.0	98.7/95.7/99.4
Rmerge (overall/inner/outer shell)	0.097/0.059/1.463	0.146/0.105/1.658	0.091/0.035/0.705
Rpim (overall/inner/outer shell)	0.040/0.025/0.627	0.061/0.047/0.700	0.052/0.018/0.436
Mean I/σI (overall/inner/outer shell)	9.9/27.0/1.7	6.1/18.2/1.0	8.6/17.0/2.1
Multiplicity (overall/inner/outer shell)	6.7/6.1/6.3	6.6/5.9/6.5	3.8/3.7/3.8
Wilson B-factor (Å^2)	19.2	24.9	33.5
Number of reflections (used in refinement)	57,658 (57,454)	25,042 (24,957)	38,541 (38,506)
Number of atoms	3,796	1,909	5,678
Waters	275	150	104
Rwork/Rfree (% data used)	0.1804/0.2108 (5%)	0.2019/0.2411 (5%)	0.1894/0.2395 (5%)
RMSD from ideal values: bond length/angles (Å/°)	0.006/0.797	0.006/0.813	0.004/0.623
Mean B value (Å^2)	28.06	31.80	55.0
Overall correlation coefficient Fo-Fc/Fo-Fc free	0.963/0.956	0.959/0.936	0.933/0.911
Molprobity score	1.21	1.34	1.71
Clashscore, all atoms	2.73	3.48	4.50
Poor rotamers (%)	1.83	1.05	3.34
Ramachandran outliers (%)	0.00	0.00	0.00
Ramachandran favored (%)	98.34	97.22	97.54

reduced TTBK2 binding (Figure 3B). We further confirmed these findings in the context of full-length proteins in a pull-down experiment with HA-tagged TTBK2 and FLAG-tagged CEP164, WT, Y73A, S82A, W84, and Q11P mutants (Figure S1B) that corroborated a strong (Y73A and W84A) or a significant reduction (ciliopathy mutation Q11P) of the TTBK2-CEP164 interaction, while no binding defect was observed for the S82A mutant. The apparently stronger binding of the Q11P mutant in the full-length context is probably explained by the fact that both CEP164 and TTBK2 are capable of self-interacting (Bowie et al., 2018; Sivasubramaniam et al., 2008), thereby increasing their binding affinity due to avidity effects. Together, our data confirm the structural model of the CEP164-TTBK2 complex, define its critical interactions, and establish the nephronophthisis-associated mutation Q11P as directly compromising the complex interface and binding affinity.

CEP164-NTD destabilization by nephronophthisis mutant R93W

In contrast to Q11P, the other CEP164-NTD-linked nephronophthisis-associated mutation R93W is not located near the TTBK2-CEP164 interface. Instead, R93 is located in the helical bundle, where it forms several hydrogen bonds predicted to stabilize it (Figure 1D; magnified view, panel 1). Thus, the R93W mutation might destabilize the helical bundle that is required for the TTBK2-interacting WW domain to fold stably (Figures 2D and 2E). To test whether this mutation indeed affects the CEP164-NTD stability, we purified the CEP164$^{1-109}$ R93W mutant and assessed its fold and thermostability by CD spectroscopy (Figures 3D and 3E). As a control, we also performed this experiment with the Q11P nephronophthisis mutant that structurally is not predicted to affect CEP164$^{1-109}$ stability (Figure 1D). Our results (Figures 3D and 3E) show that the R93W but not the Q11P mutation indeed strongly lowered the thermostability of the CEP164-NTD. Signs of destabilization were already evident around room temperature, while the mutant structure appeared largely unaffected at 7°C. Transverse relaxation optimized spectroscopy (TROSY) NMR experiments with ^{15}N-labeled CEP164$^{1-109}$ WT and R93W mutant corroborate the temperature-dependent structural instability of the R93W mutant but also indicate that the mutant structure is already compromised at 4°C (Figure S6A). We conclude that the ciliopathic R93W mutation destabilizes the CEP164-NTD fold.

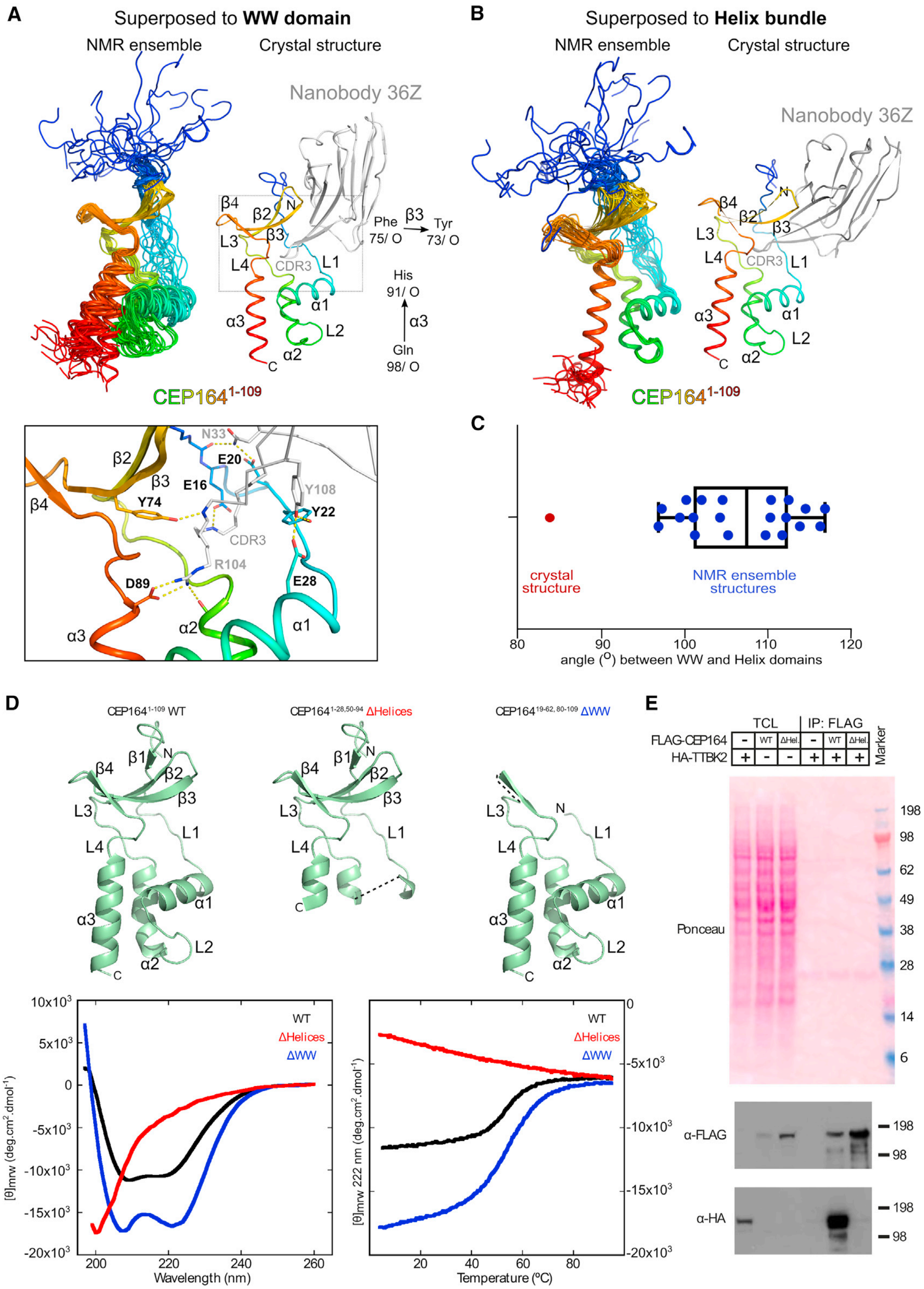

Figure 2. The isolated helical bundle but not the WW domain of CEP164$^{1-109}$ folds stably in solution

(A) Left, NMR structure ensemble of CEP164$^{1-109}$ superposed to its WW domain (CEP164$^{62-83}$), rainbow-colored from N (blue) to C terminus (red). Right, corresponding view of the superposed crystal structure of nanobody-bound CEP164$^{1-109}$ (nanobody in gray). The corresponding RMSD values (±SD) were 0.7 ± 0.2 Å and 0.9 ± 0.1 Å, respectively. Consecutive alpha-helices (α), beta-sheets (β), and linkers (L) are labeled. The vectors used to calculate the helical bundle-WW domain orientation angles (in C) are indicated. The dotted box designates the region shown magnified below. Bottom, detailed view of the CEP164$^{1-109}$ region contacted by the nanobody CDR3 loop.

(legend continued on next page)

CellPress
OPEN ACCESS

To establish the impact of the R93W mutation on TTBK2 binding, we employed pull-down assays with recombinantly produced GST-CEP164$^{1-109}$ WT, Q11P, and R93W (Figure S1G) and cell extracts from cells expressing TTBK2$^{1074-1087}$-GFP (Figure 3F). As seen before, the CEP164 Q11P mutation strongly diminished TTBK2 binding (to 7% of the WT level). In contrast, the impact of the R93W mutant was weaker but still significant, with the amount of pulled down TTBK2$^{1074-1087}$-GFP reduced to 60% of the WT level. This experiment had to be performed at 4°C, a temperature at which the impact of the R93W mutation on the stability of the NTD of CEP164 is small (Figure 3D). We also performed ITC experiments with the TTBK2$^{1074-1087}$ peptide and the CEP164$^{1-109}$ R93W mutant at 25°C, and these experiments indicated a moderate reduction in binding affinity compared with the WT. However, the ITC data were complicated by the coupling of peptide binding and refolding of a fraction of the CEP164$^{1-109}$ R93W mutant, since the CEP164 construct is destabilized and partially unfolded at 25°C (Figures 3D and S6A), so we are unable to assign a more precise value to the effect on binding. Together, our data suggest that the ciliopathic R93W mutation negatively affects the CEP164-TTBK2 interaction through CEP164-NTD destabilization.

CEP164 Q11P and R93W mutants affect cilia formation in RPE-1 cells

Since the ciliopathy-associated Q11P and R93W CEP164 mutants showed reduced binding to TTBK2 in our pull-down assays, we asked whether they would also have an impact on ciliogenesis in rescue experiments with human, serum-starved CEP164-null telomerase reverse transcriptase-immortalized retina pigmentation epithelial (hTERT RPE-1) cells (Daly et al., 2016). Both CEP164 mutations did not interfere with CEP164 localization to the mother centriole but showed a significant reduction in ARL13b-positive cilia formation compared with the WT construct (Figures 3G and 3H). Consistent with our biochemical data, the CEP164 R93W mutation resulted in a less strong effect than the Q11P mutation in this assay (Figure 3H). Thus, our data confirm the genetic data in patients (Chaki et al., 2012; Maria et al., 2016) and suggest that two CEP164 mutations that give rise to ciliopathies with comparable strength in patients show differential impacts on ciliogenesis in tissue culture cells.

CEP164-NTD binding inhibits EB1 engagement by TTBK2

TTBK2 is not only recruited to centriole distal appendages but also to microtubule ends through a SxIP motif-mediated interaction with the end binding protein EB1/3 (Watanabe et al., 2015; Jiang et al., 2012). Of the two SxIP motifs present in TTBK2, the second motif (TTBK2$^{1051-1054}$) contributes the most strongly to EB1/3 binding (Watanabe et al., 2015) and is located in the vicinity of the CEP164-interacting motif of TTBK2 (TTBK2$^{1074-1084}$). To check whether EB1 and CEP164-NTD can engage this part of TTBK2 simultaneously, we performed pull-down experiments with purified MBP-TTBK2$^{1033-1087}$ (WT, TTBK2$^{1051-1054\ \mathrm{SKIP\ to\ AAAA}}$, or TTBK2$^{1076-1078\ \mathrm{RPP\ to\ AAA}}$) and EB1 in the presence of an excess of CEP164$^{1-109}$. The results shown in Figure 4A demonstrate that the presence of CEP164-NTD strongly decreased EB1 binding to TTBK2$^{1033-1087}$ compared with the TTBK2$^{1076-1078\ \mathrm{RPP\ to\ AAA}}$ mutant that is unable to engage CEP164-NTD. In contrast, excess EB1-GFP does not efficiently compete with CEP164-NTD binding to MBP-TTBK2$^{1033-1087}$ (Figure S7B), probably due to its lower binding affinity. Further pull-down experiments with 3xFLAG-EB1 and full-length 3xHA-TTBK2 (WT or CEP164-binding-deficient ΔPro-rich [Δ1,074–1,087] mutant) in the presence of GST-CEP164$^{1-109}$ (WT or TTBK-binding deficient Y73A mutant) confirm our findings (Figure S7A). We conclude that CEP164-NTD binding inhibits EB1 engagement of the major EB1 binding site in TTBK2.

CEP164-NTD binding does not stimulate TTBK2 autophosphorylation

Many kinases exist in an auto-inhibited state that is relieved by an activating event such as phosphorylation or protein binding. Thus, we wanted to test whether CEP164-NTD binding to TTBK2 activates its kinase activity. To this end, we recombinantly produced dephosphorylated N-terminally 3xFLAG- and C-terminally STREP-tagged TTBK2 kinase (WT and kinase dead D163A mutant). The WT but not the D163A mutant TTBK2 kinase efficiently phosphorylated itself, as judged by its strong upward mobility in SDS-PAGE gels after incubation with ATP. Incubation with an excess of purified CEP164$^{1-109}$, WT, or TTBK2-binding-deficient Y73A mutant did not stimulate TTBK2 phosphorylation levels compared with the corresponding buffer control (Figure S7C). Thus, TTBK2 binding to CEP164 does not strongly activate TTBK2 kinase activity.

CEP164 and TTBK2 contain long regions predicted to be unstructured

Short binding motifs are often found in extended, open conformation, such as regions of disorder. To evaluate TTBK2 for the presence of such regions, we analyzed its sequence using several different disorder prediction algorithms (MFDp2, Mizianty et al., 2013; SPOT-Disorder, Hanson et al., 2017, 2019; or

(B) Similar to (A), but CEP164$^{1-109}$ NMR structures superposed to their helical bundle (CEP164$^{26-55,\ 87-103}$). The corresponding RMSD values (±SD) were 0.5 ± 0.1 Å and 0.8 ± 0.1 Å, respectively.
(C) Measured angles between the helical bundle and the WW domain in the NMR and crystal structures as indicated in (A). The average value across the NMR ensemble was 107° ± 7°. The individual angle values in each ensemble member are overlaid to a box-and-whisker plot. The whiskers represent the maximum and the minimum measured values, while the box ranges between the first and the third quartiles; the median value is also indicated.
(D) Top: ribbon representation of the CEP164$^{1-109}$ structure (in green). The ΔHelices and ΔWW panels indicate the construct deletions of the helical bundle or WW domain, respectively, by omitting the corresponding structural regions. Dotted black lines highlight the parts that were directly spliced together in the constructs. Alpha helices (α), beta sheets (β), and linkers (L) are labeled. Bottom: CD analyses of recombinant CEP164$^{1-109}$ WT as well as the corresponding ΔHelices and ΔWW constructs. Left: buffer-subtracted CD spectra at 25°C. Right: CD-based thermal melting analysis recorded at 222 nm.
(E) The helical bundle of CEP164 is necessary for the CEP164-TTBK2 interaction. Western blot showing a pull-down experiment with lysates from cells expressing 3xHA-TTBK2 and 3xFLAG tagged CEP164 or 3xFLAG-CEP164 carrying a deletion of its helical bundle. ΔHel., construct as indicated by ΔHelices in (D). TCL, total cell lysate.

Table 3. Structural statistics for the NMR solution structure of CEP164$^{1–109}$

**Structural restraints**			
NOE-derived distance restraints			
intraresidue	668	very strong (0–2.5 Å)	27
sequential	483	strong (0–3.0 Å)	36
medium (2 ≤ | i-j | ≤ 4)	339	medium (0–4.0 Å)	118
long (| i-j | > 4)	365	weak (0–5.5 Å)	1674
total	1855	total	1855
Dihedral restraints			
φ	81		
ψ	82		
ω	113		
H-bond restraints			
distances	72		
H bonds	36		
**Statistics for accepted structures**			
Number of accepted structures	20		
Mean AMBER energy terms (kcal/mol ±SD)			
E (total)	−5,431.4 ± 21.2		
E (van der Waals)	−797.8 ± 12.9		
E (distance restraints)	15.9 ± 3.6		
Distance restraint violations >0.2 Å (average number per structure)	4.7 ± 2.1		
Angle restraint violations >5° (average number per structure)	12.1 ± 3.6		
**RMSDs from the ideal geometry used within AMBER**			
bond lengths	0.0102 Å		
bond angles	2.11°		
Ramachandran statistics			
most favored	94.2%		
additionally allowed	5.4%		
generously allowed	0.2%		
disallowed	0.2%		
Average atomic RMSDs from average structure (±SD) residues 26–36, 44–53, 90–103			
N, Cα, C backbone atoms	0.27 ± 0.06 Å		
all heavy atoms	0.91 ± 0.07 Å		

AUCpreD, Wang et al., 2016) that have ranked highly in a recent experimental benchmarking (Nielsen and Mulder, 2019). The results shown in Figure 4B suggest that, outside its N-terminal kinase domain, TTBK2 contains large regions of disorder. Thus, TTBK2 probably has a high level of flexibility between its kinase domain and its C-terminal CEP164-binding region. This flexibility might facilitate its access to its known substrates (Bernatik et al., 2020; Huang et al., 2018; Lo et al., 2019) at or around the centriole wall.

DISCUSSION

Ciliopathies are genetic diseases that arise when mutations impair cilia formation or function. Elucidation of the corresponding molecular mechanisms can lead to a deepened understanding of the underlying biological processes and is an important first step toward the development of targeted therapeutic strategies. The recruitment of TTBK2 by the centriole distal appendage component CEP164 is one of the crucial early events in cilia formation (Cajanek and Nigg, 2014; Oda et al., 2014; Huang et al., 2018).

The structures of CEP164-NTD revealed a two-domain organization in which a WW domain is inserted into an α-helical bundle consisting of three α helices. Despite an element of inter-domain flexibility, these two domains act as a single unit whose stability critically depends on the core interactions within the helical bundle. CEP164-NTD accommodates the TTBK2 proline-rich peptide into two non-continuous hydrophobic grooves (XP and

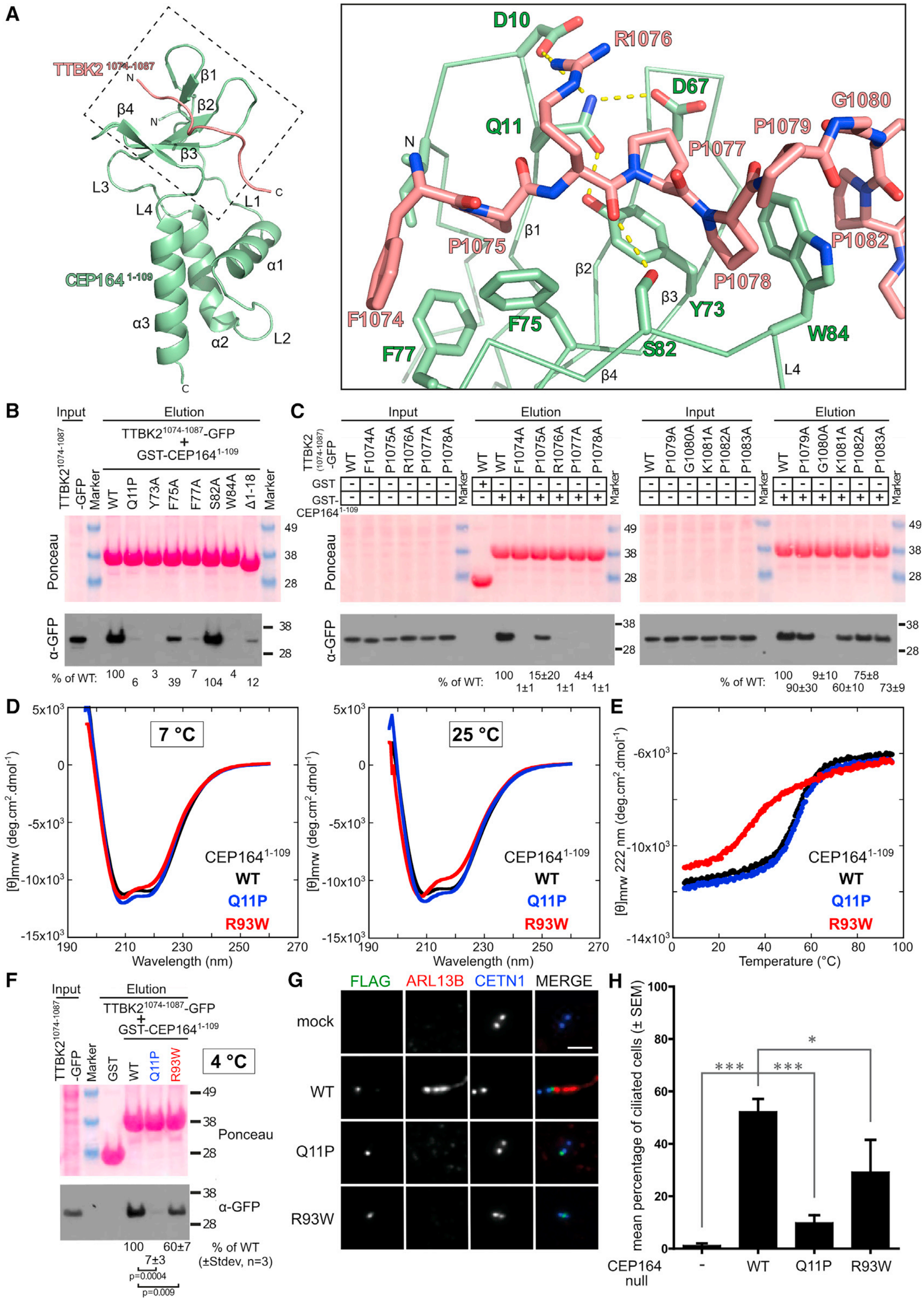

Figure 3. High-resolution structure of the CEP164$^{1–109}$-TTBK2$^{1074–1087}$ complex that is compromised by the ciliopathy-associated mutations Q11P and R93W in CEP164

(A) Left: ribbon representation of the structure of CEP164$^{1-109}$ (green) in complex with TTBK2$^{1074-1087}$ (red). The structure of the bound camelid nanobody used for co-crystallization is omitted for optical clarity. Consecutive alpha-helices (α), beta-sheets (β), and linkers (L) are labeled. The dotted box is shown magnified to the right. Right: detailed view of the interface region between CEP164$^{1-109}$ and TTBK2$^{1074-1087}$. Key interface residues are shown as sticks and are labeled. Yellow dotted lines indicate hydrogen bonds.

(legend continued on next page)

XP2). While both ligand-binding interface and ligand orientation in the complex structure showed striking similarities to class II/III WW domain complexes (Kato et al., 2004), there are also features unique to the CEP164-NTD–TTBK2 interaction. In CEP164-NTD, the XP2 groove is formed by two phenylalanines (F75, F77) and a tyrosine (Y73) instead of a tryptophan and tyrosine or two tyrosines as observed in FE65 and FBP11 WW domains, respectively. Furthermore, the CEP164-NTD contains a flexible N-terminal region that complements the WW domain in the complex structure with an additional two-stranded β-hairpin that directly contributes to ligand binding. The side-chains of I8 and V13 in this region contribute to the shape of the XP2 groove. As a result, this groove is considerably smaller and deeper than the grooves of other class II/III WW domains and accommodates a single proline residue instead of a pair of prolines. This difference might also explain the importance of F1074 in providing additional hydrophobic contacts that contribute to complex stability.

The mutations Q11P and R93W in CEP164 are associated with ciliopathies (Chaki et al., 2012; Maria et al., 2016; Slaats et al., 2014). Q11P was identified as a homozygous mutation and R93W as a compound heterozygous mutation with the Q525X truncation in nephronophthisis patients that displayed early kidney failure and retinal degeneration (Chaki et al., 2012). The Q525X mutation probably results in a strongly dysfunctional CEP164 truncation, as it is unable to localize to the mother centriole. Consistent with this notion, patients with the similar but homozygous truncation mutation R576X show more severe phenotypes with additional features typical for motile ciliopathies, such as primary ciliary dyskinesia (Chaki et al., 2012). A homozygous R93W CEP164 mutation was also found in a patient with BBS (Bardet-Biedl syndrome)-like syndrome with hypogonadism, retinal phenotypes, and obesity but without renal anomalies (Maria et al., 2016).

Our data show that the CEP164 Q11P and R93W mutants localize normally to the mother centriole but are defective in rescuing cilia formation in human, CEP164-null hTERT RPE-1 cells, confirming the genetic data in human patients (Chaki et al., 2012; Maria et al., 2016). Although both mutations are located away from the WW domain of CEP164 (residues 62–84), they affect its ability to engage TTBK2 in pull-down assays. Structurally, the R93W mutation reduces the thermostability of the CEP164-NTD by impairing the folding of the WW domain-stabilizing helical bundle. In contrast, residue Q11 is part of a short β-strand that associates with the WW domain and contributes to the placement of several side chains that directly contact TTBK2. The Q11P mutation compromises these interactions. Intriguingly, the removal of the first 18 residues of CEP164-NTD has a less strong effect on TTBK2 binding than Q11P. Thus, we speculate that the Q11P proline partly occupies the XP grooves of CEP164-NTD and thereby also acts to compete with TTBK2 binding. Our data suggest that the corresponding CEP164-NTD region is flexible enough to allow these movements. In terms of therapeutic potential, our data suggest that reagents that stabilize the CEP164-NTD fold in the R93W mutant, or prevent the WW domain association of the Q11P region, would hold promise in alleviating disease manifestation in the corresponding patients.

Our *in vitro* and *in vivo* data suggest that the R93W mutation affects TTBK2 binding and cilia formation less strongly than the Q11P mutation. Nevertheless, in patients, no corresponding clear-cut genotype-phenotype relationship is obvious (Chaki et al., 2012; Maria et al., 2016). CEP164 might have functions outside ciliogenesis that contribute to disease manifestations to account for this observation. Thus, CEP164 was suggested to function in cell cycle progression and its dysfunction, for example in the R93W mutant, to contribute to the pathogenesis of nephronophthisis through DNA damage-induced replicative stress, although this mutant was also found to have some negative effect on cilia formation (Slaats et al., 2014). However, in another study, CEP164 was not found to influence cell proliferation or the DNA damage response (Daly et al., 2016). Thus, as suggested for two closely related ciliopathy mutations in CEP120 (Joseph et al., 2018), the genetic backgrounds of the affected patients might modify the exact clinical manifestation of these ciliopathy mutations instead.

Our data also give insights into how TTBK2 binding to CEP164 might influence other TTBK2 activities. Located close to the CEP164-NTD binding region of TTBK2 is one of two EB1-binding SxIP motifs. This motif is responsible for the majority of the EB1-binding affinity of TTBK2 (Watanabe et al., 2015, Figure S7A). EB1-dependent TTBK2 recruitment to microtubules is important for microtubule regulation (Watanabe et al., 2015), and we show here that CEP164-NTD binding inhibits EB1 engagement by

(B) Several interfacial residues of the CEP164 WW domain are critical for its interaction with TTBK2. Western blot showing a pull-down experiment with recombinant GST or GST-CEP164$^{1-109}$, WT or the indicated mutants, and lysates from cells expressing C-terminally GFP-tagged TTBK2$^{1074-1087}$. Below the blot, the band intensities of the eluates (as percentage of the WT level) are shown.

(C) Several residues within the CEP164-binding region of TTBK2 are essential for its interaction with CEP164 and define a CEP164-binding motif. Western blot showing a pull-down experiment with recombinant GST or GST-CEP164$^{1-109}$ and lysates from cells expressing C-terminally GFP-tagged TTBK2$^{1074-1087}$ constructs. TTBK2 constructs carried single alanine mutations as indicated above the blot. Below the blot, the band intensities of the eluates (as percentage of the WT level) are shown (±SD, n = 3).

(D and E) The R93W, but not the Q11P mutation decrease the thermostability of the NTD of CEP164. (D) Buffer-subtracted CD spectra of recombinant CEP164$^{1-109}$ WT as well as its Q11P and R93W mutants at 7°C and 25°C. (E) CD-based thermal melting analysis of CEP164$^{1-109}$ WT, Q11P, and R93W at 222 nm.

(F) Both Q11P and R93W nephronophthisis mutations in CEP164$^{1-109}$ compromise the interaction with TTBK2$^{1074-1087}$ at 4°C. Western blot showing a pull-down experiment with recombinant GST or GST-CEP164$^{1-109}$, WT, Q11P, or R93W and lysates from cells expressing C-terminally GFP-tagged TTBK2$^{1074-1087}$. Below the blot, the band intensities of the eluates (as percentage of the WT level) are shown (±SD, n = 3).

(G and H) The CEP164 Q11P and R93W ciliopathy mutants localize to mother centrioles but do not efficiently rescue cilia formation in CEP164-null cells. (G) Immunofluorescence staining of hTERT RPE-1 CEP164-null cells rescued with the indicated, transfected 3xFLAG-tagged CEP164 constructs (green). Primary cilia were detected by ARL13b staining (red). CETN1 was used to visualize centrosomes (blue). Scale bar: 5 μm. (H) Quantification of the percentage of ciliated cells (mean percentage of ciliated cells ±SEM). Statistical significance between the groups was analyzed by one-way ANOVA with a Holm-Sidac multiple comparison post hoc test; number of cells, N_{WT} = 66, N_{Q11P} = 58, N_{R93W} = 55, and $N_{untransfected}$ = 57; *p = 0.02, ***p ≤ 0.0005.

CellPress
OPEN ACCESS

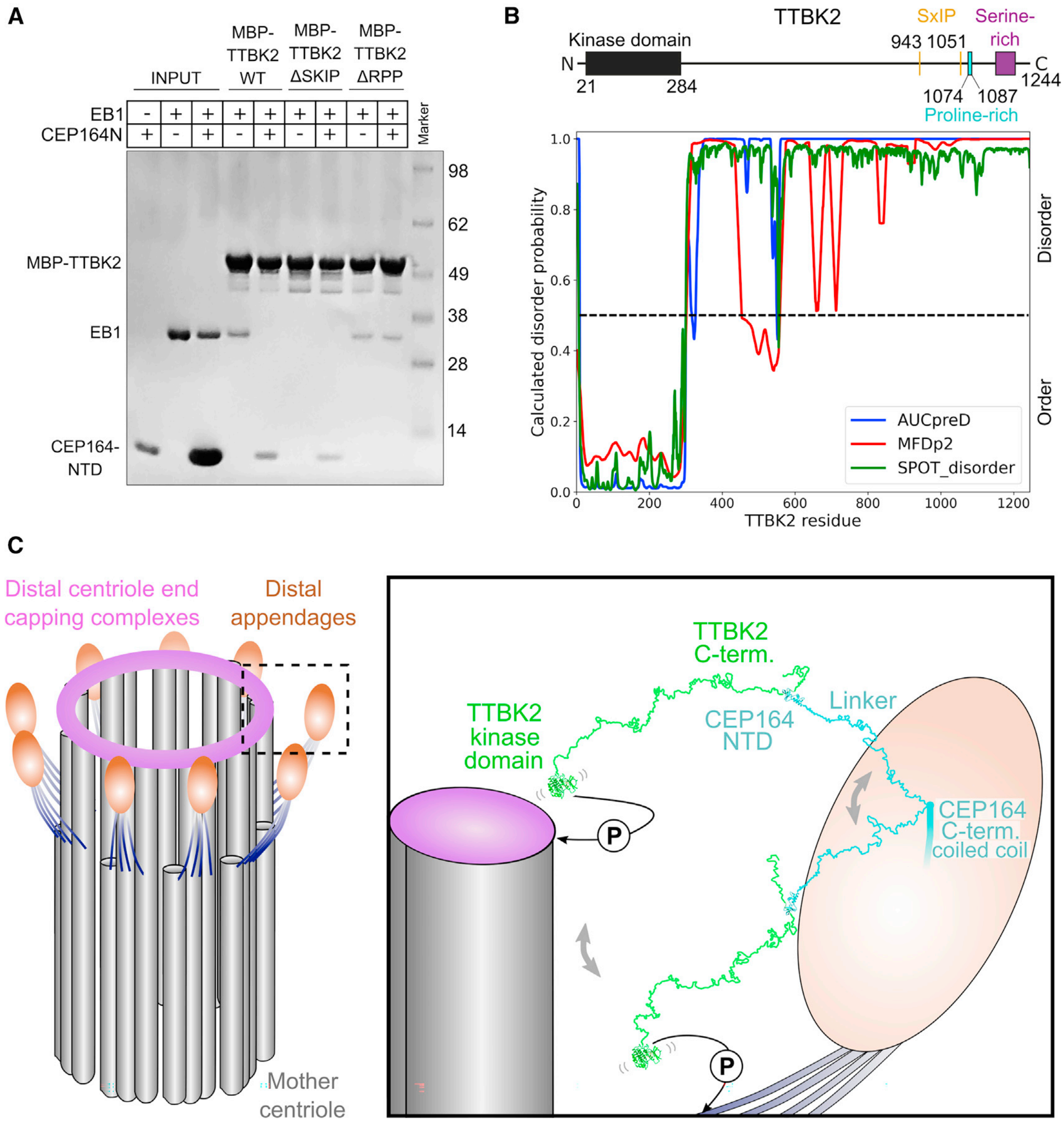

Figure 4. CEP164-NTD binding to TTBK2 inhibits the TTBK2-EB1 interaction

(A) CEP164$^{1-109}$ and EB1 compete for binding to MBP-TTBK2$^{1033-1087}$. Coomassie-stained SDS-PAGE gel showing pull-down experiments with purified MBP-TTBK2$^{1033-1087}$ (WT, EB1-binding-deficient TTBK2$^{1051-1054\ \text{SKIP to AAAA}}$ or CEP164-NTD-binding-deficient TTBK2$^{1076-1078\ \text{RPP to AAA}}$) with EB1 in the presence of an excess of CEP164$^{1-109}$.

(B) Per-residue disorder probabilities of TTBK2 as calculated by different disorder prediction algorithms. Values above 0.5 (dashed line) indicate disorder. The location of the kinase domain and selected sequence features of TTBK2 are indicated above the plot.

(C) Model of the TTBK2-CEP164 architecture at the distal appendages. Left: scheme of the mother centriole with distal appendages based on electron microscopy tomography segmentation analysis (Bowler et al., 2019). Subdistal appendages are omitted for clarity. Right: close-up view of a single distal appendage region. CEP164 is indicated in cyan, the bound TTBK2 is shown in green. The connecting linkers between CEP164-NTD and the CEP164 coiled-coil domain and between the CEP164-bound TTBK2$^{1074-1084}$ region and the N-terminal TTBK2 kinase domain (PDB: 6vrf) (Bao et al., 2021) are drawn in an extended and open conformation. Based on disorder predictions and the paucity of predicted secondary structure elements, we propose that these linkers are largely flexible (Figures 4B and S6B). This flexibility would allow the TTBK2-CEP164-NTD complex to sample larger areas of the distal appendage region and would enable TTBK2 to reach its phosphorylation substrates at (MPP9) or close to (CEP83) the distal centriole end. A circled "P" indicates a phosphorylation event by TTBK2.

TTBK2 *in vitro*. This argues that microtubule end association and CEP164 binding of TTBK2 might be mutually exclusive *in vivo* such that distal appendage-associated TTBK2 interacts with CEP164, while the non-centriolar TTBK2 fraction can interact with EB1/3 in the same cell. Preventing EB1/3 engagement and microtubule regulation by distal appendage-bound TTBK2 might be important for efficient cilia formation, but the functional importance of this putative mechanism in cells remains to be established. Intriguingly, the structural footprint of CEP164-NTD and EB1 on their TTBK2-binding regions (this study; PDB: 3gjo; Honnappa et al., 2009) is not large enough to directly account for the observed competition. Thus, CEP164-NTD and EB1 probably

engage larger regions of TTBK2 than their core binding motifs alone.

There are currently conflicting data concerning whether TTBK2 binding to CEP164 activates its kinase domain (Oda et al., 2014). To address this question, we performed TTBK2 phosphorylation assays with recombinantly produced TTBK2 (WT or kinase dead D163A mutant) in the presence of either binding-competent (WT) or -incompetent (Y73A mutant) CEP164-NTD. Our data did not reveal a stimulation of TTBK2 activity by CEP164-NTD binding. Thus, CEP164 might not be involved in activating an otherwise inactive TTBK2 but possibly recruits TTBK2 in an already activated form.

One of the puzzles concerning TTBK2 function after CEP164 binding is that its effector substrates CEP83 and MPP9 are located close to or at the centriole microtubule wall at a significant distance away from CEP164. While the N-terminal region of CEP164 *in vivo* is found at a radius of 121–284 nm from the centriole center (Bowler et al., 2019), CEP83 and MPP9 are found at a radius of 106–194 nm and ~113 nm, respectively (Bowler et al., 2019; Huang et al., 2018). Thus, TTBK2 would need to span a significant distance to reach these substrates.

While it has been suggested that TTBK2 might redistribute to a site closer to the centriole center (Lo et al., 2019), we favor an alternative explanation. Our bioinformatics analyses suggest that the TTBK2 region downstream of its N-terminal kinase domain (residues 21–284) and upstream of its CEP164-binding region (residues 1,074–1,084) contains large stretches of unfolded regions (Figure 4B). CEP164 also contains a region of ~500 residues downstream of its TTBK2-binding NTD and upstream of its first coiled-coil region, which appears largely unstructured (Figure S6B). Such regions, even with interspersed folded islands, can behave with random coil characteristics (Fitzkee and Rose, 2004). Our theoretical calculations suggest that a region of this combined length would display an average (root mean square) end-to-end distance of ~30 nm but could span much larger distances when extended. Thus, the kinase domain of (CEP164-bound) TTBK2 would probably be able to reach its substrates without relocation and CEP164-NTD, and its bound TTBK2 might be highly mobile around the distal appendages. In agreement with this notion, Bowler et al. (2019) recently showed that the N-terminal part of CEP164 displays a wider distribution at distal appendages that extends beyond their electron dense head structure. These findings accord well with our proposal that the CEP164 NTD is separated by a flexible linker from the appendage-anchored part of CEP164. We propose that the ability of distal appendage components to sample the wider area around them might be important for many different aspects of distal appendage function (Figure 4C).

STAR★METHODS

Detailed methods are provided in the online version of this paper and include the following:

- KEY RESOURCES TABLE
- RESOURCE AVAILABILITY
  - Lead contact
  - Materials availability
  - Data and code availability
- EXPERIMENTAL MODEL AND SUBJECT DETAILS
  - Cell lines
- METHOD DETAILS
  - Recombinant protein purification
  - Nanobody generation
  - Protein crystallization
  - X-ray crystallography data processing
  - Pull-down assays
  - Autophosphorylation assay
  - Bioinformatics
  - Circular dichroism (CD)
  - Isothermal titration calorimetry (ITC)
  - NMR
  - NMR structure calculations
  - Ciliogenesis rescue experiments
- QUANTIFICATION AND STATISTICAL ANALYSIS

SUPPLEMENTAL INFORMATION

Supplemental information can be found online at https://doi.org/10.1016/j.str.2021.08.007.

ACKNOWLEDGMENTS

We acknowledge M. Mazzorana (I04, MX15916-82) and N. Paterson (I03, MX21426-1) at the Diamond Light Source (UK), and M. Yu and J. O'Donnell (MRC-LMB) for dataset collection. We thank C. Morrison (NUI Galway), R. Williams, O. Perisic, and M. Hegde (MRC-LMB) for reagents. We thank M. van Roon (MRC-LMB) for technical support and helpful scientific discussions. We acknowledge the laboratory management staff from the School of Biological and Chemical Sciences and the Blizard Institute, Queen Mary University of London; the crystallization, X-ray crystallography, and NMR facilities from the MRC-LMB; and the CELLIM of CEITEC supported by the Czech-BioImaging large RI project (MEYS CR grant LM2018129). This work was supported by the Medical Research Council (file reference MC_UP_1201/3 to M.v.B. and a Cesar Milstein Fellowship for I.R.S.), the Queen Mary University of London (start-up grant to M.v.B.), the Czech Science Foundation (grant 19-05244S), and the Swiss National Science Foundation (grant IZ11Z0_166533 to L.C.).

AUTHOR CONTRIBUTIONS

I.R.S. and M.v.B. determined the crystal structures and performed the biochemical experiments. C.M.J. performed ITC. T.J.R. performed the NMR experiments. T.J.R. and D.N. solved the NMR structure. L.B. and L.C. performed the immunofluorescence experiments. A.A. contributed bioinformatics and structural analyses. M.v.B. supervised the project. I.R.S. and M.v.B. wrote the original draft of the manuscript. All authors contributed to the reviewing and editing of the manuscript. L.C. and M.v.B. secured funding.

DECLARATION OF INTERESTS

The authors declare no competing interests.

Received: April 30, 2021
Revised: July 19, 2021
Accepted: August 17, 2021
Published: September 8, 2021

REFERENCES

Afonine, P.V., Grosse-Kunstleve, R.W., Echols, N., Headd, J.J., Moriarty, N.W., Mustyakimov, M., Terwilliger, T.C., Urzhumtsev, A., Zwart, P.H., and Adams, P.D. (2012). Towards automated crystallographic structure refinement with phenix.refine. Acta Crystallogr. D Biol. Crystallogr. *68*, 352–367.

Aragón, E., Goerner, N., Zaromytidou, A.I., Xi, Q., Escobedo, AI., Massague, J., and Macias, M.J. (2011). A Smad action turnover switch operated by WW

CellPress
OPEN ACCESS

domain readers of a phosphoserine code. Genes Dev. *25*, 1275–1288. https://doi.org/10.1101/gad.2060811.

Argentaro, A., Yang, J.C., Chapman, L., Kowalczyk, M.S., Gibbons, R.J., Higgs, D.R., Neuhaus, D., and Rhodes, D. (2007). Structural consequences of disease-causing mutations in the ATRX-DNMT3-DNMT3L (ADD) domain of the chromatin-associated protein ATRX. Proc. Natl. Acad. Sci. U S A *104*, 11939–11944.

Ashkenazy, H., Abadi, S., Martz, E., Chay, O., Mayrose, I., Pupko, T., and Ben-Tal, N. (2016). ConSurf 2016: an improved methodology to estimate and visualize evolutionary conservation in macromolecules. Nucleic Acids Res. *44*, W344–W350.

Bao, C., Bajrami, B., Marcotte, D.J., Chodaparambil, J.V., Kerns, H.M., Henderson, J., Wei, R., Gao, B., and Dillon, G.M. (2021). Mechanisms of regulation and diverse activities of tau-tubulin kinase (TTBK) isoforms. Cell. Mol. Neurobiol. *41*, 669–685. https://doi.org/10.1007/s10571-020-00875-6.

Berjanskii, M.V., and Wishart, D.S. (2005). A simple method to predict protein flexibility using secondary chemical shifts. J. Am. Chem. Soc. *127*, 14970–14971. https://doi.org/10.1021/ja054842f.

Bernatik, O., Pejskova, P., Vyslouzil, D., Hanakova, K., Zdrahal, Z., and Cajanek, L. (2020). Phosphorylation of multiple proteins involved in ciliogenesis by Tau Tubulin kinase 2. Mol. Biol. Cell *31*, 1032–1046.

Bettencourt-Dias, M., Hildebrandt, F., Pellman, D., Woods, G., and Godinho, S.A. (2011). Centrosomes and cilia in human disease. Trends Genet. *27*, 307–315.

Bowie, E., and Goetz, S.C. (2020). TTBK2 and primary cilia are essential for the connectivity and survival of cerebellar Purkinje neurons. eLife *9*, e51166.

Bowie, E., Norris, R., Anderson, K.V., and Goetz, S.C. (2018). Spinocerebellar ataxia type 11-associated alleles of Ttbk2 dominantly interfere with ciliogenesis and cilium stability. PLoS Genet. *14*, e1007844.

Bowler, M., Kong, D., Sun, S.F., Nanjundappa, R., Evans, L., Farmer, V., Holland, A., Mahjoub, M.R., Sui, H.X., and Loncarek, J. (2019). High-resolution characterization of centriole distal appendage morphology and dynamics by correlative STORM and electron microscopy. Nat. Commun. *10*, 993.

Braun, D.A., and Hildebrandt, F. (2017). Ciliopathies. Cold Spring Harb. Perspect. Biol. *9*. https://doi.org/10.1101/cshperspect.a028191.

van Breugel, M., Wilcken, R., McLaughlin, S.H., Rutherford, T.J., and Johnson, C.M. (2014). Structure of the SAS-6 cartwheel hub from *Leishmania major*. eLife *3*, e01812.

Cajanek, L., and Nigg, E.A. (2014). Cep164 triggers ciliogenesis by recruiting Tau tubulin kinase 2 to the mother centriole. Proc. Natl. Acad. Sci. U S A *111*, E2841–E2850.

Case, D.A., Cheatham, T.E., Darden, T., Gohlke, H., Luo, R., Merz, K.M., Onufriev, A., Simmerling, C., Wang, B., and Woods, R.J. (2005). The Amber biomolecular simulation programs. J. Comput. Chem. *26*, 1668–1688.

Chaki, M., Airik, R., Ghosh, A.K., Giles, R.H., Chen, R., Slaats, G.G., Wang, H., Hurd, T.W., Zhou, W.B., Cluckey, A., et al. (2012). Exome capture reveals ZNF423 and CEP164 mutations, linking renal ciliopathies to DNA damage response signaling. Cell *150*, 533–548.

Chen, V.B., Arendall, W.B., III, Headd, J.J., Keedy, D.A., Immormino, R.M., Kapral, G.J., Murray, L.W., Richardson, J.S., and Richardson, D.C. (2010). MolProbity: all-atom structure validation for macromolecular crystallography. Acta Crystallogr. D Biol. Crystallogr. *66*, 12–21.

Chong, W.M., Wang, W.J., Lo, C.H., Chiu, T.Y., Chang, T.J., Liu, Y.P., Tanos, B., Mazo, G., Tsou, M.F.B., Jane, W.N., et al. (2020). Super-resolution microscopy reveals coupling between mammalian centriole subdistal appendages and distal appendages. eLife *9*, e53580.

Daly, O.M., Gaboriau, D., Karakaya, K., King, S., Dantas, T.J., Lalor, P., Dockery, P., Kramer, A., and Morrison, C.G. (2016). CEP164-null cells generated by genome editing show a ciliation defect with intact DNA repair capacity. J. Cell Sci. *129*, 1769–1774.

Diamond, R. (1995). COORDINATE-BASED CLUSTER-analysis. Acta Crystallogr. D Biol. Crystallogr. *51*, 127–135.

Emsley, P., and Cowtan, K. (2004). Coot: model-building tools for molecular graphics. Acta Crystallogr. D Struct. Biol. *60*, 2126–2132.

Evans, P.R., and Murshudov, G.N. (2013). How good are my data and what is the resolution? Acta Crystallogr. D Biol. Crystallogr. *69*, 1204–1214.

Fischer, E., Legue, E., Doyen, A., Nato, F., Nicolas, J.F., Torres, V., Yaniv, M., and Pontoglio, M. (2006). Defective planar cell polarity in polycystic kidney disease. Nat. Genet. *38*, 21–23.

Fitzkee, N.C., and Rose, G.D. (2004). Reassessing random-coil statistics in unfolded proteins. Proc. Natl. Acad. Sci. U S A *101*, 12497–12502.

Fliegauf, M., Benzing, T., and Omran, H. (2008). When cilia go bad: cilia defects and ciliopathies (vol 8, pg 880, 2007). Nat. Rev. Mol. Cell Biol. *9*, 88.

Goetz, S.C., Liem, K.F., and Anderson, K.V. (2012). The spinocerebellar ataxia-associated gene tau tubulin kinase 2 controls the initiation of ciliogenesis. Cell *151*, 847–858.

Graser, S., Stierhof, Y.D., Lavoie, S.B., Gassner, O.S., Lamla, S., Le Clech, M., and Nigg, E.A. (2007). Cep164, a novel centriole appendage protein required for primary cilium formation. J. Cell Biol. *179*, 321–330.

Hanson, J., Yang, Y., Paliwal, K., and Zhou, Y. (2017). Improving protein disorder prediction by deep bidirectional long short-term memory recurrent neural networks. Bioinformatics *33*, 685–692.

Hanson, J., Paliwal, K.K., Litfin, T., and Zhou, Y.Q. (2019). SPOT-Disorder2: improved protein intrinsic disorder prediction by ensembled deep learning. Genomics Proteomics Bioinformatics *17*, 645–656.

Hildebrandt, F., Attanasio, M., and Otto, E. (2009). Nephronophthisis: disease mechanisms of a ciliopathy. J. Am. Soc. Nephrol. *20*, 23–35.

Hommel, U., Harvey, T.S., Driscoll, P.C., and Campbell, I.D. (1992). Human epidermal growth-factor - high-resolution solution structure and comparison with human transforming growth factor-alpha. J. Mol. Biol. *227*, 271–282.

Honnappa, S., Gouveia, S.M., Weisbrich, A., Damberger, F.F., Bhavesh, N.S., Jawhari, H., Grigoriev, I., van Rijssel, F.J., Buey, R.M., Lawera, A., et al. (2009). An EB1-binding motif acts as a microtubule tip localization signal. Cell *138*, 366–376. https://doi.org/10.1016/j.cell.2009.04.065.

Huang, N., Zhang, D.H., Li, F.Y., Chai, P.Y., Wang, S., Teng, J.L., and Chen, J.G. (2018). M-Phase Phosphoprotein 9 regulates ciliogenesis by modulating CP110-CEP97 complex localization at the mother centriole. Nat. Commun. *9*, 4511.

Humbert, M.C., Weihbrecht, K., Searby, C.C., Li, Y.L., Pope, R.M., Sheffield, V.C., and Seo, S. (2012). ARL13B, PDE6D, and CEP164 form a functional network for INPP5E ciliary targeting. Proc. Natl. Acad. Sci. U S A *109*, 19691–19696.

Iglesias-Bexiga, M., Castillo, F., Cobos, E.S., Oka, T., Sudol, M., and Luque, I. (2015). WW domains of the yes-kinase-associated protein (YAP) transcriptional regulator behave as independent units with different binding preferences for PPxY motif-containing ligands. PLoS One *10*. https://doi.org/10.1371/journal.pone.0113828.

Ishikawa, H., and Marshall, W.F. (2011). Ciliogenesis: building the cell's antenna. Nat. Rev. Mol. Cell Biol. *12*, 222–234.

Jiang, K., Toedt, G., Montenegro Gouveia, S., Davey, N.E., Hua, S., van der Vaart, B., Grigoriev, I., Larsen, J., Pedersen, L.B., Bezstarosti, K., et al. (2012). A proteome-wide screen for mammalian SxIP motif-containing microtubule plus-end tracking proteins. Curr. Biol. *22*, 1800–1807.

Joseph, N., Al-Jassar, C., Johnson, C.M., Andreeva, A., Barnabas, D.D., Freund, S.M.V., Gergely, F., and van Breugel, M. (2018). Disease-associated mutations in CEP120 destabilize the protein and impair ciliogenesis. Cell Rep. *23*, 2805–2818.

Joukov, V., and De Nicolo, A. (2019). The centrosome and the primary cilium: the yin and yang of a hybrid organelle. Cells *8*. https://doi.org/10.3390/cells8070701.

Kato, Y., Nagata, K., Takahashi, M., Lian, L.B., Herrero, J.J., Sudol, M., and Tanokura, M. (2004). Common mechanism of ligand recognition by group II/III WW domains - redefining their functional classification. J. Biol. Chem. *279*, 31833–31841.

Kobayashi, T., Tanaka, K., Mashima, Y., Shoda, A., Tokuda, M., and Itoh, H. (2020). CEP164 deficiency causes hyperproliferation of pancreatic cancer cells. Front. Cell Dev. Biol. *8*, 1–12.

Kumar, D., and Reiter, J. (2021). How the centriole builds its cilium: of mothers, daughters, and the acquisition of appendages. Curr. Opin. Struct. Biol. *66*, 41–48.

Laskowski, R.A., Macarthur, M.W., Moss, D.S., and Thornton, J.M. (1993). Procheck - a program to check the stereochemical quality of protein structures. J. Appl. Crystallogr. *26*, 283–291.

Laskowski, R.A., Rullmann, J.A.C., MacArthur, M.W., Kaptein, R., and Thornton, J.M. (1996). AQUA and PROCHECK-NMR: programs for checking the quality of protein structures solved by NMR. J. Biomol. NMR *8*. https://doi.org/10.1007/BF00228148.

Lee, W., Tonelli, M., and Markley, J.L. (2015). NMRFAM-SPARKY: enhanced software for biomolecular NMR spectroscopy. Bioinformatics *31*, 1325–1327.

Lo, C.H., Lin, I.H., Yang, T.T., Huang, Y.C., Tanos, B.E., Chou, P.C., Chang, C.W., Tsay, Y.G., Liao, J.C., and Wang, W.J. (2019). Phosphorylation of CEP83 by TTBK2 is necessary for cilia initiation. J. Cell Biol. *218*. https://doi.org/10.1083/jcb.201811142.

Maria, M., Lamers, I.J.C., Schmidts, M., Ajmal, M., Jaffar, S., Ullah, E., Mustafa, B., Ahmad, S., Nazmutdinova, K., Hoskins, B., et al. (2016). Genetic and clinical characterization of Pakistani families with Bardet-Biedl syndrome extends the genetic and phenotypic spectrum. Sci. Rep. *6*, 34764.

Markley, J.L., Bax, A., Arata, Y., Hilbers, C.W., Kaptein, R., Sykes, B.D., Wright, P.E., and Wuthrich, K. (1998). Recommendations for the presentation of NMR structures of proteins and nucleic acids - (IUPAC Recommendations 1998). Pure Appl. Chem. *70*, 117–142.

McCoy, A.J., Grosse-Kunstleve, R.W., Adams, P.D., Winn, M.D., Storoni, L.C., and Read, R.J. (2007). Phaser crystallographic software. J. Appl. Crystallogr. *40*, 658–674.

McDonald, C.B., McIntosh, S.K.N., Mikles, D.C., Bhat, V., Deegan, B.J., Seldeen, K.L., Saeed, A.M., Buffa, L., Sudol, M., Nawaz, Z., et al. (2011). Biophysical analysis of binding of WW domains of the YAP2 transcriptional regulator to PPXY motifs within WBP1 and WBP2 adaptors. Biochemistry *50*, 9616–9627.

Meiyappan, M., Birrane, G., and Ladias, J.A. (2007). Structural basis for polyproline recognition by the FE65 WW domain. J. Mol. Biol. *372*, 970–980. https://doi.org/10.1016/j.jmb.2007.06.064.

Mitchison, H.M., and Valente, E.M. (2017). Motile and non-motile cilia in human pathology: from function to phenotypes. J. Pathol. *241*, 294–309.

Mizianty, M.J., Peng, Z., and Kurgan, L. (2013). MFDp2: accurate predictor of disorder in proteins by fusion of disorder probabilities, content and profiles. Intrinsically Disord. Proteins *1*. https://doi.org/10.4161/idp.24428.

Murshudov, G.N., Skubak, P., Lebedev, A.A., Pannu, N.S., Steiner, R.A., Nicholls, R.A., Winn, M.D., Long, F., and Vagin, A.A. (2011). REFMAC5 for the refinement of macromolecular crystal structures. Acta Crystallogr. D Biol. Crystallogr. *67*, 355–367.

Nielsen, J.T., and Mulder, F.A.A. (2019). Quality and bias of protein disorder predictors. Sci. Rep. *9*, 5137.

Oda, T., Chiba, S., Nagai, T., and Mizuno, K. (2014). Binding to Cep164, but not EB1, is essential for centriolar localization of TTBK2 and its function in ciliogenesis. Genes Cells *19*, 927–940.

Pedersen, L.B., Veland, I.R., Schroder, J.M., and Christensen, S.T. (2008). Assembly of primary cilia. Dev. Dyn. *237*. https://doi.org/10.1002/dvdy.21521.

Pires, J.R., Parthier, C., Aido-Machado, R., Wiedermann, U., Otte, L., Bohm, G., Rudolph, R., and Oschkinat, H. (2005). Structural basis for APPTPPPLPP peptide recognition by FBP11WW1 domain. J. Mol. Biol. *348*, 399–408. https://doi.org/10.1016/j.jmb.2005.02.056.

Schmidt, K.N., Kuhns, S., Neuner, A., Hub, B., Zentgraf, H., and Pereira, G. (2012). Cep164 mediates vesicular docking to the mother centriole during early steps of ciliogenesis. J. Cell Biol. *199*, 1083–1101.

Schneider, C.A., Rasband, W.S., and Eliceiri, K.W. (2012). NIH Image to ImageJ: 25 years of image analysis. Nat. Methods *9*, 671–675. https://doi.org/10.1038/nmeth.2089.

Schwieters, C.D., Kuszewski, J.J., Tjandra, N., and Clore, G.M. (2003). The Xplor-NIH NMR molecular structure determination package. J. Magn. Reson. *160*, 65–73.

Shen, Y., Delaglio, F., Cornilescu, G., and Bax, A. (2009). TALOS plus : a hybrid method for predicting protein backbone torsion angles from NMR chemical shifts. J. Biomol. NMR *44*, 213–223.

Singla, V., and Reiter, J.F. (2006). The primary cilium as the cell's antenna: signaling at a sensory organelle. Science *313*, 629–633.

Sivasubramaniam, S., Sun, X., Pan, Y.R., Wang, S., and Lee, E.Y. (2008). Cep164 is a mediator protein required for the maintenance of genomic stability through modulation of MDC1, RPA, and CHK1. Genes Dev. *22*, 587–600.

Slaats, G.G., Ghosh, A.K., Falke, L.L., Le Corre, S., Shaltiel, I.A., van de Hoek, G., Klasson, T.D., Stokman, M.F., Logister, I., Verhaar, M.C., et al. (2014). Nephronophthisis-associated CEP164 regulates cell cycle progression, apoptosis and epithelial-to-mesenchymal transition. PLoS Genet. *10*, e1004594.

Stokman, M.F., Saunier, S., and Benmerah, A. (2021). Renal ciliopathies: sorting out therapeutic approaches for nephronophthisis. Front. Cell Dev. Biol. *9*, 653138.

Tabares da Rosa, S., Wogulis, L.A., Wogulis, M.D., González-Sapienza, G., and Wilson, D.K. (2019). Structure and specificity of several triclocarban-binding single domain camelid antibody fragments. J. Mol. Recognit. *32*, e2755. https://doi.org/10.1002/jmr.2755.

Tanos, B.E., Yang, H.J., Soni, R., Wang, W.J., Macaluso, F.P., Asara, J.M., and Tsou, M.F. (2013). Centriole distal appendages promote membrane docking, leading to cilia initiation. Genes Dev. *15*, 163–168.

Verma, A., Jing-Song, F., Finch-Edmondson, M.L., Velazquez-Campoy, A., Balasegaran, S., Sudol, M., and Sivaraman, J. (2018). Biophysical studies and NMR structure of YAP2 WW domain - LATS1 PPxY motif complexes reveal the basis of their interaction. Oncotarget *9*, 8068–8080.

Wang, S., Ma, J.Z., and Xu, J.B. (2016). AUCpreD: proteome-level protein disorder prediction by AUC-maximized deep convolutional neural fields. Bioinformatics *32*, 672–679.

Watanabe, T., Kakeno, M., Matsui, T., Sugiyama, I., Arimura, N., Matsuzawa, K., Shirahige, A., Ishidate, F., Nishioka, T., Taya, S., et al. (2015). TTBK2 with EB1/3 regulates microtubule dynamics in migrating cells through KIF2A phosphorylation. J. Cell Biol. *210*, 737–751.

Werner, S., Pimenta-Marques, A., and Bettencourt-Dias, M. (2017). Maintaining centrosomes and cilia. J. Cell Sci. *130*, 3789–3800.

Winter, G., Waterman, D.G., Parkhurst, J.M., Brewster, A.S., Gildea, R.J., Gerstel, M., Fuentes-Montero, L., Vollmar, M., Michels-Clark, T., Young, I.D., et al. (2018). DIALS: implementation and evaluation of a new integration package. Acta Crystallogr. D Struct. Biol. *74*, 85–97.

Xia, B., Tsui, V., Case, D.A., Dyson, H.J., and Wright, P.E. (2002). Comparison of protein solution structures refined by molecular dynamics simulation in vacuum, with a generalized Born model, and with explicit water. J. Biomol. NMR *22*, 317–331.

Xu, Q.W., Zhang, Y.X., Wei, Q., Huang, Y., Hu, J.H., and Ling, K. (2016). Phosphatidylinositol phosphate kinase PIPKI gamma and phosphatase INPP5E coordinate initiation of ciliogenesis. Nat. Commun. *7*, 10777.

Yang, T.T., Chong, W.M., Wang, W.J., Mazo, G., Tanos, B., Chen, Z.M., Tran, T.M.N., Chen, Y.D., Weng, R.R., Huang, C.E., et al. (2018). Super-resolution architecture of mammalian centriole distal appendages reveals distinct blade and matrix functional components. Nat. Commun. *9*, 2023.

Zarrinpar, A., and Lim, W.A. (2000). Converging on proline: the mechanism of WW domain peptide recognition. Nat. Struct. Biol. *7*, 611–613.

CellPress
OPEN ACCESS

STAR★METHODS

KEY RESOURCES TABLE

REAGENT or RESOURCE	SOURCE	IDENTIFIER
**Antibodies**		
Monoclonal ANTI-FLAG M2 antibody produced in mouse	Sigma-Aldrich/Merck	Cat# F1804
Polyclonal ANTI-HA antibody produced in rabbit	Gift from R. Hegde (MRC-LMB)	N/A
Polyclonal ANTI-GFP antibody produced in rabbit	Invitrogen/ThermoFisher Scientific	Cat# A11122
Polyclonal ANTI-ARL13B antibody produced in rabbit	Proteintech	Cat# 17711-1
ANTI-CENT1 – Alexa Fluor 647	Proteintech/Invitrogen	Cat# 12794-1-AP/ A20186
Donkey anti-Mouse IgG (H+L) Secondary Antibody, Alexa Fluor 488	Invitrogen/ThermoFisher Scientific	Cat# R37114
Donkey anti-Rabbit IgG (H+L) Highly Cross-Adsorbed Secondary Antibody, Alexa Fluor 568	Invitrogen/ThermoFisher Scientific	Cat# A10042
Normal Rabbit IgG	Santa Cruz Biotechnology	Cat# sc-2027
**Bacterial Strains**		
Rosetta (DE3)	Gift from J. Kilmartin (MRC-LMB)	N/A
**Chemicals, Peptides, and Recombinant Proteins**		
Glycergel Mounting Media	Agilent	Cat# C056330-2
Lipofectamine 3000	Thermo Fisher Scientific	Cat# L3000001
D-MEM/F-12, supplied,GlutaMAX, sodium carbonate	Thermo Fisher Scientific	Cat# 31331028
D-MEM Glutamax	Thermo Fisher Scientific	Cat# 10566016
Expi293 Expression Medium	Thermo Fisher Scientific	Cat# A1435101
Glutathione sepharose 4B	GE Healthcare Life Sciences	Cat# 17075601
Amylose resin	New England Biolabs	Cat# E8021S
anti-FLAG M2 magnetic beads	Sigam-Aldrich/Merck	Cat# M8823
NuPAGE 4-12% Bis-Tris 1.0mm 15-well	ThermoFisher Scientific	Cat# NP0323BOX
PEI	Polysciences	Cat# 24765
TTBK2$^{1074\text{-}1087}$ peptide	Biomatik	N/A
Wizard Classic 2 screen	Rigaku/ Molecular Dimensions	Cat# MD15-W2-T
JCSG screen	Qiagen/ NeXtal	Cat# 130920
Wizard Cryo 2 screen	Rigaku/ Molecular Dimensions	Cat# MD15-C2-T
**Deposited Data**		
Crystal structure of *Homo sapiens* CEP164$^{1\text{-}109}$ in complex with camelid nanobody 10Z	This paper	PDB accession code 7O06
Crystal structure of *Homo sapiens* CEP164$^{1\text{-}109}$ in complex with camelid nanobody 36Z	This paper	PDB accession code 7O0S
Crystal structure of *Homo sapiens* TTBK2$^{1074\text{-}1087}$-CEP164$^{1\text{-}109}$ in complex with camelid nanobody 36Z	This paper	PDB accession code 7O3B
NMR structure ensemble of *Homo sapiens* CEP164$^{1\text{-}109}$	This paper	PDB accession code 7NWJ
NMR data	This paper	BMRB accession code 50793

(*Continued on next page*)

Continued		
REAGENT or RESOURCE	SOURCE	IDENTIFIER
Crystal structure of the camelid nanobody VHH T9	Tabares da Rosa et al. (2019)	PDB accession code 5VLV
Crystal structure of human EB1 in complex with microtubule Tip localization signal peptide of MACF	Honnappa et al. (2009)	PDB accession code 3GJO
Crystal structure of ADP bound TTBK2 kinase domain	Bao et al. (2021)	PDB accession code 6VRF
NMR structure of Smurf1 WW domain in complex with a Smad1 derived peptide	Aragón et al. (2011)	PDB accession code 2LAZ
NMR structure of YAP WW domain in complex with a Smad1 derived peptide	Aragón et al. (2011)	PDB accession code 2LAW
NMR structure of FBP11WW1 domain in complex with a proline-rich peptide	Pires et al. (2005)	PDB accession code 1YWI
Crystal structure of FE65 WW domain in complex with hMena peptide	Meiyappan et al. (2007)	PDB accession code 2HO2
Experimental Models: Cell Lines		
hTERT RPE-1	Gift from C. Morrison (NUI Galway) (Daly et al., 2016)	N/A
CEP164 null hTERT RPE-1	Gift from C. Morrison (NUI Galway) (Daly et al., 2016)	N/A
Flp-In T-REx 293	Gift from R. Hegde (MRC-LMB) (Joseph et al., 2018)	N/A
Expi293	Gift from O. Perisic, R. Williams group (MRC-LMB)	N/A
Recombinant DNA		
pGEX6P1-CEP164$^{1-109}$ (BamH1 and EcoR1 sites)	This paper	N/A
pGEX6P1-TTBK2$^{1074-1087}$-CEP164$^{1-109}$ (BamH1 and EcoR1 sites)	This paper	N/A
pGEX6P1-CEP1641-28,50-94 (=ΔHelices)-TEV-HIS6 (BamH1-EcoR1 sites)	This paper	N/A
pGEX6P1-camelid nanobody 10Z (BamH1 and EcoR1 sites)	This paper	N/A
pGEX6P1-camelid nanobody 36Z (BamH1 and EcoR1 sites)	This paper	N/A
pOPTM -TTBK2$^{1074-1087}$ (Nde1 and BamH1 sites)	This paper	N/A
pGEX6P1- EB1 (BamH1 and EcoR1 sites)	This paper	N/A
pSKB2LNB-EB1-GFP (Nde1 and BamH1 sites)	This paper	N/A
pGEX6P1-CEP164N-GFP (BamH1 and EcoR1 sites)	This paper	N/A
pcDNA 3.1 derivative – 3xFLAG-TTBK2-3xSTREP (WT or D163A mutant) (BamH1 and EcoR1 sites)	This paper	N/A
pcDNA 3.1 derivative – 3xFLAG-CEP164 (WT or Q11P, or R93W, or kinase dead (BamH1 and EcoR1 sites)	This paper	N/A
pcDNA 3.1 derivative – 3xHA-TTBK2 (or mutant) (BamH1 and EcoR1 sites)	This paper	N/A
Software and Algorithms		
DIALS	(Winter et al., 2018)	https://dials.github.io/
AIMLESS	(Evans and Murshudov, 2013)	http://www.ccp4.ac.uk/download

(Continued on next page)

Continued

REAGENT or RESOURCE	SOURCE	IDENTIFIER
PHASER	(McCoy et al., 2007)	http://www.ccp4.ac.uk/download
REFMAC	(Murshudov et al., 2011)	http://www.ccp4.ac.uk/download
COOT	(Emsley and Cowtan, 2004)	http://www.ccp4.ac.uk/download
PHENIX.REFINE	(Afonine et al., 2012)	http://www.phenix-online.org/download/
MolProbity	(Chen et al., 2010)	http://molprobity.manchester.ac.uk/
The PyMOL Molecular Graphics System	Schrödinger, LLC	Version 2.0 / https://pymol.org/2/#download
GraphPad Prism Software	GraphPad Software	Version 6.0 / https://www.graphpad.com/
AUCpreD	(Wang et al., 2016)	http://raptorx.uchicago.edu/StructurePropertyPred/predict/
Multilayered Fusion-based Disorder predictor (MFDp2)	(Mizianty et al., 2013)	http://biomine.cs.vcu.edu/servers/MFDp2/
SPOT-Disorder2	(Hanson et al., 2019)	https://sparks-lab.org/server/spot-disorder2/
Malvern Panalytical PEAQ software	Malvern	N/A
TopSpin	Bruker	Version 3
NMRFAM-SPARKY	(Lee et al., 2015)	https://nmrfam.wisc.edu/nmrfam-sparky-distribution/
XPLOR-NIH	(Schwieters et al., 2003)	https://nmr.cit.nih.gov/xplor-nih/
TALOS+	(Shen et al., 2009)	https://spin.niddk.nih.gov/bax/nmrserver/talos/
AMBER 11	(Case et al., 2005)	https://ambermd.org/GetAmber.php
CLUSTERPOSE	(Diamond, 1995)	https://www.ccpn.ac.uk/v2-software/software/extras/contributions
PROCHECK-NMR	(Laskowski et al., 1993; Laskowski et al., 1996)	https://www.ebi.ac.uk/thornton-srv/software/PROCHECK/
DeltaVision softWoRx	GE Healthcare Life Sciences	N/A
Other		
Sephacryl S-300 column	GE Healthcare Life Sciences	Cat# GE17-0599-01
Hi-Trap Q-FF column	GE Healthcare Life Sciences	Cat# 17515601
Hi-Trap Q-HP column	GE Healthcare Life Sciences	Cat# 17115401
Superdex S75 HR 10/30 column	GE Healthcare Life Sciences	Cat# 17-1047-01
Hi-Trap SP-FF column	GE Healthcare Life Sciences	Cat# 17-5157-01
Ni-NTA	Qiagen	Cat# 30210
Complete Protease Inhibitor cocktail (EDTA free)	Roche/Merck	Cat# 11873580001
Malvern Panalytical autoITC	Malvern	MRC-LMB
Jasco 815 Spectropolarimeter	JASCO	MRC-LMB
Bruker Avance-III spectrometers operating at 1H frequencies of 600, 700 or 800 MHz	Bruker	MRC-LMB
DeltaVision Elite microscope	GE Healthcare Life Sciences	National Centre for Biomolecular Research, Faculty of Science, Masaryk University

RESOURCE AVAILABILITY

Lead contact

Further information and requests for resources and reagents should be directed to and will be fulfilled by M. van Breugel (m.vanbreugel@qmul.ac.uk).

Materials availability

All unique reagents generated in this study are available from the Lead Contact.

Data and code availability

Coordinates and structure factors of crystal structures that are presented in this paper are available in the Protein Data Bank (PDB, accession codes: Nanobody 10Z-CEP164$^{1-109}$ - 7O06, Nanobody 36Z-CEP164$^{1-109}$ - 7O0S, Nanobody 36Z-TTBK2$^{1074-1087}$-CEP164$^{1-109}$ - 7O3B). NMR data are deposited in the Biological Magnetic Resonance Bank (BMRB, accession code 50793). NMR structure ensemble of CEP164$^{1-109}$ coordinates are deposited in PDB (accession code 7NWJ). This paper does not report original code. Any additional information required to reanalyse the data reported in this paper is available from the lead contact upon request.

EXPERIMENTAL MODEL AND SUBJECT DETAILS

Cell lines

Human cell culture

Flp-In T-REx 293 cells (gift from R. Hegde, MRC-LMB, Cambridge, UK) were grown in D-MEM, GlutaMAX (Thermo Fisher Scientific) supplied with 10% FBS. CEP164-null hTERT RPE-1 (gift from C. Morrison, NUI Galway, Ireland) cells were grown in D-MEM/F-12 (Thermo Fisher Scientific), GlutaMAX, sodium carbonate (ThermoFisher Scientific) supplied with 0, 0.5 or 10% FBS. Expi293 cells (gift from O. Perisic from R. Williams lab, MRC-LMB, Cambridge, UK) were grown in Expi293 medium (ThermoFisher). All cell lines were grown at 37°C with 5% CO_2.

Bacterial cell culture

Escherichia coli Rosetta (DE3) (gift from J. Kilmartin, MRC-LMB, Cambridge, UK) were grown in LB or 2xTY media and used for protein expression and purification.

METHOD DETAILS

Recombinant protein purification

DNA encoding human CEP164$^{1-109}$ (Uniprot Q9UPV0) was cloned into vector pGEX6P1 cut by BamH1-EcoR1 restriction enzyme digestion, giving rise to an ORF with an N-terminal GST-tag that can be removed by cleavage with PreScission protease. Mutations or deletions were introduced into this construct by site-directed mutagenesis. Constructs (or empty pGEX6P1) were expressed in *E. coli* Rosetta (DE3) cells at 18°C by IPTG induction overnight. Subsequently, the proteins were purified from cell lysates (prepared by sonication and centrifugational clearing) by chromatography with Glutathione-Sepharose 4B (GE Healthcare) and eluted with reduced Glutathione using standard methods. Unlike for the preparation of the GST-tagged versions, for the preparation of the untagged CEP164 constructs, purified GST-PreScission protease was added and the eluates dialysed against 10 mM Tris-Cl pH 8.0, 50 mM NaCl, 2 mM DTT. Subsequently, the eluates were subjected to gel-filtration on a Sephacryl S-300 column (GE Healthcare) run in 10 mM Tris-Cl pH 8.0, 50 mM NaCl, 2 mM DTT. Peak fractions were pooled and subjected to ion-exchange chromatography on a Hi-Trap Q-FF or -HP column (GE Healthcare) run using a linear salt gradient from 10 mM Tris-Cl, pH 8.0, 2 mM DTT (buffer A) to buffer A, 1M NaCl over 25 column volumes. Peak fractions were concentrated, snap frozen in small aliquots and stored at -80°C. For the untagged CEP164 constructs that were used in ITC or thermostability experiments or for crystallisations, a final gel-filtration step was performed on a Superdex S75 column (Pharmacia) either run in ITC buffer (PBS, 2 mM DTT, ITC or thermostability assay) or in 10 mM Na-Hepes pH 7.2, 50 mM NaCl, 2 mM DTT (crystallisation). The proteins were concentrated, snap frozen in aliquots and stored at -80°C.

^{15}N- or $^{15}N^{13}C$-labelled CEP164$^{1-109}$ proteins were expressed as above, but in supplemented M9 medium (van Breugel et al., 2014) containing $^{15}NH_4Cl$ and optionally ^{13}C Glucose. Proteins were purified by Glutathione-Sepharose 4B (GE Healthcare) chromatography, followed by GST-PreScission protease addition, an optional gel-filtration step on a Sephacryl S-300 column (GE Healthcare) run in 10 mM Tris-Cl pH 8.0, 50 mM NaCl, 2 mM DTT, followed by ion-exchange chromatography on a Hi-Trap Q-FF or Q-HP column (GE Healthcare) run using a linear salt gradient from 10 mM Tris-Cl, pH 8.0, 4 mM DTT (buffer A) to buffer A, 1M NaCl over 25 column volumes. Peak fractions were concentrated and a final gel-filtration step performed on a Superdex S75 column (Pharmacia) run in NMR buffer (PBS, 4 mM DTT, supplemented with Complete Protease Inhibitor cocktail (EDTA free, Roche)). The proteins were concentrated, snap frozen in aliquots and stored at -80°C.

The fusion construct TTBK2$^{1074-1087}$-CEP164$^{1-109}$ (Uniprot Q6IQ55 / Q9UPV0 respectively) was cloned into vector pGEX6P1 cut by BamH1-EcoR1 restriction enzyme digestion, giving rise to an ORF with an N-terminal GST-tag that can be removed by cleavage with PreScission protease. This construct had the linker AGSGAGS placed between TTBK2$^{1074-1087}$ and CEP164$^{1-109}$. Expression and Glutathione-Sepharose purification were as described above, but instead of elution with Glutathione, the protein was eluted by on-beads cleavage with GST-PreScission protease in 50 mM Tris-Cl, pH 7.4, 2 mM DTT. This was followed by ion-exchange chromatography on a Hi-Trap Q-FF column (GE Healthcare) run using a linear salt gradient from 10 mM Tris-Cl, pH 8.0, 2 mM DTT (buffer A) to buffer A, 1M NaCl over 25 column volumes. Peak fractions were concentrated and a final gel-filtration step performed on a Superdex S75 column (Pharmacia) run in 10 mM Na-Hepes pH 7.2, 50 mM NaCl, 2 mM DTT. The proteins were concentrated, snap frozen in aliquots and stored at -80°C.

Construct CEP164$^{1-28,50-94}$ (=ΔHelices)-TEV-HIS6 was cloned into vector pGEX6P1 cut by BamH1-EcoR1 restriction enzyme digestion, giving rise to an ORF with an N-terminal GST-tag that can be removed by cleavage with PreScission protease and a C-terminal His-tag that can be removed by cleavage with TEV protease. The construct was expressed in *E. coli* Rosetta (DE3) cells at 18°C by IPTG induction overnight. Subsequently, the protein was purified from cell lysates (prepared by sonication and centrifugational

clearing) by Ni-NTA (Qiagen) chromatography using standard methods. After elution, the eluate was dialysed overnight against cleavage buffer (50 mM Tris-HCl, pH 8.0, 500 mM NaCl, 5 mM imidazole, pH 7.5, 2 mM DTT) in the presence of TEV protease. Subsequently, the reactions were allowed to bind to Glutathione-Sepharose 4B (GE Healthcare) beads, the beads washed with cleavage buffer, and the protein eluted by on-beads cleavage with GST-PreScission protease in PBS, 2 mM DTT. After a gel-filtration step on a Superdex S75 column (Pharmacia) in PBS, 2 mM DTT, the peak fractions were concentrated and the protein snap frozen and stored at -80°C.

Camelid Nanobodies 10Z and 36Z were cloned into vector pGEX6P1 cut by BamH1-EcoR1 restriction enzyme digestion, giving rise to an ORF with an N-terminal GST-tag that can be removed by cleavage with PreScission protease. Expression and Glutathione-Sepharose purification were as described above. Purified GST-PreScission protease was added and the eluates dialysed against 10 mM Tris-Cl pH 7.4. Na-Acetate pH 5.3 (10Z) or (optionally) Na-Bicine pH 9.0 (36Z) were added to 10 mM followed by ion-exchange chromatography on a Hi-Trap S-FF (10Z) or Q-FF (36Z) column (GE Healthcare) run using a linear salt gradient from 10 mM Na-Hepes pH 7.2 (buffer A) (10Z) or 10 mM Tris-Cl, pH 8.0 or pH 8.5 (buffer A) (36Z) to buffer A, 1M NaCl over 25 column volumes. Peak fractions were concentrated and a final gel-filtration step performed on a Superdex S75 column (Pharmacia) run in 10 mM Na-Hepes pH 7.2, 50 mM NaCl, (optionally) 2 mM DTT. The proteins were concentrated, snap frozen in aliquots and stored at -80°C.

The peptide corresponding to $TTBK2^{1074-1087}$ was synthesised by Biomatik (Wilmington, Delaware, 19809 USA) to a purity of 98% as judged by HPLC analysis and was reconstituted with Millipore dH2O. Amino acid analysis (New England Peptide, Gardner, MA 01440 USA) was used in duplicate to determine the accurate concentration of the peptide stock in solution (4.844 mM).

MBP-$TTBK2^{1033-1087}$ (wild-type, EB1-binding deficient $TTBK2^{1051-1054\ SKIP\ to\ AAAA}$ or CEP164-NTD-binding deficient $TTBK2^{1076-1078\ RPP\ to\ AAA}$) in vector pOPTM (R. Williams lab, MRC-LMB) were expressed in *E. coli* Rosetta (DE3) cells at 18°C by IPTG induction and purified from cell lysates by chromatography with Amylose resin (NEB, E8021) and eluted with Maltose using standard methods. Eluates were subjected to ion-exchange chromatography on a Hi-Trap Q-HP column (GE Healthcare) run using a linear salt gradient from 10 mM Tris-Cl, pH 8.0, 2 mM DTT (buffer A) to buffer A, 1M NaCl over 25 column volumes. Finally, the eluates were run in PBS, 2 mM DTT on a Superdex 200 column (GE Healthcare). Peak fractions were concentrated, snap frozen in small aliquots and stored at -80°C.

EB1 (in vector pGEX6P1) was expressed in *E. coli* Rosetta (DE3) cells at 18°C by IPTG induction, purified by chromatography with Glutathione-Sepharose 4B (GE Healthcare) and eluted with reduced Glutathione using standard methods. EB1-GFP (in a modified pET28 vector) was expressed similarly and purified by chromatography with NiNTA resin (Qiagen) and eluted with Imidazole using standard methods. Purified GST-PreScission protease was added to the eluates and eluates dialysed against 10 mM Tris-Cl pH 8.0, 2 mM DTT. Subsequently, the eluates were subjected to ion-exchange chromatography on a Hi-Trap Q-HP column (GE Healthcare) run using a linear salt gradient from 10 mM Tris-Cl, pH 8.0, 2 mM DTT (buffer A) to buffer A, 1M NaCl over 25 column volumes. Finally, the eluates were run in PBS, 2 mM DTT on a Superdex 200 column (GE Healthcare). Peak fractions were concentrated, snap frozen in small aliquots and stored at -80°C. CEP164-NTD ($CEP164^{1-109}$, optionally C-terminally tagged with GFP, both constructs in vector pGEX6P1) that was used in the competition assay was purified as described above and equally subjected to a final gel-filtration step in PBS, 2 mM DTT on a Superdex 200 column (GE Healthcare).

Full-length 3xFLAG-TTBK2-2xStrep-tag II (Uniprot Q6IQ55, natural variant L8P) was expressed in Expi293 cells by transient transfection for 48h, and purified from cells lysates (prepared by sonication and centrifugational clearing) by chromatography with anti-FLAG M2 Affinity gel (Sigma, A2220) and eluted with 3xFLAG peptide (Sigma, F4799) in 50 mM HEPES pH 8.0, 100 mM NaCl, 0.1% IGEPAL CA-630, 0.5 mM DTT. Eluates were incubated in the presence of Lambda-Protein-Phosphatase (Santa Cruz) with Strep-Tactin Sepharose (IBA) beads, beads washed with 50 mM HEPES pH 8.0, 100 mM NaCl, 0.1% IGEPAL CA-630, 2 mM DTT and then eluted with 6 mM D-Desthiobiotin (IBA) in 50 mM HEPES, 100 mM NaCl, 0.1% IGEPAL CA-630, 2 mM DTT, pH 7.8. The eluted proteins were snap frozen in small aliquots and stored at -80°C.

Nanobody generation

Nanobodies against recombinant, purified human $CEP164^{1-109}$ were supplied by VIB Nanobody Core (Vrije Universiteit Brussel (VUB), 1050 Brussels). They were raised by injections of purified $CEP164^{1-109}$ into a llama, followed by construction of a VHH library from peripheral blood lymphocytes for phage display, phage display panning against $CEP164^{1-109}$ and analysis of clones by anti-$CEP164^{1-109}$ ELISA and sequencing.

Protein crystallization

Nanobody 10Z/36Z and $CEP164^{1-109}$ were mixed equimolar to a final concentration of 0.81 mM (Nanobody 10Z-$CEP164^{1-109}$ complex) or 1.16 mM (Nanobody 36Z-$CEP164^{1-109}$ complex). Crystals of the corresponding protein-complexes were obtained in the Wizard 2 screen (Emerald BioStructures, reservoir solution: 0.1 M Hepes, pH 7.5, 1.26 M ammonium sulfate) (10Z-$CEP164^{1-109}$) or in the JCSG+ suite (Qiagen, reservoir solution: 0.18 M tri-ammonium citrate, 20 % (w/v) PEG 3350) (36Z-$CEP164^{1-109}$) by the vapour diffusion method at 19°C using 100 nl protein solution and 100 nl of reservoir solution. Crystals were mounted after six days in the mother liquor, to which PEG-400 was added to 30%, and frozen in liquid nitrogen.

Nanobody 36Z and the $TTBK2^{1074-1087}$-$CEP164^{1-109}$ fusion protein were mixed equimolar to a final concentration of 1.38 mM. Crystals of the corresponding protein-complex were obtained in the Emerald BioStructures Cryo 2 Screen (reservoir

solution: 100 mM Acetate, pH 4.5, 100 mM NaCl, 30 % PEG-200) by the vapour diffusion method at 19°C using 100 nl protein solution and 100 nl of reservoir solution. Crystals were mounted after seven days in the mother liquor and frozen in liquid nitrogen.

X-ray crystallography data processing

Datasets were integrated using DIALS (Winter et al., 2018) and were scaled using AIMLESS (Evans and Murshudov, 2013). The structure of the Nanobody 10Z-CEP164$^{1\text{-}109}$ complex was solved by molecular replacement with PHASER (McCoy et al., 2007) using the nanobody of PDB 5VLV (with removed loops and as poly-alanine model, Tabares da Rosa et al., 2019) as a search model. Cycles of refinement in REFMAC (Murshudov et al., 2011) and manual building in COOT (Emsley and Cowtan, 2004) allowed the complete building of the nanobody 10Z structure. The electron density maps showed clear electron densities in regions not occupied by the nanobody that tentatively could be assigned to CEP164$^{1\text{-}109}$ residues. Placement of these residues and further refinement in REFMAC (Murshudov et al., 2011) and PHENIX.REFINE (Afonine et al., 2012) and manual building cycles in COOT (Emsley and Cowtan, 2004) allowed the successive building of a complete model of CEP164$^{1\text{-}109}$ with high confidence.

The structure of the Nanobody 36Z-CEP164$^{1\text{-}109}$ complex was solved by molecular replacement with PHASER (McCoy et al., 2007) using the nanobody of PDB 5VLV (with removed loops and as poly-alanine model, Tabares da Rosa et al., 2019) and the CEP164$^{1\text{-}109}$ structure as search models. Cycles of refinement in REFMAC (Murshudov et al., 2011) and PHENIX.REFINE (Afonine et al., 2012) and manual building in COOT (Emsley and Cowtan, 2004) were used to build the structure of the Nanobody 36Z-CEP164$^{1\text{-}109}$ complex.

The structure of the complex between Nanobody 36Z and the TTBK2$^{1074\text{-}1087}$-CEP164$^{1\text{-}109}$ fusion protein was solved by molecular replacement with PHASER (McCoy et al., 2007) using the nanobody 36Z and the CEP164$^{1\text{-}109}$ structure as search models. Cycles of refinement in REFMAC (Murshudov et al., 2011) and PHENIX.REFINE (Afonine et al., 2012) and manual building in COOT (Emsley and Cowtan, 2004) were used to build the structures of nanobody 36Z and of CEP164$^{1\text{-}109}$. The electron density maps showed clear electron densities close to the surface of the WW domain of CEP164$^{1\text{-}109}$ that were not occupied by the nanobody or CEP164$^{1\text{-}109}$. Due to a high connectivity and clear side-chain densities for TTBK2 R1076 and most of the TTBK2$^{1074\text{-}1087}$ proline residues, these electron densities allowed the confident building of TTBK2 residues 1074-1084 in further refinement and building cycles.

Structure visualization and measurement of the angle between CEP164$^{1\text{-}109}$ head and body structures were done in PyMOL (The PyMOL Molecular Graphics System, Version 2.0 Schrödinger, LLC). GraphPad Prism Software v. 6.0 (GraphPad Software; www.graphpad.com) was used to plot the result.

Pull-down assays

Pull-downs with full-length 3xFLAG CEP164 constructs

Flp-In T-REx 293 cells were transfected with a pcDNA 3.1 derivative vector (gift from R. Hegde, MRC-LMB) expressing (from a CMV promoter) 3xFLAG tagged human CEP164 (Uniprot Q9UPV0, natural variant T988S) or 3xHA tagged human TTBK2 (Uniprot Q6IQ55, natural variant L8P) constructs using Polyethylenimine (PEI MAX, MW 40000, Polysciences). Cells were lysed by sonication in lysis buffer (PBS, 0.1% (v/v) IGEPAL CA-630, supplemented with Complete Protease Inhibitor (EDTA free, Roche)) and lysates were cleared by centrifugation. Cleared lysates containing the 3xFLAG-tagged CEP164 constructs were added to anti-FLAG M2 magnetic beads (M8823, Sigma). After incubation on a rotator for 1h at 4°C, beads were washed with lysis buffer and the cleared lysates containing the 3xHA-tagged human TTBK2 constructs were added to the beads. After another incubation on a rotator for 1h at 4°C, beads were washed with lysis buffer, eluted with SDS and eluates subjected to Western blotting using a mouse monoclonal anti-FLAG antibody (F1804, Sigma) or a polyclonal rabbit antibody against the HA-tag (gift from R. Hegde, MRC-LMB, Cambridge, UK).

Pull-downs with GST-CEP164$^{1\text{-}109}$

Flp-In T-REx 293 cells were transfected with vector pEGFP-N1 containing TTBK2$^{1074\text{-}1087}$ constructs (cloned into pEGFP-N1 utilising the Xho1-BamH1 restriction sites) using Polyethylenimine (PEI MAX, MW 40000, Polysciences). Cells were lysed by sonication in lysis buffer (PBS, 0.1% (v/v) IGEPAL CA-630, 2 mM DTT, supplemented with Complete Protease Inhibitor (EDTA free, Roche)) and lysates were cleared by centrifugation. Cleared lysates were added to Glutathione-Sepharose 4B beads (GE Healthcare) onto which 60 μg of purified GST or GST-CEP164$^{1\text{-}109}$ constructs had been bound beforehand (in lysis buffer). After incubation on a rotator for 1h at 4°C, beads were washed with lysis buffer and eluted with Laemmli Buffer. Eluates were subjected to Western blotting using a polyclonal GFP antibody (Invitrogen, A11122).

Pull-downs with 3xFLAG EB1 constructs

Flp-In T-REx 293 cells were transfected with a pcDNA 3.1 derivative vector expressing (from a CMV promoter) 3xFLAG only, 3xFLAG tagged human EB1 or 3xHA tagged human TTBK2 (WT or ΔPro-rich (Δ1074-1087)) constructs, using Polyethylenimine (PEI MAX, MW 40000, Polysciences). Cells were lysed by sonication in lysis buffer (PBS, 0.1% (v/v) IGEPAL CA-630, 0.1 mM DTT, supplemented with Complete Protease Inhibitor (EDTA free, Roche)) and lysates were cleared by centrifugation. Cleared lysates containing the 3xFLAG or the 3xFLAG tagged EB1 construct were added to anti-FLAG M2 affinity gel (A2220, Sigma). After incubation on a rotator for 1h at 4°C, beads were washed with lysis buffer and the centrifugationally cleared cell lysates containing the 3xHA-tagged human TTBK2 constructs, mixed with ~ 0.4 mg/ml recombinant GST-CEP164$^{1\text{-}109}$ (WT or Y73A, expressed in *E.coli* Rosetta, purified by glutathione affinity chromatography and dialysed against PBS, 1 mM DTT), were added to the beads. After incubation on a rotator for 1h at 4°C, beads were washed with lysis buffer, eluted with SDS and eluates subjected to Western blotting using a mouse monoclonal anti-FLAG antibody (F1804, Sigma) or a polyclonal rabbit antibody against the HA-tag (gift of R. Hegde, MRC-LMB, Cambridge, UK).

Pull-downs with MBP-TTBK2 constructs

A total of 100 μg of purified CEP164-NTD-GFP or 100 μg of purified EB1 (optionally in the presence of a 10X molar excess of purified CEP164-NTD) was added in 300 μl lysis buffer (PBS, 0.1% IGEPAL CA-630, 2 mM DTT) to Amylose resin (New England Biolabs) onto which 30 μg of purified MBP-TTBK2 constructs (MBP-TTBK2$^{1033-1087}$ wild-type, EB1-binding deficient TTBK2$^{1051-1054}$ SKIP to AAAA, or CEP164-NTD-binding deficient TTBK2$^{1076-1078}$ RPP to AAA) had been bound beforehand. After incubation on a rotator for 1h at 4°C, beads were washed with lysis buffer and eluted with Laemmli Buffer. Eluates were subjected to electrophoresis in a NuPAGE 4-12% gel (Invitrogen) and stained with Coomassie brilliant blue. In a similar set-up, we tested whether the presence of purified EB1-GFP (in 10X molar excess) would affect CEP164-NTD-GFP binding to these MBP-TTBK2 constructs .

Autophosphorylation assay

Purified dephosphorylated full-length 3xFLAG-TTBK2-2xStrep-tag II (wild-type or kinase dead (KD, D163A)) produced in Expi293 cells were submitted to an auto-phosphorylation assay. A total of 30 μg of recombinant kinase was incubated with 2.0 mM ATP and 2.0 mM $MgCl_2$ in a buffer containing 50 mM HEPES pH 8.0, 100 mM NaCl, 2 mM DTT (total reaction volume of 35 μl) for 10 minutes on ice in the presence or in the absence of 120 μg of purified CEP164-NTD WT or Y73A mutant. The reaction was quenched by adding 10 μl Laemmli buffer and the product was analysed in a 8% SDS-PAGE (Tris-Glycine) gel stained with Coomassie brilliant blue.

Bioinformatics

Intrinsically disordered regions were predicted using the following methods: AUCpreD (Wang et al., 2016), Multilayered Fusion-based Disorder predictor (MFDp2,(Mizianty et al., 2013)) and SPOT-Disorder v1.0 (Hanson et al., 2017, 2019). The theoretical mean end-to-end distance of protein intrinsically disordered regions was calculated based on the method described in (Fitzkee and Rose, 2004).

Circular dichroism (CD)

Measurements were made using a Jasco 815 Spectropolarimeter with a 0.1 cm cuvette and in PBS buffer. Spectra were recorded at 0.2 mg/ml concentration using a scan rate of 50 nm/min, bandwidth 1nm and were the average of 8 scans. Mean residue ellipticity was calculated using

$$\Theta_{mrw} = 0.1 \times \Theta/([\text{conc.}] \times N \times PL)$$

where Θ is the measured signal in millidegree, N is the number of residues and PL the pathlength in cm, thereby allowing direct comparison of the constructs of differing sequence lengths.

Thermal denaturation was followed at 222 nm with a bandwidth of 4 nm between 4 and 95°C and using a scan rate of 1°C/min. Sample concentration was 0.2 mg/ml and Θ_{mrw} was calculated as above.

Isothermal titration calorimetry (ITC)

Binding interactions were studied using a Malvern Panalytical autoITC instrument at 25°C and in PBS buffer. The ITC method is particularly sensitive to errors in the concentration of the ligand titrated from the instrument syringe added in excess of the binding partner in the cell. For this reason, we chose to perform ITC experiments with the CEP164$^{1-109}$ protein in the syringe and with the target binding peptide (which contains no aromatics that would allow its quantification using UV absorbance) in the cell. We were able to concentrate the protein up to the 1-3 mM stock concentrations required for the syringe with no evidence of aggregation.

ITC experiments were performed with 19 injections of 2 μL preceded by a small 0.5 μL pre-injection that was not used during curve fitting. Control measurements of injections of CEP164$^{1-109}$ protein into buffer were performed (WT and mutants where stocks of materials permitted) and these control heats were close to the values seen for buffer into buffer blank runs. All ITC binding data were corrected with the appropriate control heats of dilution of CEP164$^{1-109}$ and fitted using the one set of binding sites' model in Malvern Panalytical PEAQ software.

In our ITC experiments, the binding stoichiometry of the TTBK2-CEP164 (wild-type) complex was 0.7 (± 0.2 SD, n=5) across different batches of CEP164$^{1-109}$ preparations. This stoichiometry is lower than 1, the value expected for the 1:1 complexes typically formed between WW domains and proline-rich binding motifs (PRBMs) and seen in the crystal structure of the TTBK2-CEP164 complex (Figure 3A). This discrepancy is not large and may simply result from the fact that the fitted stoichiometry is poorly constrained where titrations are performed with material in the ITC cell at or below the K_D concentration (ITC c-value ~1). It was not possible to perform experiments at higher concentrations due to sample limitation and concerns over aggregation. However, we also note the preponderance of prolines in the TTBK2 peptide that will be present in various combinations of cis-trans isomerization states in this unstructured peptide. Some fraction of these states may be unable to bind CEP164 and their re-isomerization may be slow on the timescale of ITC measurement thereby resulting in the underestimated binding stoichiometry that then reflects binding competent fraction of peptide.

Stock peptide solution in the ITC cell was prepared by weight of lyophilized material to a notional 100 μM. This stock used for all ITC measurements was then quantified using a commercial amino acid analysis service provided by New England Peptide (Gardner, MA 01440 USA).

NMR

NMR spectra were recorded using Bruker Avance-III spectrometers operating at 1H frequencies of 600, 700 or 800 MHz, each equipped with 5 mm inverse cryogenically cooled probes, and with a sample temperature of 293 K unless otherwise stated. All samples were prepared in physiological ionic strength buffer (25 mM aqueous sodium phosphate, 125 mM sodium chloride, at pH 7.4, supplemented with 4 mM DTT and 1 pill of Complete Protease Inhibitor cocktail (EDTA free, Roche) per 250 ml) and degassed by repeated cycles of vacuum or argon in the head space. Backbone and Cβ resonance assignments were obtained for a 120 μM sample of ^{13}C- and ^{15}N-labelled protein, using standard triple resonance techniques and unmodified Bruker pulse programs. Aliphatic sidechain resonance assignments were recorded from a 2D ^{1}H-^{13}C-HSQC, aided by 3D (H)CCH- and H(C)CH-TOCSY spectra. Sidechain resonances of Tyr and Phe residues were assigned using 2D ^{1}H-^{13}C-HSQC selective for the aromatic region, and (HB)CB(CGCD)HD and (HB)CB(CGCC-TOCSY)H spectra, and Trp sidechain assignments from 2D NOESY and DQF-COSY. Chemical shifts were referenced to the ^{1}H frequency of internal 210 μM dimethylsilapentanesulfonate (DSS) added to a 250 μM ^{15}N-labelled sample, with X-nuclei referenced according to IUPAC recommendations (Markley et al., 1998).

For structure determination NOE intensities were recorded for 500 μM ^{15}N-labelled protein using a 2D ^{1}H-^{1}H-NOESY with 120 ms NOE mixing time (τ_m) and excitation sculpting water suppression, with 1.5 seconds repetition delay, 512 and 2048 data points in t_1 and t_2, respectively and spectral widths of 8.4 kHz in both dimensions. Assignment was supported by 3D ^{13}C-edited and ^{15}N-edited ^{1}H-^{1}H-NOESY-HSQC spectra recorded with 100 ms τ_m, 800 MHz ^{1}H.

Steady state {^{1}H}^{15}N-NOE data were acquired in duplicate as pseudo-3D spectra at 600 MHz ^{1}H, interleaving rows with or without 120° ^{1}H pulses applied at 5 ms intervals throughout the 7 seconds recycle delay. Samples were ^{15}N-labelled, at concentrations of 500 μM for WT protein, 320 μM for the Q11P mutant.

All NMR datasets were processed using TopSpin version 3 (Bruker) and analysed using NMRFAM-SPARKY (Lee et al., 2015).

NMR structure calculations

Initial structures were calculated using XPLOR-NIH (Schwieters et al., 2003). Distance restraints were categorised as strong (0-2.2 Å), medium (0-3.0 Å), weak (0-4.0 Å) or very weak (0-5.5 Å), based on NOE intensity. Since the XPLOR-NIH calculations employed r^{-6} summation for all groups of equivalent protons and non-stereospecifically assigned prochiral groups, and since no stereoassignments were made (and the assignment-swapping protocol within XPLOR-NIH for deriving stereoassignments indirectly during the structure calculation itself was not applied), all distance restraints involving protons within such groups were converted to group restraints (by using wildcards such as HB*). All lower bounds were set to zero (Hommel et al., 1992). Backbone φ and ψ torsion angle restraints were derived from chemical shift indices, using the program TALOS+ (Shen et al., 2009). H-bond restraints with distance ranges 0-2.2 Å for H...O and 0-3.2 Å for N...O, were included in the secondary structural elements identified in the initial structures and confirmed empirically from the TALOS+ analysis. Structures were calculated from polypeptide chains with randomized φ and ψ torsion angles using a two-stage simulated annealing protocol within the program XPLOR-NIH, essentially as described in (Argentaro et al., 2007).

The structures calculated in XPLOR-NIH were then subjected to a further stage of refinement using a full force field and an implicit water-solvent model as implemented in the program AMBER 11 (Case et al., 2005). Calculations comprised initial minimization (200 steps steepest descent then 1800 steps conjugate gradient), then two rounds of 20 ps of simulated annealing (each comprising 5000 x 1 fs-steps heating from 0 - 500 K; 13000 x 1 fs-steps cooling to 100 K; 2000 x 1fs-steps cooling to 0 K) and final minimization (200 steps steepest descent then 1800 steps conjugate gradient). The experimental distance and torsion angle restraints were applied throughout, and force constants for the distance restraints were increased during the simulated annealing to final values of 20 kcal mol^{-1}Å^{-2} for distance restraints, 20 kcal mol^{-1}rad^{-2} for φ and ψ torsion angle restraints and 50 kcal mol^{-1}rad^{-2} for ω torsion angle restraints. Implicit solvent representation using the generalized Born method (Xia et al., 2002) was employed throughout (igb=1), and Langvin temperature control was used (ntt=3; gamma_ln=5).

The program CLUSTERPOSE (Diamond, 1995) was used to calculate the mean rmsd of ensembles to their mean structures, Ramachandran statistics were evaluated using PROCHECK-NMR (Laskowski et al., 1993; Laskowski et al., 1996) and structures were visualized using the program PyMOL (The PyMOL Molecular Graphics System, Version 1.8 (Schrödinger, LLC, 2015)).

Ciliogenesis rescue experiments

hTERT RPE-1 CEP164 knockout cells (gift from C. Morrison, (Daly et al., 2016)) were cultured in DMEM/F12 supplemented with 10% FBS, 1% L-Glutamine (all from ThermoFisher), and 1× penicillin/streptomycin (Biosera). 24h after seeding cells on glass coverslips, they were transfected with 0.5μg of indicated plasmid (3XFLAG-CEP164 WT, 3XFLAG-CEP164 Q11P or 3XFLAG-CEP164 R93W) using Lipofectamine 3000 (ThermoFisher). The culture medium was exchanged 3h after transfection. To induce ciliogenesis, the cells were starved in 0.1% FBS medium 48h after transfection for 24 h. Cells were then fixed by ice-cold MeOH, 2x washed in PBS, blocked in blocking buffer (1% BSA in PBS), and stained with following primary and secondary antibodies: anti-FLAG (mouse, F1804, Sigma), anti-ARL-13B (rabbit, 17711-1-AP, Proteintech) directly labeled anti-CETN1-Alexa Fluor 647 (12794-1-AP, Proteintech labeled using Alexa Fluor™ 647 Antibody Labeling Kit A20186, Invitrogen), anti-mouse Alexa Fluor 488 (R37114, Invitrogen), anti-rabbit Alexa Fluor 568 (A10042, Invitrogen). Prior to the incubation with anti-CETN1 Alexa Fluor 647, blocking with normal rabbit IgG (sc-2027, Santa Cruz Biotechnology) was done. Coverslips were mounted using Glycergel (Agilent).

Microscopy analysis was done on DeltaVision Elite microscope (GE Healthcare) with a 100×/Zeiss Plan-ApoChromat 1.4 objective and DeltaVision softWoRx acquisition software. Flag-positive cells as well as control untransfected cells were counted and inspected for ARL-13B signal. The image stacks were deconvolved with three cycles of conservative ratio deconvolution algorithm using softWoRx, and directly projected as maximal intensity images.

QUANTIFICATION AND STATISTICAL ANALYSIS

For ITC, dissociation constant (K_D) data are presented as mean ±SD of n independent experiments, as indicated in Table 1. For Western blot experiments, band intensity was quantified in ImageJ software (Schneider et al., 2012). Band intensity data are presented as mean ±SD of n=3 independent replicates (Figures 3D, 3F, S1A, and S1B). Statistical significance between WT and mutants was analysed by a paired, two-tailed t-test in Microsoft Excel (Figure 3F). For ciliogenesis rescue assays (Figure 3H), data are presented as mean percentage of ciliated cells ±SEM. Statistical significance between the groups was analysed by one way ANOVA with Holm-Sidac post-hoc test using GraphPad Prism Software v. 6.0 (GraphPad Software; www.graphpad.com) from n=5 independent experiments, with N=10-20 cells per condition (total number of cells: N_{WT}=66, N_{Q11P}=58, N_{R93W}=55, and $N_{untransfected}$=57).

Structure, Volume *30*

Supplemental Information

Molecular mechanisms underlying the role of the centriolar CEP164-TTBK2 complex in ciliopathies

Ivan Rosa e Silva, Lucia Binó, Christopher M. Johnson, Trevor J. Rutherford, David Neuhaus, Antonina Andreeva, Lukáš Čajánek, and Mark van Breugel

Supplementary Figure 1. Key residues in the CEP164-TTBK2 interface are essential for the CEP164-TTBK2 interaction. A) The N-terminal domain of CEP164 and TTBK2$^{1074\text{-}1087}$ are sufficient for the CEP164-TTBK2 interaction. Western blot showing a pull-down experiment with recombinant GST or GST-CEP164$^{1\text{-}109}$ and lysates from cells expressing C-terminally GFP-tagged TTBK2$^{1074\text{-}1087}$ constructs. TTBK2 constructs carried alanine mutations of four consecutive residues in the 1074-1087 residue range, as indicated. Below the blot, the band intensities of the eluates (in percent of the WT level) are shown (±standard deviation, n=3). **B)** Interface residues of the CEP164 WW domain are critical for its interaction with TTBK2. Western blot showing a pull-down experiment with lysates from cells expressing 3xHA tagged TTBK2 and 3xFLAG tagged CEP164, WT or the indicated WW domain mutants. Below the blot, the band intensities of the eluates (in percent of the WT level) are shown (±standard deviation, n=3). **C)** TTBK2 residues 1076-1078 are crucial for its interaction with CEP164. Western blot showing a pull-down experiment with lysates from cells expressing 3xFLAG tagged CEP164 and 3xHA tagged TTBK2, WT or R1076A+P1077A+P1078A (3-5A) mutant. TCL: Total cell lysate. **D)** The recombinant CEP164$^{1\text{-}109}$ mutants assayed for TTBK2$^{1074\text{-}1087}$ binding in ITC experiments (Figure 1C) do not strongly compromise the CEP164$^{1\text{-}109}$ secondary structure. Buffer-subtracted circular dichroism (CD) spectra of recombinant CEP164$^{1\text{-}109}$, WT and mutants at 25 °C. **E-H)** Coomassie blue-stained 4-12% NuPAGE (Invitrogen) SDS-PAGE gels of **E)** purified, recombinant Nanobodies 10Z, 36Z and the TTBK2$^{1074\text{-}1087}$-CEP164$^{1\text{-}109}$ fusion - Nanobody 36Z complex used for crystallization, **F)** purified, recombinant GST or GST-CEP164$^{1\text{-}109}$ (GST-CEP164-NTD), wild-type (WT) and mutants, as indicated above the gel, that were used in the pull-down experiments in Figure 3B, **G)** purified, recombinant GST or GST-CEP164-NTD, WT, Q11P or R93W that were used in the pull-down experiments in Figure 3F, **H)** purified, recombinant CEP164$^{1\text{-}109}$ (CEP164-NTD), WT and mutants, used in ITC experiments (Figure 1C), **I)** SDS-PAGE (home-made, 15%) gel showing purified, recombinant CEP164$^{1\text{-}109}$-ΔWW and CEP164$^{1\text{-}109}$-ΔHelices that were used in the CD experiments in Figure 2D.

Supplementary Figure 2. The high-resolution structures obtained from two different nanobody-CEP164$^{1\text{-}109}$ complexes are highly similar. A) Top: Ribbon representation of the structure of CEP164$^{1\text{-}109}$, rainbow-coloured from N- to C-terminus, in complex with the camelid nanobody 10Z (in grey). Rotation, as indicated. Consecutive alpha-helices (α), beta-sheets (β) and linkers (L) are labelled. Dotted boxes indicate the regions showed magnified in the panels below. Bottom left: Detailed view of the CEP164$^{1\text{-}109}$ WW domain region including the short β1 strand. Bottom right: Detailed view of the interface region between the CDR3 loop of the bound nanobody and CEP164$^{1\text{-}109}$. Green or yellow dotted lines indicate hydrogen-bonds. Selected residues are shown as sticks and are labelled. **B)** Equivalent views to A) but with the structure of the CEP164$^{1\text{-}109}$-nanobody 36Z complex. Note that despite CDR3 loops of different length and sequence, the binding modes of nanobody 10Z and 36Z are similar to each other. Both nanobodies utilise an arginine in their CDR3 loops to engage CEP164 residue D89. CEP164 D89 is located in the interface region between the WW domain and the helical bundle of CEP164's N-terminal domain.

Supplementary Figure 3. NMR analyses of CEP164$^{1\text{-}109}$ alone and in complex with TTBK2$^{1074\text{-}1087}$ in solution. A) Amino acid residues 1-25 of CEP164$^{1\text{-}109}$ are flexible in solution. Top: {^{1}H} ^{15}N heteronuclear NOE (mean from duplicate datasets) plotted by residue number for CEP164$^{1\text{-}109}$ wild-type (WT, blue circles) and Q11P mutant (red

squares), depicting the flexibility in the CEP164$^{1-109}$ backbone at 293 K. The observed WT - Q11P difference for each residue is shown in black bars. The dotted line across the plot indicates an (arbitrary) threshold with values above indicating conformationally more constrained and values below indicating more flexible backbones. Bottom: RCI-S^2 predicted order parameters obtained from NMR backbone chemical shifts of wild-type CEP164$^{1-109}$ using the TALOS+ software package. RCI-S^2 closely correlates with amplitudes of backbone motions in proteins (Berjanskii and Wishart, 2005). Right: Ribbon presentation of the crystal structure of CEP164$^{1-109}$ (in grey. Residues 1 and 104-109 were not visible in the electron density map, probably due to their high flexibility), consecutive alpha-helices (α), beta-sheets (β) and linkers (L) are labelled. Coloured from green through to red are residues with the specified ranges of ^{1}H ^{15}N heteronuclear NOE (I/I_0) values indicating increased flexibility of the corresponding backbones. **B)** TTBK2$^{1074-1087}$ binding results in only small conformational changes in the N-terminal domain of CEP164. Ribbon representation of the structure of CEP164$^{1-109}$ (in red) overlayed with the structure of the CEP164$^{1-109}$-TTBK2$^{1074-1087}$ complex (in green and blue). Consecutive alpha-helices (α), beta-sheets (β) and linkers (L) of CEP164$^{1-109}$ are labelled. **C)** TTBK2$^{1074-1087}$ binding to CEP164$^{1-109}$ involves the same CEP164 regions *in crystallo* and in solution. Heatmap depicting the extent of exchange broadening in the HSQC of 90 μM ^{15}N-CEP164$^{1-109}$ upon addition of 60 μM unlabelled TTBK2$^{1074-1087}$ at 293 K. The percentage attenuation of peak height for each residue, relative to wild-type, is colour-coded onto the crystal structure of CEP164$^{1-109}$ using a colour gradient from yellow (no peak height attenuation upon TTBK2$^{1074-1087}$ addition), through orange, to red (100% reduction of peak height). Proline residues, which do not produce ^{1}H-^{15}N HSQC peaks, are coloured in grey. The CEP164$^{1-109}$ structure is shown in the same orientation as in B) but in a combined ribbon and semi-transparent surface presentation. Rotation, as indicated. Note the agreement between the CEP164 regions that show exchange broadening upon TTBK2 binding in solution and those *in crystallo* that either are directly involved in TTBK2 binding or undergo conformational changes upon binding (compare to B)).

Supplementary Figure 4. Evolutionary conservation of the solvent exposed WW domain surface and the interior of the CEP164$^{1-109}$ helical bundle. Top: Ribbon representation of the structure of CEP164$^{1-109}$ coloured according to ConSurf (Ashkenazy et al., 2016) conservation scores from variable (cyan) to conserved (burgundy). Consecutive alpha-helices (α), beta-sheets (β) and linkers (L) are labelled. Rotation, as indicated. Dotted boxes indicate the regions showed magnified in the panels below. Bottom left: Detailed view of the solvent exposed CEP164$^{1-109}$ WW domain surface comprising strands β1-β4. Bottom right: Detailed view of the helical bundle region of CEP164$^{1-109}$. Selected residues are shown as sticks and are labelled.

Supplementary Figure 5. Structural comparison of the CEP164$^{1-109}$-TTBK2$^{1074-1087}$ complex with the WW domain-proline-rich binding motif (PRBM) complexes of different WW domain classes. A) View onto the solvent exposed, PRBM-interacting surface of the WW domains of CEP164$^{1-109}$ (green). Left: As ribbon representation and in complex with TTBK2$^{1074-1087}$ (grey). Right: as molecular surface representation. Circled are the XP and the XP2 grooves into which TTBK2 P1077-P1078 and P1075 insert, respectively. **B)** Similar view as in A) but with the WW domain-PRBM complexes of the

indicated members of WW domain classes I, II, III and IV, in ribbon representation. Selected residues are labelled and shown as sticks.

Supplementary Figure 6. The ciliopathic mutation R93W destabilises the CEP164$^{1-109}$ structure. CEP164 contains long regions of predicted disorder.
A) BEST-TROSY NMR spectrum of ^{15}N-labelled CEP164$^{1-109}$ R93W (red) overlayed onto the corresponding wild-type (WT) spectrum (black). Spectra were acquired at a sample temperature of 277 K (left) or 298 K (right). Extensive loss of peaks due to exchange broadening for R93W but not WT at the higher temperature illustrates the structural instability of the mutant. Even at 4°C, perturbations of the TROSY spectrum of the mutant are indicative of a structure that is already compromised. Exchange broadening and peak loss were reversible on re-cooling. **B)** The region between the N-terminal domain and the first coiled coil domain of CEP164 is predicted to be largely unstructured. Per-residue disorder probabilities of CEP164 as calculated by the disorder prediction algorithm AUCpreD (Wang et al., 2016). Values above 0.5 (dashed line) indicate disorder. The location of the N-terminal domain and the predicted coiled coil domains (cc) of CEP164 are indicated above the plot.

Supplementary Figure 7. CEP164 binding inhibits EB1 engagement by TTBK2 and does not stimulate TTBK2's autophosphorylation activity. A) CEP164-NTD binding inhibits full-length TTBK2 association with EB1. Western blot showing a pull-down experiment with bead-bound 3xFLAG or 3xFLAG-EB1 and cell lysates containing 3xHA-tagged full-length TTBK2 (WT or CEP164 binding-deficient ΔPro-rich (Δ1074-1087) mutant), in the presence of recombinant TTBK2-binding-competent WT or -incompetent Y73A GST-CEP164$^{1-109}$, as indicated. TCL: Total cell lysate. **B)** Excess EB1-GFP does not efficiently compete with CEP164$^{1-109}$ (CEP164-NTD) binding to MBP-TTBK2$^{1033-1087}$. Coomassie-stained SDS-PAGE gel showing pull-down experiments with purified MBP-TTBK2$^{1033-1087}$ (wild-type, EB1-binding deficient TTBK2$^{1051-1054\ \mathrm{SKIP\ to\ AAAA}}$ or CEP164-NTD-binding deficient TTBK2$^{1076-1078\ \mathrm{RPP\ to\ AAA}}$) with CEP164$^{1-109}$ (CEP164-NTD) in the presence of an excess of EB1-GFP. **C)** TTBK2 kinase activity is not increased in the presence of an excess of CEP164$^{1-109}$. Coomassie stained SDS-PAGE gel showing a TTBK2 autophosphorylation assay with recombinantly produced TTBK2 in the presence of CEP164-NTD WT or Y73A. Autophosphorylation of wild-type TTBK2, but not its kinase dead D163A mutant, is evident from the slowed TTBK2 migration in Coomassie-stained 8%, low bis-acrylamide SDS-PAGE gels. CEP164-NTD runs with the dye front.

Figure S1

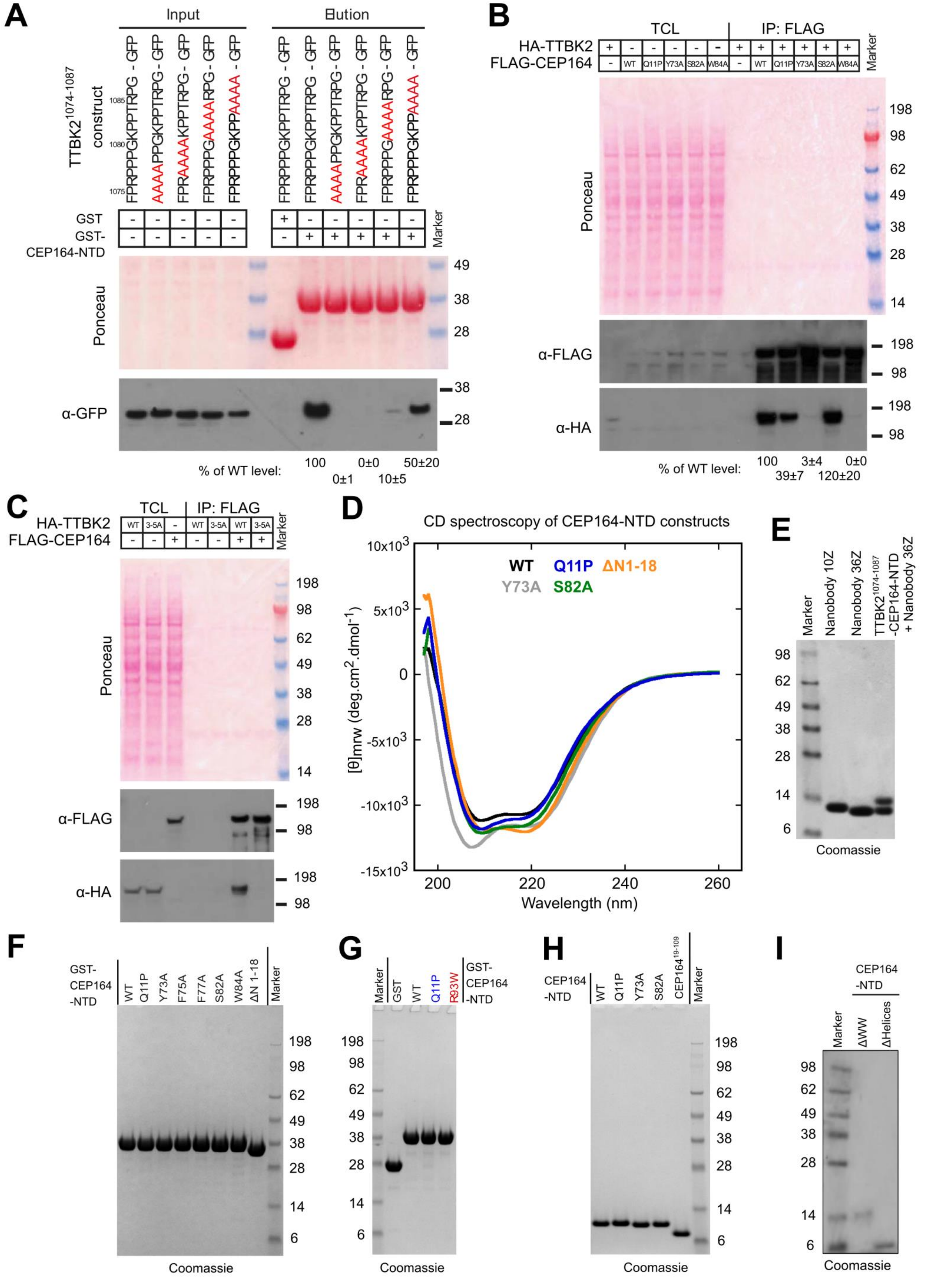
A
Input
Elution
TTBK2$^{1074-1087}$ construct
GST
GST-CEP164-NTD
Marker
Ponceau
α-GFP
% of WT level:
100
0±1
0±0
10±5
50±20
B
TCL
IP: FLAG
HA-TTBK2
FLAG-CEP164
WT
Q11P
Y73A
S82A
W84A
Marker
Ponceau
α-FLAG
α-HA
% of WT level:
100
39±7
3±4
120±20
0±0
C
TCL
IP: FLAG
HA-TTBK2
FLAG-CEP164
3-5A
Ponceau
α-FLAG
α-HA
D
CD spectroscopy of CEP164-NTD constructs
WT
Q11P
ΔN1-18
Y73A
S82A
[θ]mrw (deg.cm^2.dmol^{-1})
Wavelength (nm)
E
Marker
Nanobody 10Z
Nanobody 36Z
TTBK2$^{1074-1087}$-CEP164-NTD + Nanobody 36Z
Coomassie
F
GST-CEP164-NTD
WT
Q11P
Y73A
F75A
F77A
S82A
W84A
ΔN 1-18
Marker
Coomassie
G
Marker
GST
WT
Q11P
R93W
GST-CEP164-NTD
Coomassie
H
CEP164-NTD
WT
Q11P
Y73A
S82A
CEP164$^{19-109}$
Marker
Coomassie
I
CEP164-NTD
Marker
ΔWW
ΔHelices
Coomassie

Figure S2

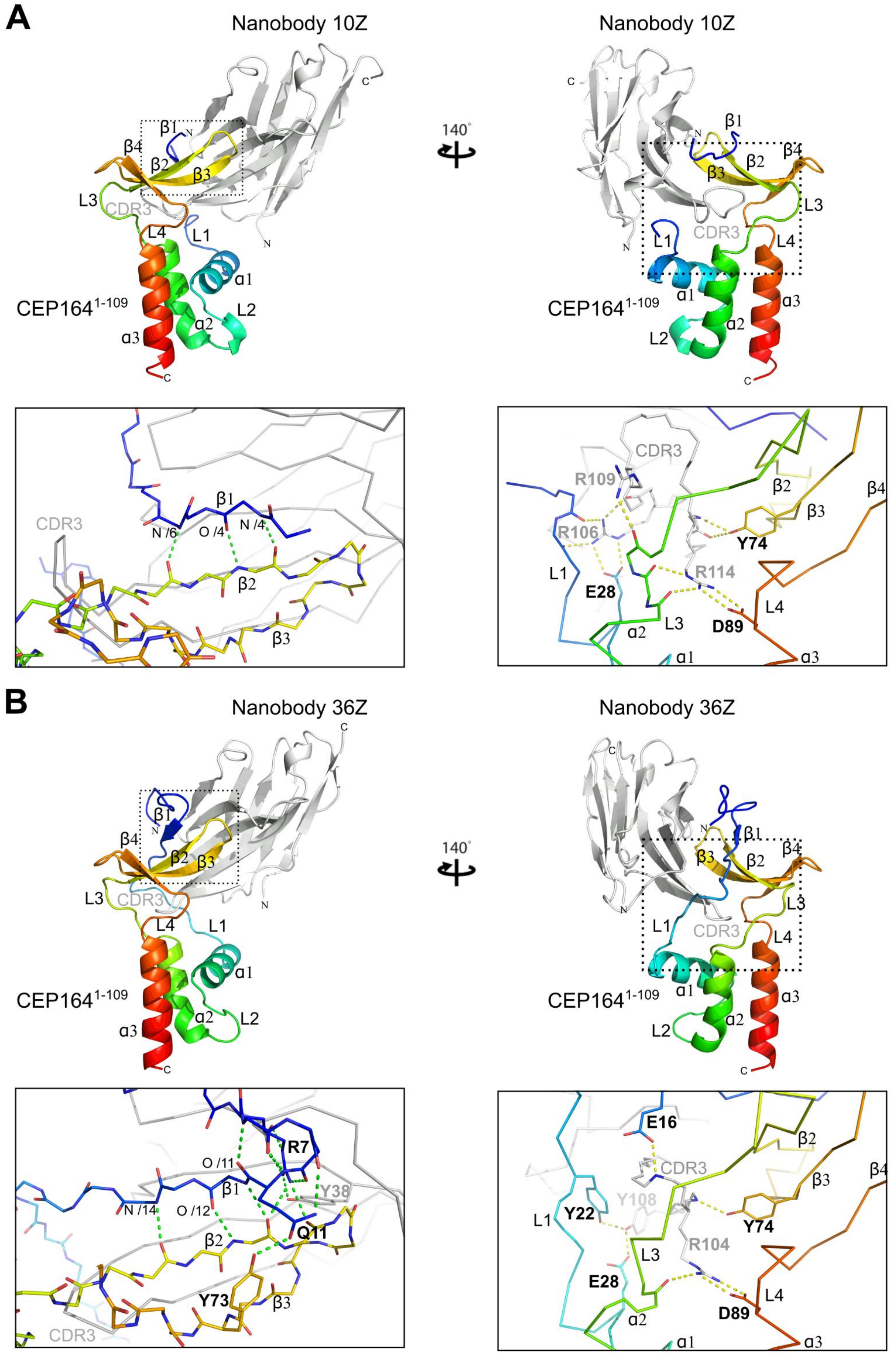
A
Nanobody 10Z
Nanobody 10Z
140°
β1
β2
β3
β4
L1
L2
L3
L4
CDR3
α1
α2
α3
CEP164^1-109
N
C
N /6
O /4
N /4
R109
R106
R114
E28
Y74
D89
B
Nanobody 36Z
Nanobody 36Z
140°
O /11
O /12
N /14
R7
Y38
Q11
Y73
E16
Y22
Y108
R104

Figure S3

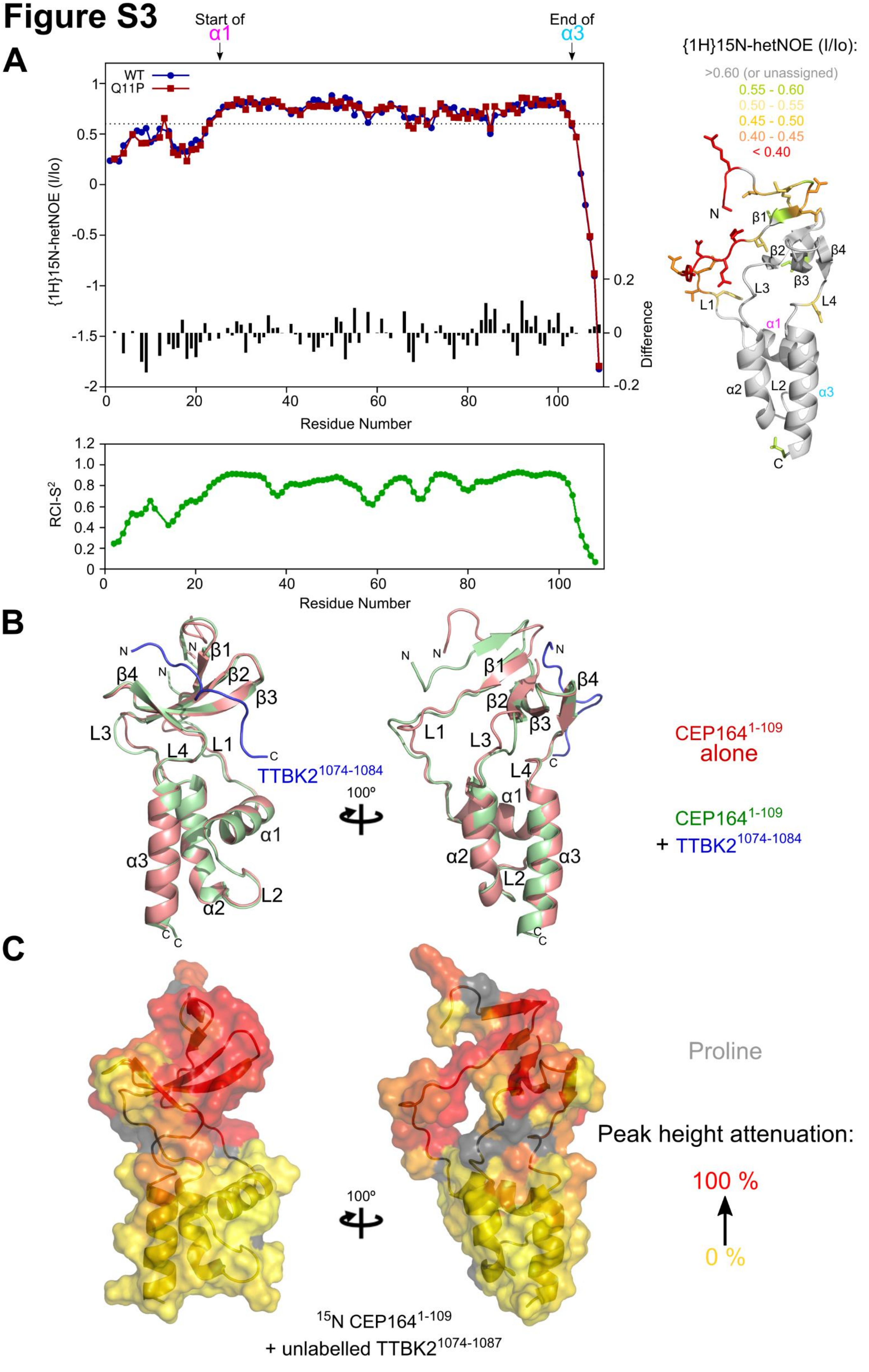
A
Start of α1
End of α3
WT
Q11P
{1H}15N-hetNOE (I/Io)
Difference
Residue Number
RCI-S^2
{1H}15N-hetNOE (I/Io):
>0.60 (or unassigned)
0.55 - 0.60
0.50 - 0.55
0.45 - 0.50
0.40 - 0.45
< 0.40
B
TTBK2$^{1074-1084}$
100º
CEP164$^{1-109}$ alone
CEP164$^{1-109}$ + TTBK2$^{1074-1084}$
C
100º
Proline
Peak height attenuation:
100 %
0 %
^{15}N CEP164$^{1-109}$
+ unlabelled TTBK2$^{1074-1087}$

Figure S4

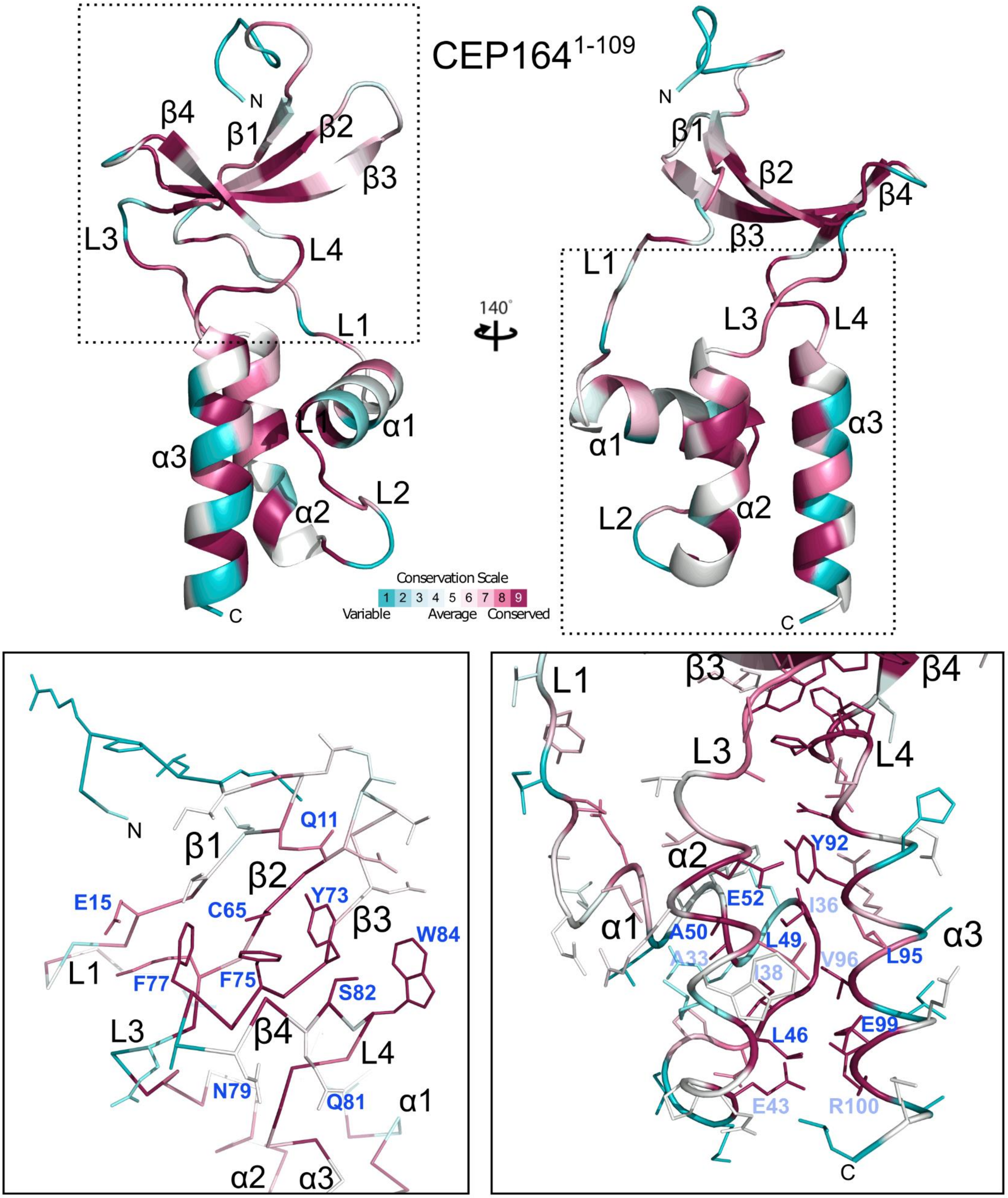

CEP164^1-109
N
β4
β1
β2
β3
L3
L4
L1
α1
α3
α2
L2
C
140°
Conservation Scale
1 2 3 4 5 6 7 8 9
Variable
Average
Conserved
Q11
E15
C65
Y73
W84
F77
F75
S82
N79
Q81
Y92
E52
I36
A50
L49
A33
I38
V96
L95
E99
L46
E43
R100

Figure S5

A

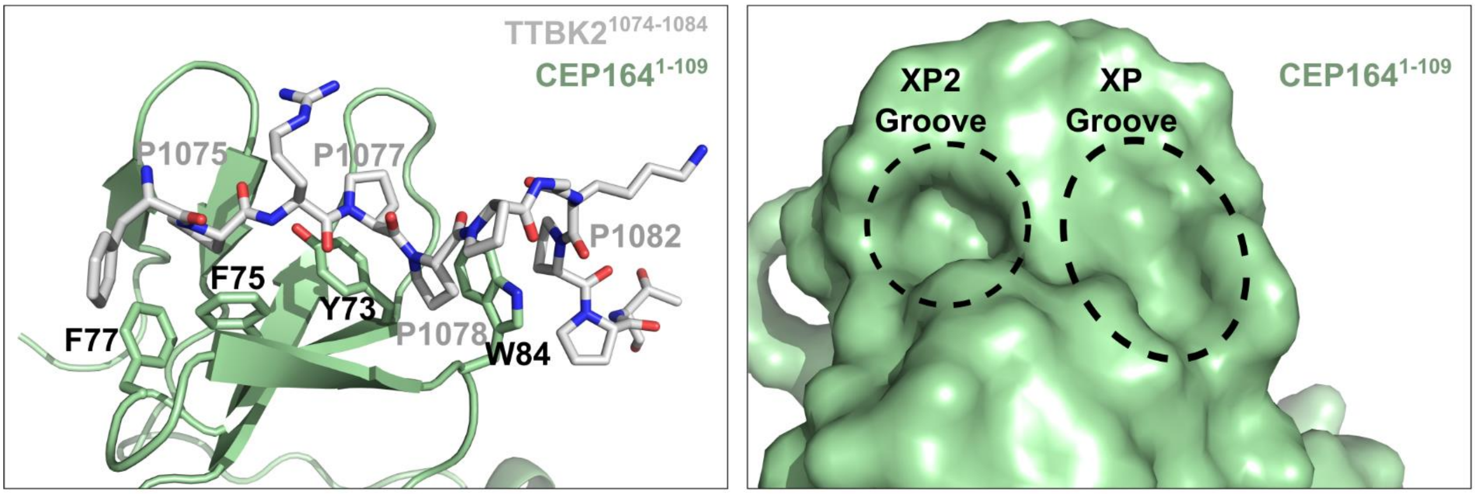

B

Class I WW domain

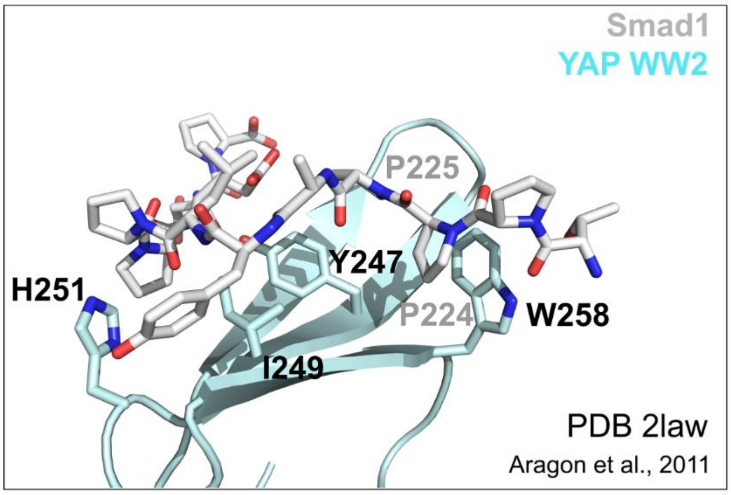

Class II WW domain

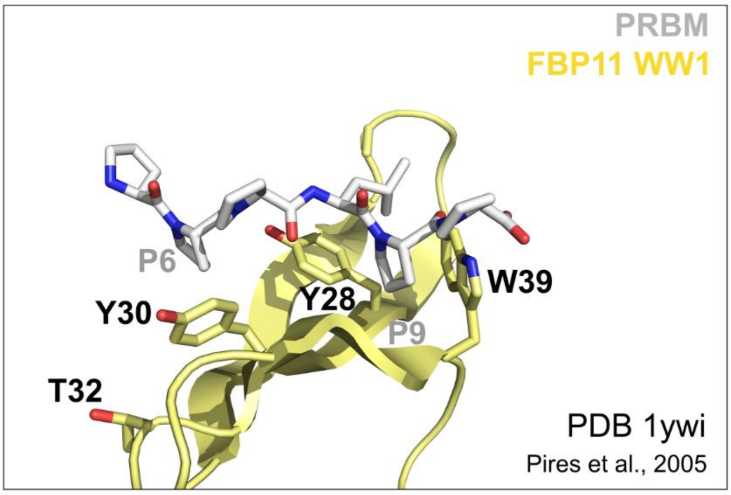

Class III WW domain

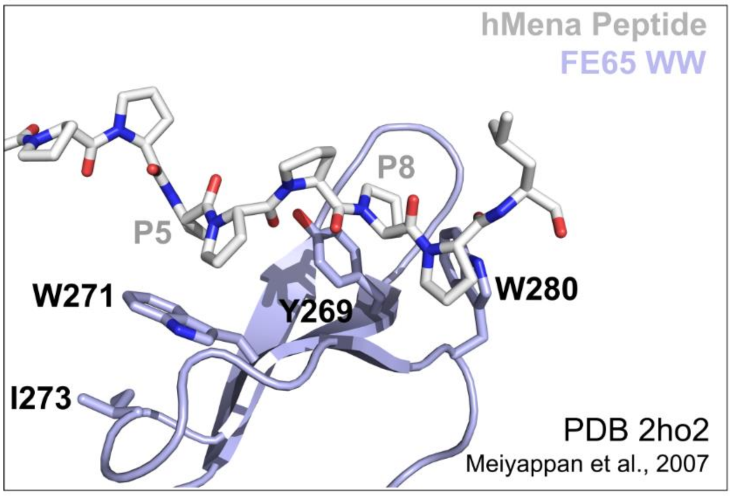

Class IV WW domain

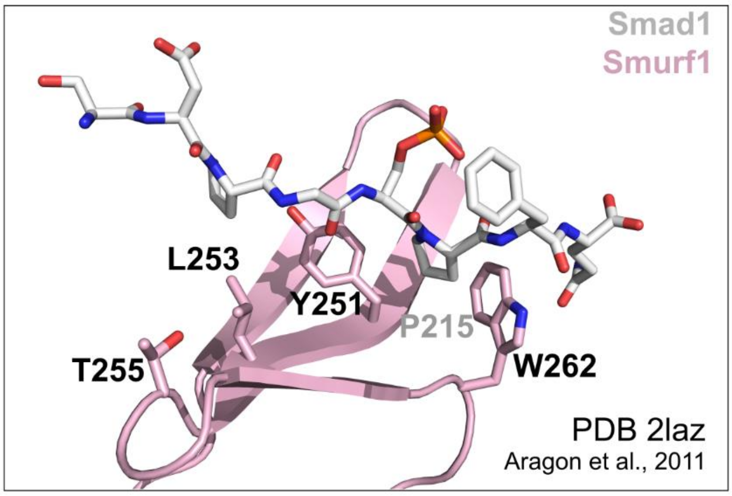

Figure S6

A

NMR BEST-TROSY spectra of ^{15}N labelled CEP164$^{1-109}$

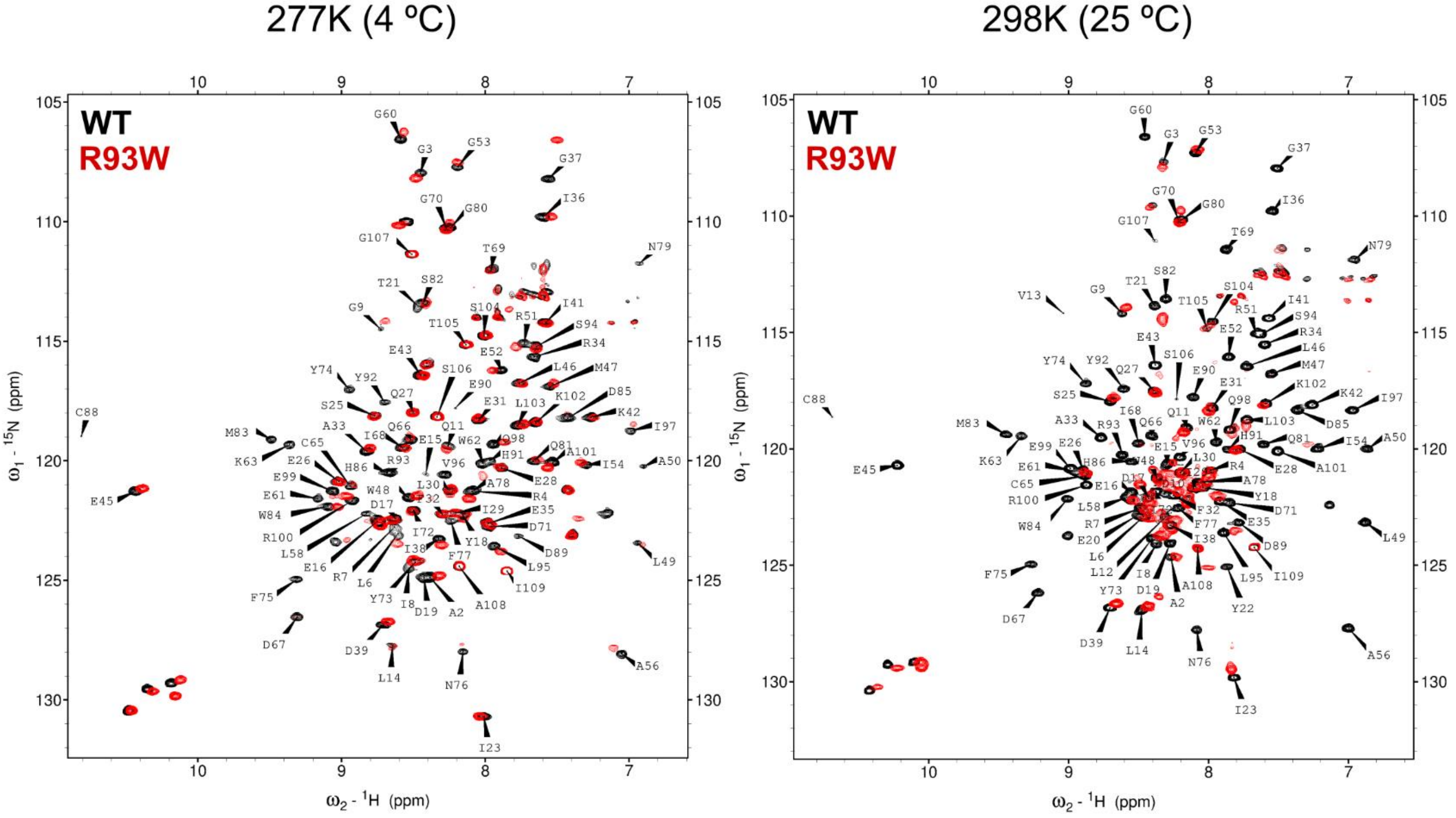

B

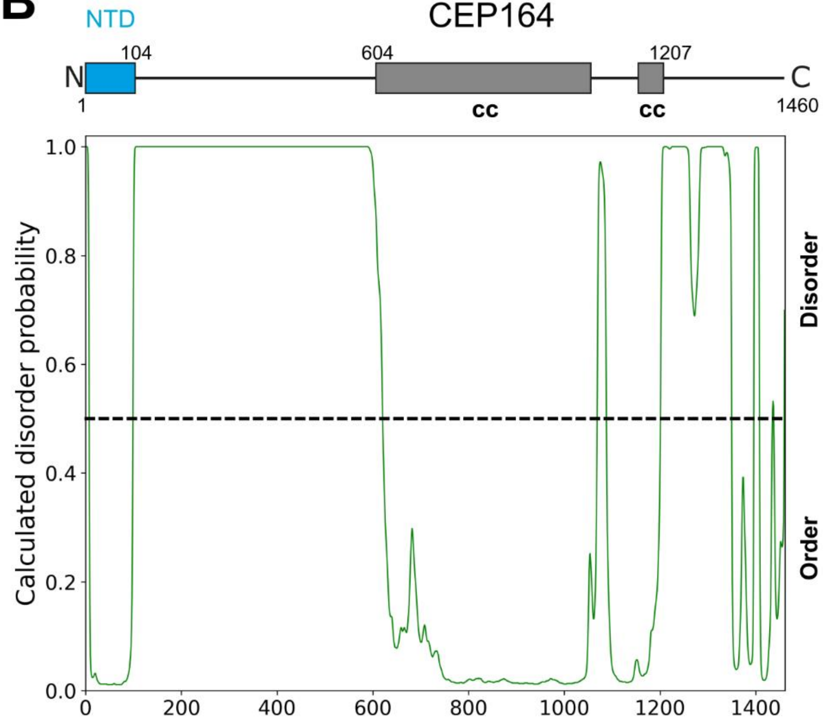

Figure S7

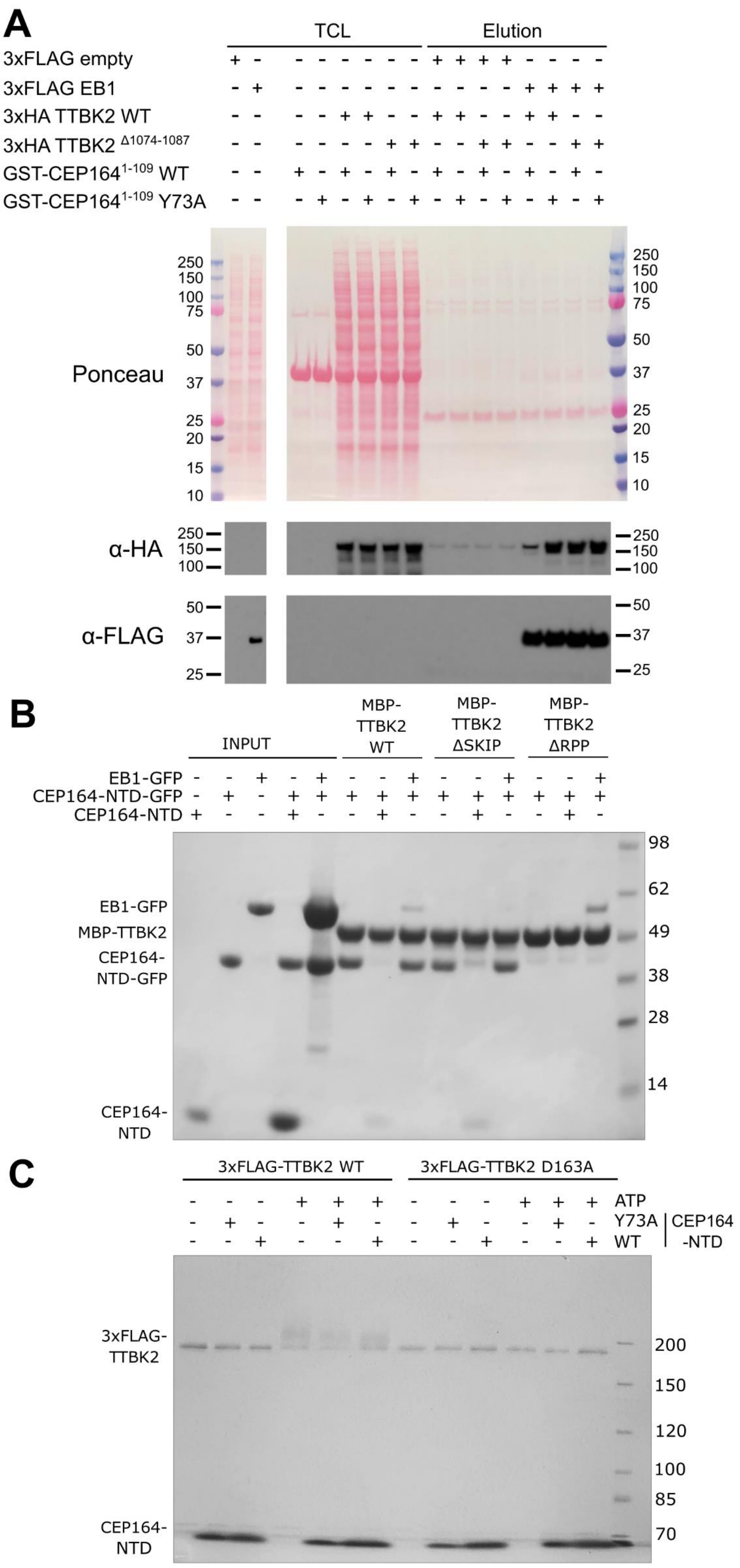
A
TCL
Elution
3xFLAG empty
3xFLAG EB1
3xHA TTBK2 WT
3xHA TTBK2 Δ1074-1087
GST-CEP164 1-109 WT
GST-CEP164 1-109 Y73A
Ponceau
α-HA
α-FLAG
B
INPUT
MBP-TTBK2 WT
MBP-TTBK2 ΔSKIP
MBP-TTBK2 ΔRPP
EB1-GFP
CEP164-NTD-GFP
CEP164-NTD
MBP-TTBK2
C
3xFLAG-TTBK2 WT
3xFLAG-TTBK2 D163A
ATP
Y73A
WT
CEP164 -NTD
3xFLAG-TTBK2
CEP164-NTD
